# Structural basis for membrane-dependent positive feedback regulation of DOCK11 by activated Cdc42

**DOI:** 10.1073/pnas.2607665123

**Published:** 2026-10-06

**Authors:** Tamao Hisano, Takehiro Shinoda, Mutsuko Kukimoto-Niino, Kazushige Katsura, Ryohei Kondo, Yoshiko Ishizuka-Katsura, Yukako Miyata-Yabuki, Kazuharu Hanada, Reiko Nakagawa, Akihiko Nishikimi, Mikako Shirouzu

**Affiliations:** ^a^ Laboratory for Protein Functional and Structural Biology, RIKEN Center for Integrative Medical Sciences, Yokohama 230-0045, Kanagawa, Japan; ^b^ Core Facility Administration, Research Institute, National Center for Geriatrics and Gerontology, Obu 474-8511, Aichi, Japan; ^c^ Mass Spectrometry Analysis Team, RIKEN Center for Biosystems Dynamics Research, Kobe 650-0047, Hyogo, Japan; ^d^ Structural Life Science and Cell Biology Collaboration Team, RIKEN Center for Biosystems Dynamics Research, Yokohama 230-0045, Kanagawa, Japan

**Keywords:** Rho GTPase signaling, RhoGEF, cryo-EM, positive feedback, membrane association

## Abstract

The guanine nucleotide exchange factor DOCK11 is widely expressed across tissues and activates Cdc42, a Rho family GTPase that regulates cytoskeletal remodeling and cell polarity. Loss-of-function mutations in DOCK11 have recently been linked to inborn errors of immunity. DOCK11 has also been implicated in facilitating hepatitis B virus infection in hepatocytes and has emerged as a potential target for therapeutic interventions. A positive feedback mechanism has been proposed for DOCK11-mediated Cdc42 activation; however, the structural basis for this regulation has remained unclear. Here we report the cryoelectron microscopy structures of full-length DOCK11 as an apo dimer, a binary complex with nucleotide-free Cdc42, and a ternary complex containing both nucleotide-free and activated Cdc42. DOCK11 predominantly adopts a U-shaped dimer that binds to nucleotide-free Cdc42 via the catalytic domain, whereas activated Cdc42 engages a distal site within the armadillo-repeat domain. Biochemical and cellular analyses demonstrate that binding of activated Cdc42 is critical for enhancing the membrane-dependent nucleotide exchange activity of DOCK11, promoting its localization to the plasma membrane, and facilitating intracellular activation of Cdc42. We also determined the structure of the membrane-bound ternary complex using membrane-coated grids. Strikingly, the ternary complex adopts an extended conformation that aligns multiple membrane-contact elements, revealing how activated Cdc42 stabilizes DOCK11 membrane binding for efficient exchange activity. Collectively, these findings provide a structural framework for the coordinated mechanism underlying membrane-dependent positive feedback in Rho GTPase signaling.

## Significance

Cdc42 is a small GTPase that acts as a molecular switch controlling cell polarity, migration, and immune responses. Positive feedback has been proposed to contribute to DOCK11-mediated Cdc42 activation, but how this process occurs at the membrane has remained unclear. We determined the structures of full-length DOCK11 in multiple functional states, including its membrane-bound form. Our results reveal how interactions with membranes and activated Cdc42 cooperate to sustain DOCK11 activity and amplify Cdc42 signaling. These findings provide a structural framework for understanding how membrane-associated regulators fine-tune intracellular signaling pathways.

Rho GTPases are central regulators of cytoskeletal dynamics that influence numerous cellular processes, including morphogenesis, polarity, motility, and cell division (1). Dedicator of cytokinesis (DOCK) proteins are evolutionarily conserved guanine nucleotide exchange factors (GEFs) for the Rho GTPases Rac and Cdc42, which promote their conversion from GDP- to GTP-bound states (2, 3). In mammals, 11 DOCK proteins activate Rac and Cdc42 to control a broad spectrum of developmental and immune functions under normal and pathological contexts (4, 5). Based on sequence homology, they are grouped into four subfamilies (A to D) and share four conserved domains: three DOCK homology regions (DHR1–DHR3; DHR3 was previously referred to as the C2 domain) and armadillo repeats (the ARM domain) (6). The DHR1 domain binds phosphoinositides (PIPs) (7), whereas the DHR2 domain is responsible for GTPase activation and mediates homodimerization (2, 8). Among the DOCK proteins, the DOCK-A subfamily (DOCK1, 2, and 5) has been comparatively well characterized with respect to functions, regulatory mechanisms, and structures. Structural works using cryoelectron microscopy (cryo-EM) and crystallography have established the ELMO1-dependent autoinhibitory mechanism and shown that interactions with partners such as RhoG and BAI1 relieve this autoinhibition and promote plasma-membrane localization (9–11).

DOCK11 belongs to the DOCK-D subfamily which also shares a pleckstrin homology (PH) domain at the N-terminal region (2). DOCK11 specifically activates Cdc42 (12) and is widely expressed across various tissues including leukocytes, spleen, bone marrow, thymus, lungs, and placenta (13). In these cells, DOCK11 is found not only in the cytoplasm but also in the nucleus (14). Notably, it is involved in immune responses, participating in B cell homeostasis and differentiation (15–21). Loss-of-function mutations in DOCK11 cause inborn errors of immunity characterized by immune dysregulation, immunodeficiency, and anemia (22, 23). Studies using patient-derived cells and knockout models have revealed that DOCK11 deficiency leads to abnormal T cell morphology and migration, impaired cytokine production in both T and B cells, and erythroid hypoplasia (22, 23). DOCK11 has also been reported to facilitate hepatitis B virus (HBV) infection by promoting viral nuclear entry through retrograde trafficking in hepatocytes (24). Furthermore, DOCK11 accumulates at DNA damage sites and promotes actin polymerization to elicit repair pathways, which are crucial for the formation of covalently closed circular DNA (cccDNA), thereby enabling HBV persistence and reactivation (25, 26). Consequently, DOCK11 represents a potential therapeutic target for HBV treatment.

Lin et al. (12) reported that the GEF activity of DOCK11 is accelerated by the binding of activated Cdc42 to DOCK11, suggesting a positive feedback mechanism that regulates the intracellular signaling associated with DOCK11. To elucidate the positive feedback mechanism of DOCK11, we determined the cryo-EM structures of full-length DOCK11 and its complex with inactive and activated Cdc42. We showed that DOCK11 exhibits different conformations in solution and on the membrane. Based on biochemical and cell imaging analyses, we propose a mechanism by which DOCK11 regulates Cdc42 activation by binding to activated Cdc42 on the membrane.

## Results

### Cryo-EM Structure of DOCK11.

**Fig. 1.**
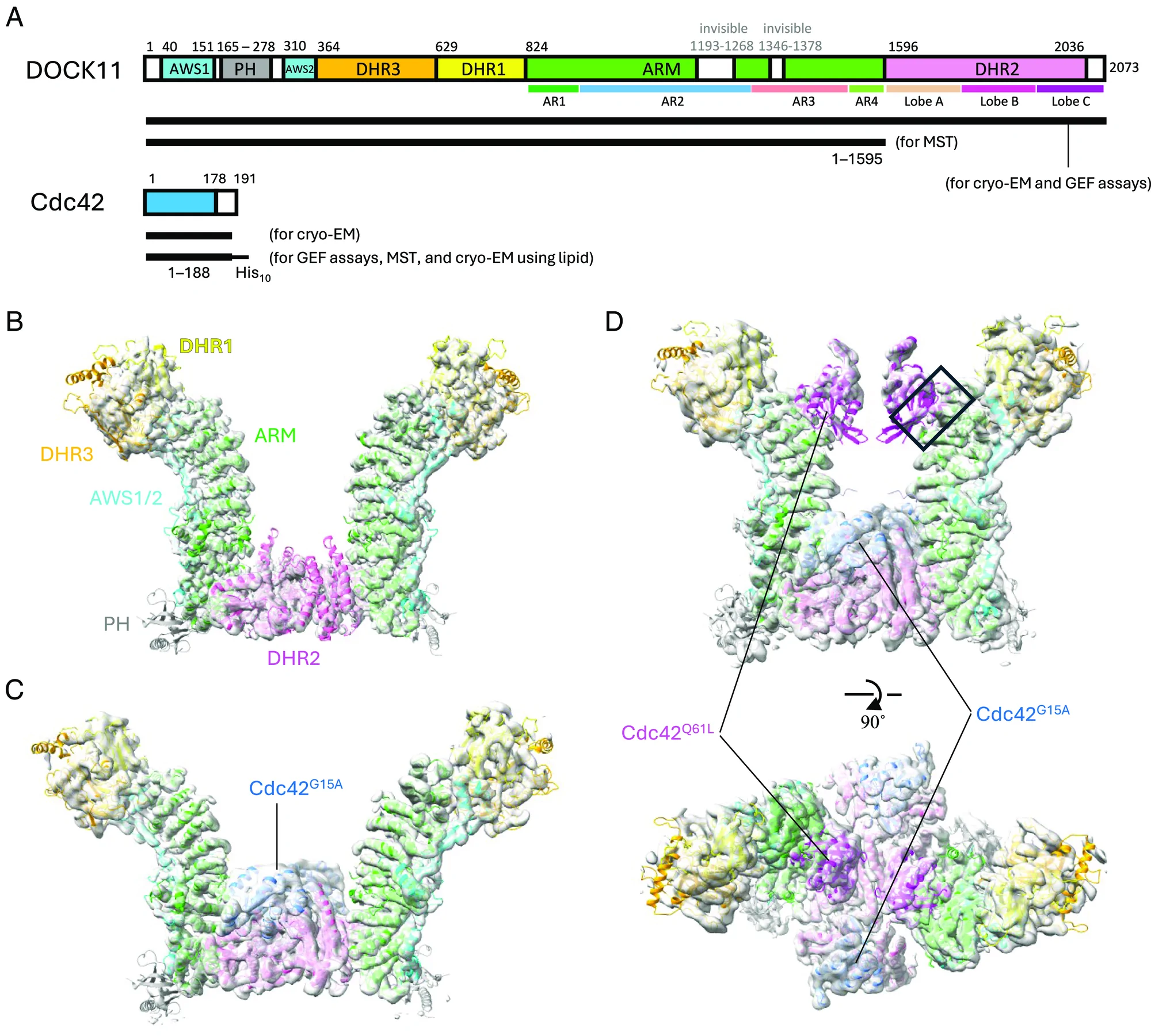
Cryo-EM structures of DOCK11 in solution. (*A*) Schematic of DOCK11 and Cdc42 domain structures. The AWS1, AWS2, PH, DHR3, DHR1, ARM, and DHR2 domains are colored differently. The DHR2 domain has three subdomains: Lobe A (residues 1,596 to 1,758), Lobe B (residues 1,759 to 1,912), and Lobe C (1,923 to 2,073). Regions not visible on the map and not modeled are colored white. Black bars indicate the regions of the protein constructs used in this study. (*B–D*) Cryo-EM density maps with fitted structural models of (*B*) DOCK11, (*C*) DOCK11–Cdc42^G15A^ binary complex, and (*D*) DOCK11–Cdc42^G15A^–Cdc42^Q61L^ ternary complex. DOCCK11 domains are colored as in *A*. Cdc42^G15A^ (the nucleotide-free mutant) and Cdc42^Q61L^ (the constitutively active mutant) are colored sky blue and magenta, respectively. Labels are indicated only for one protomer (Chain A). The rectangle in *D* indicates the activated Cdc42-binding site, which is shown in detail in Fig. 2*A*. Maps are generated using DeepEMhancer (29). The figures of the maps and structural models were generated using ChimeraX (30).

DOCK11 is a large protein with 2,073 residues, composed of five domains (PH, ARM, and DHR1–DHR3) (Fig. 1*A*). The cryo-EM structure of the DOCK11 dimer was determined at an overall resolution of 3.80 Å (Fig. 1*B* and *SI Appendix*, Figs. S1 and S2 *A* and *B* and Table S1). Initial cryo-EM analysis of DOCK11 yielded only low-resolution (5.6 Å) maps (*SI Appendix*, Fig. S3). Therefore, DOCK11 samples were cross-linked with bis(sulfosuccinimidyl)suberate (BS^3^), a widely used *N*-hydroxysuccinimide ester cross-linking agent, and subsequently subjected to cryo-EM analysis, yielding maps at higher resolution. The dimeric DOCK11 molecule exhibits a U-shaped structure with the ARM domains facing each other along the molecular dyad axis. The dimeric association is mediated mainly by hydrophobic interactions between the DHR2 domains (*SI Appendix*, Fig. S2*G*), as observed for other DOCK proteins in full-length (DOCK2, DOCK5, and DOCK6) or truncated forms (DOCK9 and DOCK10) (8–11, 27, 28).

The ARM domain is mainly composed of helices stabilized by stacking interactions and folded into a superhelical structure or α-solenoid. For descriptive purposes, we divided the domain into four regions (AR1–AR4) based on helix orientation (Fig. 1*A* and *SI Appendix*, Fig. S4). The stacking interaction extends to the helices of the DHR2 domain (Lobe A), stabilizing the ARM–DHR2 interaction. The connection between the ARM and DHR2 domains bends approximately 90˚, resulting in the DOCK11 protomer adopting a “L”-like shape. DHR3 and DHR1 domains have β-sandwich structures with the β-strand topology of the type II C2 domain (31) and interact extensively with each other (hereafter referred to as the DHR3-DHR1 tandem). Several basic residues (Lys and Arg) are distributed on one face of the DHR3-DHR1 tandem, which may be involved in interactions with the negatively charged lipid membranes (see below). The DHR3-DHR1 tandem interacts with the α-helices of AR1 (residues 824 to 930) of the ARM domain. This interaction is braced by a short helix (residues 100 to 109) at the interface (*SI Appendix*, Fig. S4*B*). The N-terminal segments (residues 39 to 151 and 301 to 363) bind to the concave surface of the ARM domain spanning the entire longitudinal axis, with most regions adopting an extended main chain conformation (Fig. 1*B* and *SI Appendix*, Fig. S4*B*). We refer to these regions as ARM-wrapping segments 1 (AWS1; residues 39 to 151) and 2 (AWS2; residues 301 to 363). The region (residues 152 to 309) including the PH domain (residues 165 to 278) has poor density, indicating that it is a mobile region. The PH domain has been modeled with an ambiguous orientation near AR4 of the ARM domain, where densities are visible at a low contour level (*SI Appendix*, Fig. S2).

DOCK11 shares structurally conserved domains (ARM and DHR1–DHR3) with DOCK2 and DOCK5, including the tandem structures of DHR1 and DHR3 (C2) domains (6, 9, 10). Helices of the ARM domains of DOCK11 and DOCK5 occupy analogous positions, however, superposition reveals relatively large deviations in AR4 (RMSD of 3.2 Å for 500 Cα atoms of the ARM domain) (*SI Appendix*, Fig. S5*A*). A deviation is also observed in the AR4–DHR2 relative orientation (*SI Appendix*, Fig. S5*B*). These deviations result in conformational differences in the ARM–DHR2 region and opening angle between the ARM and DHR2 domains. Unlike DOCK11, DOCK2 and DOCK5 lack the bracing helix positioned between the DHR3-DHR1 tandem and the ARM domain and instead display a distinct interdomain interaction. In DOCK2 and DOCK5, the helical domain located N-terminal to the DHR3 domain contacts the concave surface of the ARM domain (while also engaging the regulatory protein ELMO1). As a result, the DHR3-DHR1 tandem adopts an orientation relative to the ARM domain that differs from that of DOCK11.

### The ARM Domain Serves as the Binding Site for the Activated Cdc42.

We prepared a sample for a DOCK11–Cdc42^G15A^–Cdc42^Q61L^ ternary complex (the “ternary sample”) and performed cryo-EM analysis (Cdc42^G15A^ and Cdc42^Q61L^ are nucleotide-free and constitutively active mutants, respectively). Three-dimensional (3D) classification of the particles yielded two distinct reconstructed maps (*SI Appendix*, Fig. S6 and Table S1). One, exhibiting extra densities at the DHR2 domain, appeared to correspond to the DOCK11–Cdc42^G15A^ binary complex (4.3 Å resolution), whereas the other, with additional extra densities at the ARM domain, represented the ternary complex (3.8 Å resolution) (Fig. 1 *C* and *D*). Consistently, cryo-EM analysis of the DOCK11–Cdc42^G15A^ binary complex (the “binary sample”) produced a 5.2 Å-resolution map (*SI Appendix*, Fig. S7), which closely resembled that of the binary complex derived from the ternary sample. In the ternary complex structure, the additional density was well fitted by the Cdc42^Q61L^ model, which displayed characteristic features of the Switch I and Switch II regions in the GTP-bound state (e.g., PDB ID 1NF3 chain B), clearly distinct from those observed in nucleotide-free Cdc42 structures. Comparison of the structures of DOCK11 alone, the binary complex, and the ternary complex revealed conformational differences in the relative orientation of the two ARM domains of the DOCK11 dimer, pivoting at the hinge between the ARM and DHR2 domains, indicating substantial structural flexibility (*SI Appendix*, Fig. S8). Furthermore, cross-linking mass spectrometry of the DOCK11–Cdc42^G15A^–Cdc42^Q61L^ ternary complex revealed long-range cross-links between the ARM domain and the DHR2 domain or Cdc42^G15A^, suggesting that the ARM domain is flexible relative to the DHR2 domain–Cdc42^G15A^ (*SI Appendix*, Fig. S9 and Table S3). These data suggest that the ARM domains undergo dynamic fluctuations within the U-shaped DOCK11 dimer. In addition, the same analysis identified cross-links bridging the two Cdc42 molecules bound to the ARM domains within the dimer (*SI Appendix*, Fig. S9). Accordingly, these cross-links may have restricted the relative movements of the ARM domains, and the cryo-EM structure of the cross-linked ternary complex may therefore represent one of the conformational states in which the ARM domains adopt a more closed arrangement.

**Fig. 2.**
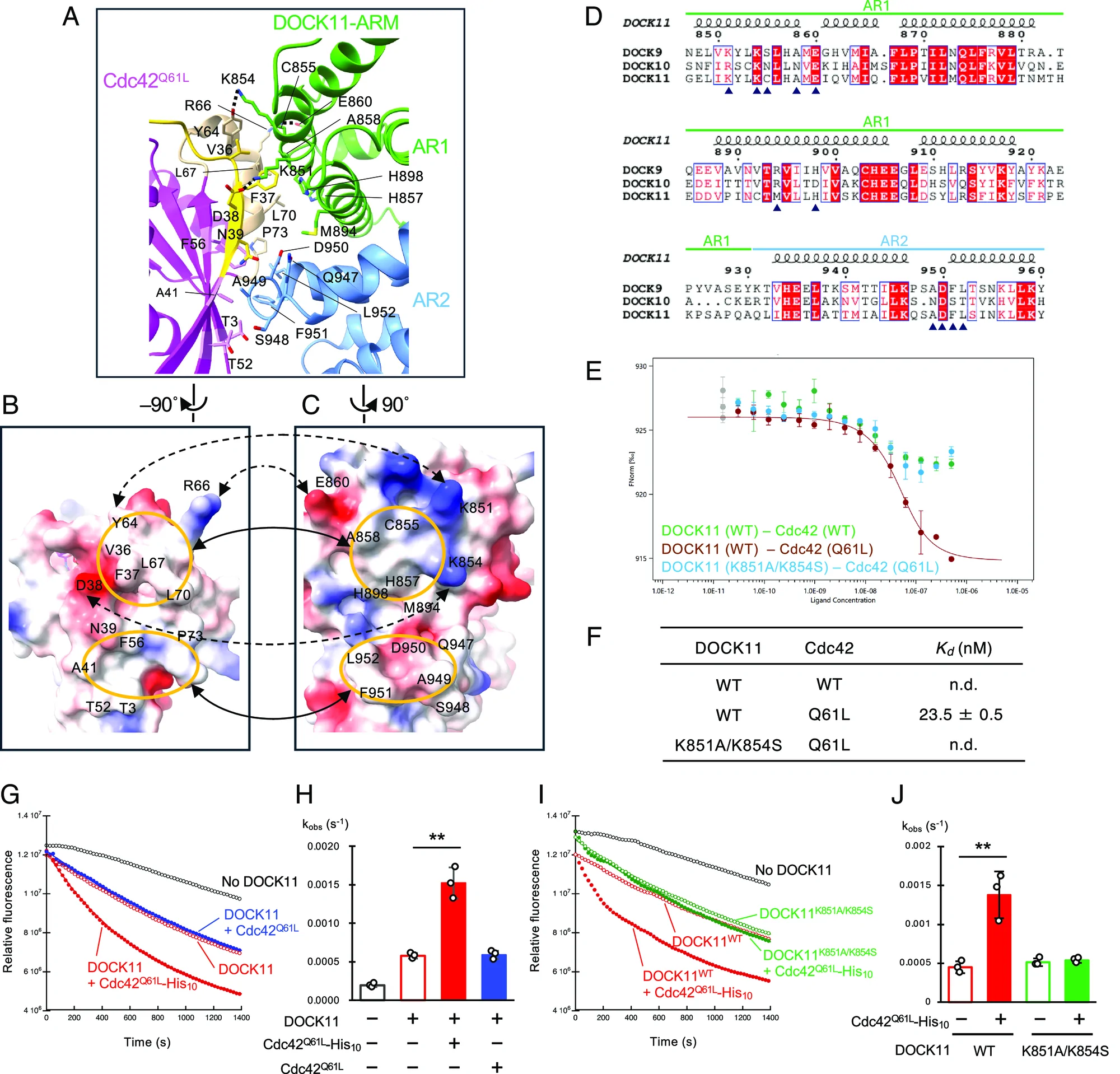
The interaction of activated Cdc42 with the ARM domain. (*A*) Interaction interface between the ARM domain of DOCK11 and activated Cdc42. Residues involved in hydrophobic/van der Waals interactions and possible electrostatic interactions (dashed lines) are shown as sticks. AR1 and AR2 are colored green and light blue, respectively. Cdc42^Q61L^ is colored magenta, with Switch I and II regions shown in gold and tan, respectively. (*B*) Electrostatic surface potential of the ARM-domain binding site viewed after a −90° rotation relative to *A*. (*C*) Electrostatic surface potential of Cdc42^Q61L^ viewed after a 90° rotation relative to *A*. Orange circles indicate interacting surfaces, and dashed arrows indicate possible electrostatic interactions. The figures of the structural models and surface potential maps were generated using PyMOL (32). (*D*) Sequence alignment of DOCK-D family proteins around the activated Cdc42-binding site. Residues located at the Cdc42^Q61L^-binding interface are indicated by triangles. Bars denote the AR1 and AR2 regions. The alignment was generated using Clustal Omega (33) and ESPript3 (34). (*E*) The interaction analyses of Cdc42 [wild-type (WT) or Q61L mutant] with DOCK11 WT (green and red) and double mutant (K851A/K854S; blue) by microscale thermophoresis (MST). Note that DOCK11 has been truncated (1 to 1,595) lacking the DHR2 domain to avoid a potential interaction of Cdc42 with the DHR2 domain. (*F*) Summary of the MST analysis. Data are given as *K_d_* (dissociation constant) ± fitting error derived from a global fit of three independent experiments. (*G–J*) In vitro GEF assays for DOCK11 in the presence of PC/PE/DGS-NTA(Ni)-containing SUVs. (*G* and *I*) Representative fluorescence time courses. (*H* and *J*) Quantification of three technically independent experiments (mean ± SD). (*G* and *H*) Effects of membrane-tethered Cdc42^Q61L^-His_10_ and untethered Cdc42^Q61L^ on the GEF activity of DOCK11. (*I* and *J*) Comparison of the GEF activities of WT and K851A/K854S mutant DOCK11 in the presence or absence of membrane-tethered Cdc42^Q61L^-His_10_. Statistical comparisons were performed using an unpaired two-sided Student’s *t* test (**P < 0.01).

Cdc42^Q61L^ binds the N-terminal region of the ARM domain (AR1 and part of AR2) through a combination of electrostatic and hydrophobic/van der Waals interactions (Figs. 1*D* and 2 *A*–*C* and *SI Appendix*, Fig. S10). A hydrophobic patch formed by Switch I and Switch II residues of Cdc42^Q61L^ (Val36, Phe37, Tyr64, Leu67, and Leu70) lies against a complementary surface on AR1 of DOCK11 involving Cys855, Ala858, Lys854, His857, and His898. In addition, a shallow hydrophobic groove on Cdc42^Q61L^ (Thr3, Asn39, Ala41, Thr52, Phe56, and Pro73) accommodates a protruding loop from DOCK11 (Ala949, Phe951, and Leu952) located between the first and second helices of AR2. Several polar residues are positioned in close proximity across the interface, including Tyr64, Asp38, and Arg66 of Cdc42^Q61L^ and Lys851, Lys854, and Glu860 of DOCK11, suggesting potential electrostatic/hydrogen-bonding interactions that may further stabilize the complex. These interactions ensure the specific recognition of the active form of Cdc42 by DOCK11.

Sequence alignment within the DOCK-D subfamily showed that the 13 residues in DOCK11 that appear to be involved in the interaction with Cdc42^Q61L^ are mostly conserved in DOCK9 but less conserved in DOCK10 (Fig. 2*D*), suggesting that DOCK9 possesses a similar Cdc42-binding site. However, DOCK9 has been reported to be unable to bind the activated Cdc42 (35, 36). This discrepancy may be due to the substitution of Met895 in DOCK11 with Arg in DOCK9. Alternatively, since the N-terminal half of DOCK9 which includes the PH, DHR3, DHR1, and ARM domains has been reported to participate in an autoinhibitory interaction with the DHR2 domain (36), DOCK9 interdomain interactions might sterically inhibit the binding of activated Cdc42 to the ARM domain.

### Validation of Activated Cdc42 Binding to the ARM Domain of DOCK11.

To confirm the direct binding of activated Cdc42 to the ARM domain, we performed microscale thermophoresis (MST) experiments. We expressed and purified a C-terminally truncated DOCK11 protein (residues 1 to 1,595) lacking the DHR2 domain and examined its binding to a fluorescent-labeled Cdc42 protein (Fig. 2 *E* and *F*). The truncated DOCK11 bound to Cdc42^Q61L^ (GTP-bound form) with a K_d_ value of 24 nM but not to WT Cdc42 (GDP-bound form), indicating that activated Cdc42 specifically binds to DOCK11 lacking the DHR2 domain. Simultaneous mutations of two residues in the ARM domain, Lys851 and Lys854 (K851A/K854S), inhibited the binding of truncated DOCK11 to the activated Cdc42 (Fig. 2 *E* and *F*). This indicates that either or both Lys851 and Lys854 contribute to binding to the activated Cdc42, supporting the results of the cryo-EM analysis.

Subsequently, to assess the effect of activated Cdc42 binding on the GEF activity of DOCK11, we performed an in vitro GEF activity assay using small unilamellar vesicles (SUVs) composed of phosphatidylcholine (PC), phosphatidylethanolamine (PE), and 1,2-dioleoyl-*sn*-glycero-3-[(N-(5-amino-1-carboxypentyl)iminodiacetic acid)succinyl]-nickel salt (DGS-NTA) at a molar ratio of 48/50/2 (Fig. 2 *G*–*J*). In this assay, C-terminally His_10_-tagged Cdc42 (the substrate) was anchored to Ni-conjugated SUVs, and DOCK11 was added to measure nucleotide exchange in the absence and presence of C-terminally His_10_-tagged Cdc42^Q61L^-GTP (a product analog). The SUV-based GEF activity of DOCK11 was enhanced by approximately 2.5-fold in the presence of His_10_-tagged Cdc42^Q61L^ (Fig. 2 *G* and *H*). Notably, this enhancement was not observed with nontagged Cdc42^Q61L^, which cannot bind to SUVs, indicating that association with the lipid bilayer is essential for the enhancement of DOCK11 GEF activity by Cdc42^Q61L^. Furthermore, unlike the WT DOCK11, the GEF activity of the double mutant K851A/K854S was unaffected by the addition of His_10_-tagged Cdc42^Q61L^ (Fig. 2 *I* and *J*). Collectively, these results suggest that binding of activated Cdc42^Q61L^ to the ARM domain promotes DOCK11 localization to lipid membranes and enhances GEF activity.

To further confirm the binding of activated Cdc42 to the ARM domain of DOCK11 in more complex cellular environment, we coexpressed EGFP-tagged DOCK11 (WT or double mutant) and mCherry-Cdc42^Q61L^ in human embryonic kidney (HEK)-293 T cells, and performed immunoprecipitation using cell lysates. Cdc42^Q61L^ bound less efficiently to the double mutant DOCK11 than to the WT protein (*SI Appendix*, Fig. S11). These results suggest that activated Cdc42 binds to DOCK11 in cells and that one or both of Lys851 and Lys854 play important roles in the interaction between DOCK11 and activated Cdc42.

**Fig. 3.**
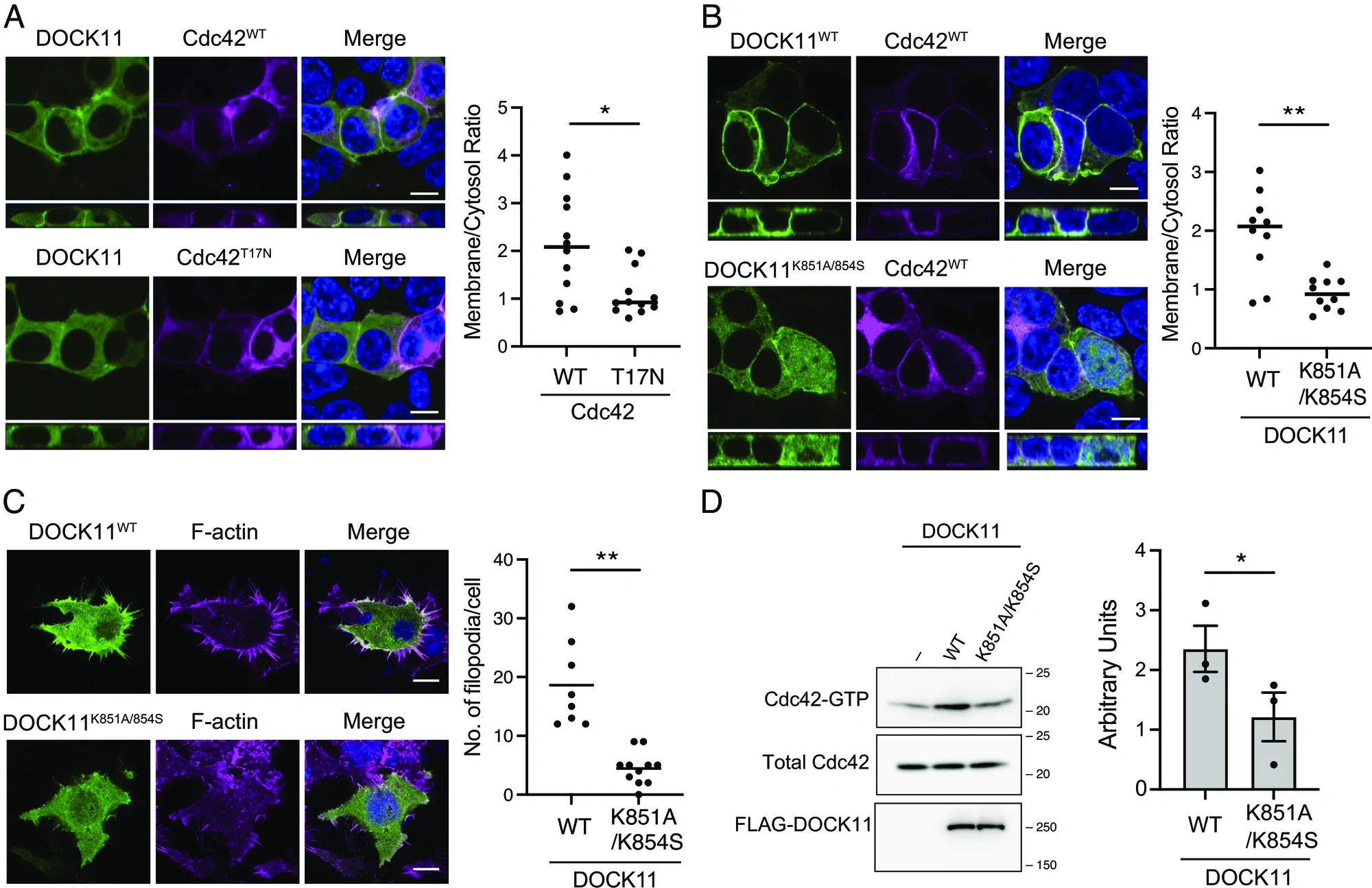
Binding of activated Cdc42 is required for cell membrane localization of DOCK11. (*A–C*) Representative confocal fluorescence images and quantitative analyses. *Lower* panels show the corresponding XZ orthogonal views. (Scale bar, 10 μm.) Graphs show representative quantification from technically independent experiments. (*A*) Effect of mCherry-tagged WT (Cdc42^WT^) or T17N mutant Cdc42 (Cdc42^T17N^) (magenta) on the localization of GFP-tagged DOCK11 (green) in HEK-293 T cells. Nuclei were stained with DAPI (blue). (*B*) Localization of WT (DOCK11^WT^) and K851A/K854S mutant DOCK11 (DOCK11^K851A/K854S^). (*C*) Filopodia formation induced by overexpression of DOCK11^WT^ or DOCK11^K851A/K854S^. (*D*) Pull-down assays measuring cellular Cdc42 activation by DOCK11^WT^ and DOCK11^K851A/K854S^. Molecular weight markers are indicated on the right. Cdc42-GTP levels were quantified by densitometry and normalized to the no-transfection control. Graphs show the mean ± SEM from three technically independent experiments. *P < 0.05, **P < 0.01 (two-tailed Welch’s *t* test for *A–C* and paired two-tailed Student’s *t* test for *D*).

Finally, we examined the effect of the binding of the activated Cdc42 to DOCK11 on the membrane localization of DOCK11 in cells using fluorescence microscopy. To examine how DOCK11 cellular localization correlates with the activation state of Cdc42, EGFP-tagged DOCK11 (WT) was coexpressed with mCherry-Cdc42 WT or the dominant negative (T17N) mutant in HEK-293 T cells. DOCK11 accumulated at the plasma membrane when expressed with the WT Cdc42, whereas its accumulation was attenuated when expressed with the T17N Cdc42 mutant (Fig. 3*A*). These results suggest that membrane localization of DOCK11 is mediated by activated Cdc42. Subsequently, to assess the binding of activated Cdc42 to the ARM domain, EGFP-tagged DOCK11 WT or the K851A/K854S double mutant were coexpressed with mCherry-Cdc42. WT DOCK11 accumulated at the plasma membrane, whereas the K851A/K854S double mutant showed a reduced accumulation (Fig. 3*B*). Consistent with these results, whereas overexpression of WT DOCK11 induced filopodia formation in HEK-293 T cells, the K851A/K854S double mutant failed to do so (Fig. 3*C*). These results indicate that the binding of activated Cdc42 to the ARM domain is crucial for the plasma membrane localization of DOCK11.

Furthermore, we examined whether the interaction with activated Cdc42 affected the GEF activity of DOCK11 in cells. When HEK-293 T cells were transfected with WT DOCK11, an increase in GTP-bound activated Cdc42 was detected in the pull-down assay (Fig. 3*D*). However, this increase in activated Cdc42 was not observed when cells were transfected with the K851A/K854S mutant. These results suggest that the association between DOCK11 and activated Cdc42 is critical for DOCK11 to catalyze the GDP–GTP exchange reaction in cells.

### The Extended Structure of DOCK11 in the Membrane-Bound State.

If the U-shaped DOCK11 dimer associates with the membrane via the DHR3-DHR1 tandem of each protomer, the DHR2 domains would be located far from the predicted membrane surface, raising the question of how DOCK11 can effectively bind to membrane-anchored Cdc42. Membrane-anchored Cdc42 likely cannot reach the active site in the DHR2 domain unless DOCK11 changes its conformation. Furthermore, the GEF activity of DOCK11 was enhanced only when SUV-bound activated Cdc42 was used in the GEF assay (Fig. 2*F*), suggesting that association with the lipid bilayer containing Cdc42 or phosphatidylinositol 3,4,5-triphosphate (PIP3) may not only promote the colocalization of DOCK11 with Cdc42 but also modulate DOCK11 activity. This prompted us to investigate the structure of DOCK11 in the membrane-bound state.

**Fig. 4.**
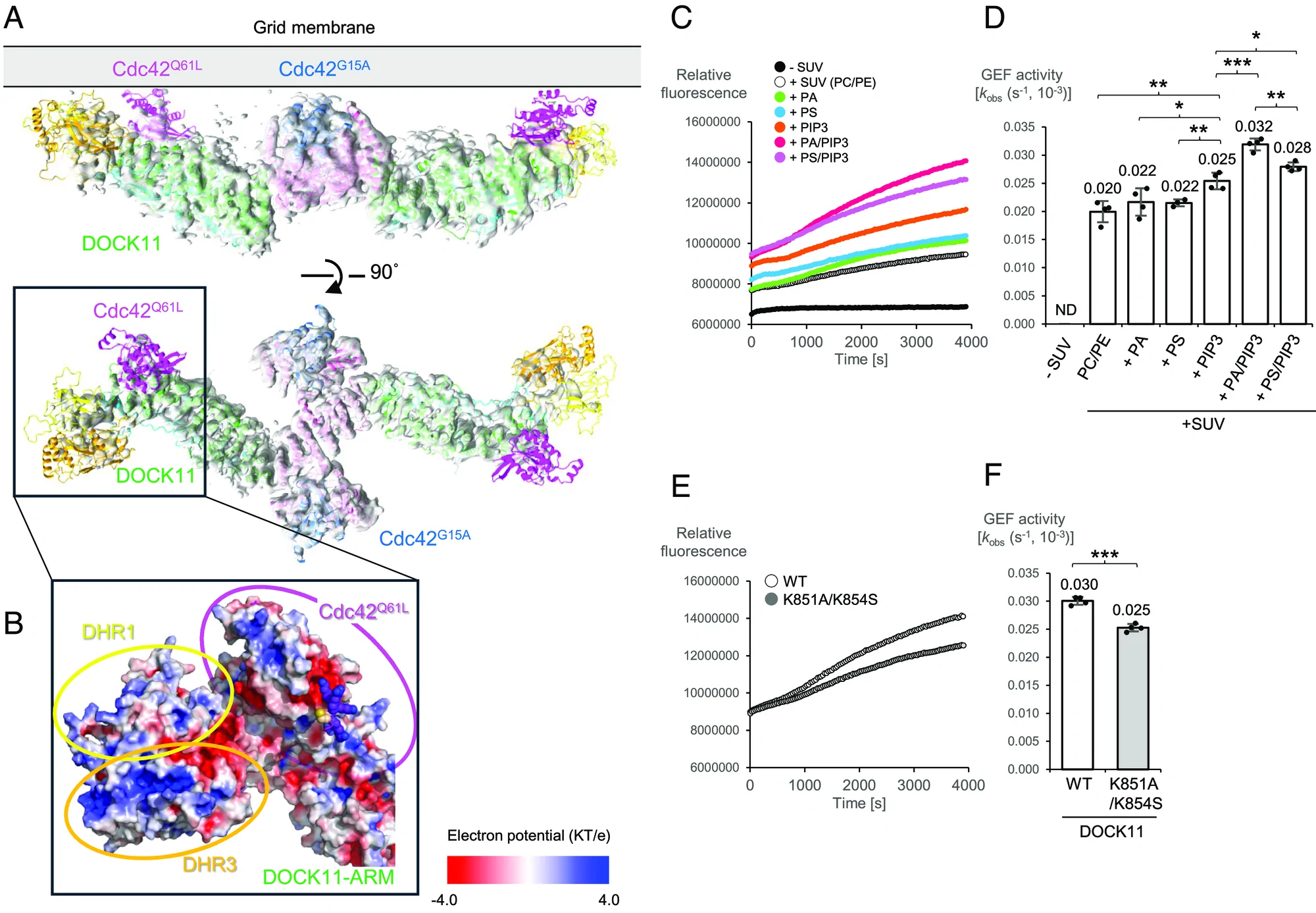
The extended structure of the DOCK11 ternary complex in the membrane-bound state. (*A*) Cryo-EM density maps and fitted structural models of the DOCK11–Cdc42^G15A^–Cdc42^Q61L^ ternary complex. The color schemes are the same as those shown in Fig. 1. Labels are shown for one protomer (Chain A) of the DOCK11 dimer and its bound Cdc42 molecules. Lowering the contour level reveals obvious densities for the DHR3-DHR1 tandem. The figures of the maps and structural models were generated using ChimeraX. (*B*) A close-up view of the DHR3-DHR1 tandem and the activated Cdc42 with a surface potential map, showing positive patches derived from lysine and arginine residues of the tandem that possibly interact with the negatively charged membrane lipids including PIP3 and PA. The figure of the surface potential map was generated using PyMOL. (*C*–*F*) GEF assays using RhoGDI–Cdc42 as the substrate to evaluate the membrane-dependent GEF activity of DOCK11 in the presence of small unilamellar vesicles (SUVs). (*C* and *D*) Effects of lipid composition on the membrane-dependent GEF activity of DOCK11. SUVs consisted of equimolar PC and PE containing 4 mol% of the indicated lipid. (*E* and *F*) Comparison of the GEF activities of WT DOCK11 and the K851A/K854S mutant in the presence of SUVs containing PIP3 and PA. (*C* and *E*) Representative fluorescence time courses. (*D* and *F*) Observed rate constants (*k*_obs_) calculated by fitting the fluorescence traces to a single-exponential rise model. Data are presented as the mean ± SD (n = 3 to 4). Statistical significance was determined using an unpaired two-tailed Student’s *t* test (*P < 0.05, **P < 0.01, ***P < 0.001).

We employed a recently developed lipid membrane-coated grid approach for cryo-EM single-particle analysis (37). The grids were prepared using a lipid mixture composed of PC, phosphatidic acid (PA), PIP3, and DGS-NTA at a molar ratio of 67/21/6/6. The concentrations of PA and PIP3 were set higher than those in the inner leaflet of the plasma membrane (38) to enhance membrane association of DOCK11 for structural characterization, based on previous reports demonstrating interactions between DOCK9 and these lipids (36, 39). Mixed protein samples of DOCK11, Cdc42^G15A^-His_10_, and Cdc42^Q61L^-His_10_ were incubated on a membrane-coated surface prior to vitrification and cryo-EM imaging. Cryo-EM data were collected at tilt angles of 0° and 30°. Interestingly, the particles predominantly adopted an elongated conformation on the membrane (*SI Appendix*, Figs. S12 and S13, see *SI Appendix*, *Materials and Methods* for details), whereas U-shaped particles, which represented the major population in the solution-based single-particle analysis, accounted for less than 10% of the membrane-bound particles (*SI Appendix*, Fig. S14). Subsequent 3D classification and reconstruction suggested that these U-shaped particles most likely correspond to molecules nonspecifically associated with the membrane rather than to a physiologically relevant membrane-bound state. A 3D reconstruction map for the extended particles was obtained without imposing symmetry (i.e., C1 symmetry) (Fig. 4*A*), reaching an overall resolution of 6.1 Å (*SI Appendix*, Table S2). The density at this resolution was sufficient to determine the relative domain organization of DOCK11. The density map revealed that Cdc42^G15A^ was bound to the DHR2 domain, whereas Cdc42^Q61L^ was bound to the N-terminal region of the ARM domain, consistent with the ternary complex model obtained under solution conditions.

Model fitting to the density map using the structure of the DOCK11 ternary complex in solution revealed that DOCK11 exhibited an extended conformation on the membrane (Fig. 4*A*). The overall structure of the DOCK11 dimer was different from that observed in solution, indicating a large conformational change upon membrane binding. Compared to the U-shaped structures of DOCK11 in solution, the N-terminal half (ranging from the N terminus to the ARM domain) was displaced as a rigid body. The structural transition from the U-shaped to the extended form is mediated by hinge motion at residue Lys1595 which is located at the boundary between the ARM (AR4) and DHR2 (Lobe A) domains (*SI Appendix*, Fig. S15). In the U-shaped conformation, the ARM–DHR2 interaction is stabilized by two C-terminal helices of the ARM domain (H2^AR4^ and H3^AR4^) and two N-terminal helices of the DHR2 domain (H1^LobeA^ and H2^LobeA^). The transition was accomplished by rotating H2^AR4^ and H3^AR4^ by approximately 60˚ in the plane parallel to that of H1^LobeA^ and H2^LobeA^, resulting in a rigid body rotation of 50˚ with a twist for the N-terminal half of Chain A extended model. The N-terminal half of Chain B is further rotated by approximately 35˚ with a twist from that of Chain A, derived from the ~30˚ rotation of H2^AR4^ and H3^AR4^ of the Chain A conformation around H1^LobeA^ (*SI Appendix*, Fig. S15).

The extended structure of the DOCK11 ternary complex shows several features that may be more favorable for membrane binding than the U-shaped structure. First, the surface of the DHR3-DHR1 tandem with Lys/Arg and PIP-binding residues lining a plane (Fig. 4*B*) faces the membrane surface with a favorable orientation which optimizes the interaction between the tandem and the membrane. Second, the DHR2 domain bound to His_10_-tagged Cdc42^G15A^ is displaced close to the membrane, indicating that membrane-anchored inactive Cdc42 can effectively bind to the catalytic site in the DHR2 domain. Third, the active Cdc42-binding site in the ARM domain is located close to the membrane and associated with the membrane-bound His_10_-tagged Cdc42^Q61L^-GTP with the same binding mode as that observed in solution, indicating that membrane-anchored Cdc42-GTP can access and bind to this site.

We further investigated the mode of interaction among DOCK11, Cdc42, and the lipid membrane using a SUV-based GEF assay. In this assay, Rho-specific guanine nucleotide dissociation inhibitor (RhoGDI)-bound lipidated Cdc42 was used as the substrate. While RhoGDI-bound Cdc42 does not efficiently interact with soluble DOCK11, nucleotide exchange occurs predominantly when DOCK11 is associated with the lipid membrane, where membrane engagement promotes dissociation of Cdc42 from RhoGDI and allows Cdc42 activation by DOCK11. Activated Cdc42 remains anchored to the membrane through its C-terminal lipid modification. Consistent with this design, little GEF activity was detected in the absence of SUVs, whereas robust activity was observed in their presence (Fig. 4 *C* and *D*).

We then examined the effects of membrane lipid composition on the membrane-dependent GEF activity of DOCK11 using PC/PE-based SUVs supplemented with 4 mol% PA, PS, or PIP3. Neither PA nor PS alone significantly affected GEF activity, whereas PIP3 markedly enhanced DOCK11 activity. Moreover, the stimulatory effect of PIP3 was further enhanced in the presence of PA (Fig. 4 *C* and *D*). These results indicate that PIP3-dependent membrane association is conserved in DOCK11 and support a role for the DHR1–PIP3 interaction in stabilizing the active conformation of DOCK11. Furthermore, the enhanced activation observed in the presence of both PIP3 and PA provides functional support for our structural model, in which the DHR3-DHR1 tandem cooperatively engages the membrane.

We also examined the GEF activity of the DOCK11(K851A/K854S) mutant under these assay conditions. The mutant exhibited significantly reduced GEF activity compared with WT DOCK11 (Fig. 4 *E* and *F*). This finding suggests that membrane-anchored activated Cdc42 further stabilizes the membrane-associated active conformation through its interaction with the active Cdc42-binding site.

Collectively, these biochemical data support the membrane-associated arrangement of the individual domains observed in the cryo-EM structure. The extended conformation enables membrane engagement by the DHR3-DHR1 tandem while simultaneously accommodating the membrane-tethered substrate Cdc42 at the DHR2 domain and membrane-anchored product Cdc42 at the ARM domain. This architecture is expected to sustain DOCK11 association with the membrane, thereby enhancing GEF activity.

Recently, we applied the same technique to determine the membrane-bound extended structure of DOCK5-ELMO1 complexed with Rac1 and RhoG (37). The differences between the solution and membrane-bound structures arose from a hinge motion of DOCK5, similar to that observed in DOCK11, and the opening angle between the ARM and DHR2 domains was nearly the same as that of membrane-bound DOCK11 (chain B) (*SI Appendix*, Fig. S16). This indicates that DOCK proteins likely adopt similar extended structures on the membrane while activating small GTPases and regulating downstream signaling.

In the membrane-bound ternary complex, weak additional density was observed between the ARM domain (AR3) of DOCK11 and the membrane surface (*SI Appendix*, Fig. S17), whereas the weak density near the C-terminal region of the ARM domain (AR4), which was assigned to the PH domain in the membrane-free reconstruction, was absent. Therefore, the extra densities are likely to correspond to the PH domain associated with the membrane via PIP3 (40, 41) present in the lipid composition. Unfortunately, the density quality was insufficient for model building. Because the PH domain is located between AWSs, with both ends connected by long flexible peptide chains, it is possible that the PH domain is relocated from the side of the ARM domain upon membrane binding.

Following the identification of the extended conformation of DOCK11, we reexamined cryo-EM datasets for apo DOCK11 and the ternary sample. Surprisingly, this revealed 2D classes with elongated shapes for a minor population of particles, resembling those observed in the membrane-coated grid data (*SI Appendix*, Fig. S18). These observations indicate that the dimeric DOCK11 molecule can also adopt an extended conformation in solution. This suggests that DOCK11 exists in an equilibrium between the predominant U-shaped and minor, extended conformations in solution.

## Discussion

Small GTPase activity is tightly regulated by GEFs, GTPase activating proteins (GAPs), and GDIs through autoinhibition and positive/negative feedback loops (42). Specifically, DOCK1, DOCK2, and DOCK5 utilize autoinhibition (43), whereas DOCK7 and DOCK11 employ positive feedback mechanisms to regulate small GTPase activity at the plasma membrane (12, 44). We determined the cryo-EM structures of DOCK11 alone, in the binary complex, and in the ternary complex in solution. Additionally, we determined the cryo-EM structure of DOCK11 in a membrane-bound ternary complex. Since the alignment of protein domains with specific functions relative to the membrane is particularly important for the regulation of signaling [3D coincidence detection (45)], our data advance the understanding of how signaling proteins, including the DOCK proteins, are associated with nanoclusters on the membrane.

ARM repeats are observed in many proteins including signaling proteins and function as platforms for protein–protein interactions (46, 47). All 11 members of the DOCK family possess ARM domains. However, the functional roles of these domains are poorly understood. In this study, we identified this ARM region as a direct binding site for the GEF reaction product, Cdc42-GTP, and demonstrate that this interaction is critical for both membrane recruitment and enhancement of GEF activity. This establishes that the DOCK ARM domain functions as a protein–protein interaction scaffold that is utilized in the positive feedback mechanism, particularly in the case of DOCK11. Notably, the ARM domain underwent no major conformational rearrangement upon binding to Cdc42-GTP, suggesting that feedback regulation was achieved primarily through recruitment and spatial organization rather than large allosteric rearrangement within the ARM domain.

Lin et al. (12) previously reported that activated Cdc42 binds to the N-terminal region (residues 66 to 126) and the DHR2 domain of DOCK11, based on immunoprecipitation assays using truncated DOCK11 fragments. In contrast, in our structure, the N-terminal region corresponding to residues 66 to 126 includes the bracing helix and is bound to the ARM domain (AR1–AR2), situated on the face opposite to the active Cdc42-binding site (*SI Appendix,* Fig. S4*B*). In addition, our complex structure suggests that activated Cdc42 is unlikely to interact directly with the DHR2 domain in the observed state. We therefore speculate that the discrepancy may arise from the use of truncated DOCK11 fragments in the previous binding assays, which could have exposed nonnative interaction surfaces or altered the conformational context of the protein.

In intracellular signaling, Rho-family GTPases activated by GEFs subsequently interact with downstream effector proteins. Several structures of activated Rho-family GTPases in complex with downstream effectors have been reported (*SI Appendix*, Fig. S19). In these structures, the Switch I and Switch II regions of the GTPases commonly form a central part of the effector-binding interface, although the effector proteins use diverse binding modes. Effectors containing a CRIB (Cdc42/Rac interactive binding) motif represent a well-characterized class of Cdc42/Rac-binding proteins and can bind Cdc42 with nanomolar affinity by extending the Cdc42 β-sheet through β2-strand interactions (48, 49). Other Cdc42 effectors lacking a CRIB motif have also been reported to bind Cdc42 with micromolar to submicromolar affinities [e.g., the formin FMNL2 (50)]. Although the DOCK11-activated Cdc42 interaction also involves the Switch regions of Cdc42, it appears to differ from previously characterized effector-binding modes because DOCK11 uses its ARM region rather than a CRIB motif, and Lys851 and/or Lys854 contribute to the high-affinity interaction. Thus, the *K*_d_ of 24 nM places DOCK11 among high-affinity activated Rho GTPase-binding proteins, and the binding mode revealed in this study distinguishes this interaction from previously reported Cdc42–effector interactions.

The DHR1 domain of DOCK11 contains a conserved PIP-binding motif similar to that found in other DOCK family members, suggesting that DHR1 mediates the interaction with PIP3-containing membranes (39, 51, 52). In contrast, the DHR3 domain lacks a canonical PIP-binding motif (53) but presents a basic patch on the surface (Fig. 4*B*), which is well suited for electrostatic interactions with negatively charged membranes enriched in anionic lipids such as PIPs and phosphatidic acid (PA). Our cryo-EM data confirmed a specific module of the two domains associated in a side-by-side manner with intensive interactions, indicating that they may work together to mediate the membrane association of DOCK11 through specific PIP-binding and nonspecific binding to acidic lipids such as PA. The DHR3-DHR1 tandem structure has also been observed in DOCK-A and DOCK-C family proteins (DOCK2, DOCK5, and DOCK6) (9, 10, 28), indicating the same functionality of the tandem. Basic residues are distributed in two distinct surface regions of the DHR3 domain across DOCK-family members, similar to those observed in DOCK11 (*SI Appendix*, Fig. S20). These conserved basic residues are positioned in locations compatible with membrane association, suggesting that they may represent a general membrane-binding feature of the DOCK-family C2 tandem structures (53). Interestingly, some signaling proteins have multiple C2 domains, and work independently to either bind to two different membranes or to lipids and proteins (54). For example, synaptotagmins have two C2 domains (C2A and C2B) that exhibit different structural features and lipid specificity (55). The two domains are separated by approximately 10 residues but are not associated with each other and work independently (56). Thus, the DHR3-DHR1 tandem of DOCK proteins, stabilized by intensive interdomain interactions, appears to be specialized for binding to membranes that are remodeled upon signal transduction (53).

The PH domain has many functions including binding to PIPs (57). Our data suggest that it is mobile and may contribute to the membrane association of DOCK11 upon its membrane recruitment. Given that the PH domain of DOCK9 shares homology with that of DOCK11 (62% identity) and exhibits specificity for PIP_2_ binding (36), the PH domain of DOCK11 is also likely to bind PIP2. Furthermore, a structural comparison of the PH domains of DOCK9 (PDB ID 1WG7) and DOCK11 with that of PLCδ1 (PDB ID 1MAI), for which the crystal structure in complex with the PIP2 head group has been reported (58), reveals residues in DOCK11 that are likely to participate in PIP2 binding (*SI Appendix*, Fig. S21).

Collectively, DOCK11 can bind to the membrane via four domains (PH and DHR1 via PIPs, DHR3 via negatively charged lipids and ARM via membrane-anchored activated Cdc42; a total of eight domains in the dimeric form), which contribute to stabilizing membrane association, facilitating binding to membrane‐anchored inactive Cdc42, and enhancing its activation. Such a multivalent binding mode may also extend the lifetime of the active state of DOCK11, which may facilitate the processive activation of Cdc42, as in the case of Ras activation by SOS (59). In the case of DOCK5 in the membrane-bound extended conformation, the DHR1 domain of DOCK5, the PH domain of ELMO1, and the membrane-anchored activated RhoG bound to the RBD of ELMO1 mediate the binding of the DOCK5–ELMO1 complex to the membrane, stabilizing the membrane-bound state of the DOCK5–ELMO1 complex in which autoinhibition is released (37). These examples indicate that membrane-mediated conformational rearrangements are critical for the functions of DOCK family proteins.

Positive feedback regulation by activated Rho-family GTPases has also been described for Dbl-family RhoGEFs, particularly members of the Lbc-family RhoGEF (60, 61). In PDZ RhoGEF, an Lbc family RhoGEF, activated RhoA binds to the PH domain and enhances further RhoA activation by increasing the effective localization of the GEF near membrane-associated RhoA substrates (61, 62). DOCK11 and Dbl-family RhoGEFs have distinct domain architectures and use structurally different modules to recognize activated GTPases. Nevertheless, in both cases, the product of the GEF reaction, an activated Rho-family GTPase, promotes membrane association or membrane retention of the upstream GEF, thereby increasing the probability of subsequent rounds of nucleotide exchange on membrane-associated substrates. Thus, the DOCK11 and Dbl families may have independently evolved a similar positive feedback strategy to support sustained and spatially restricted activation of Rho GTPases.

**Fig. 5.**
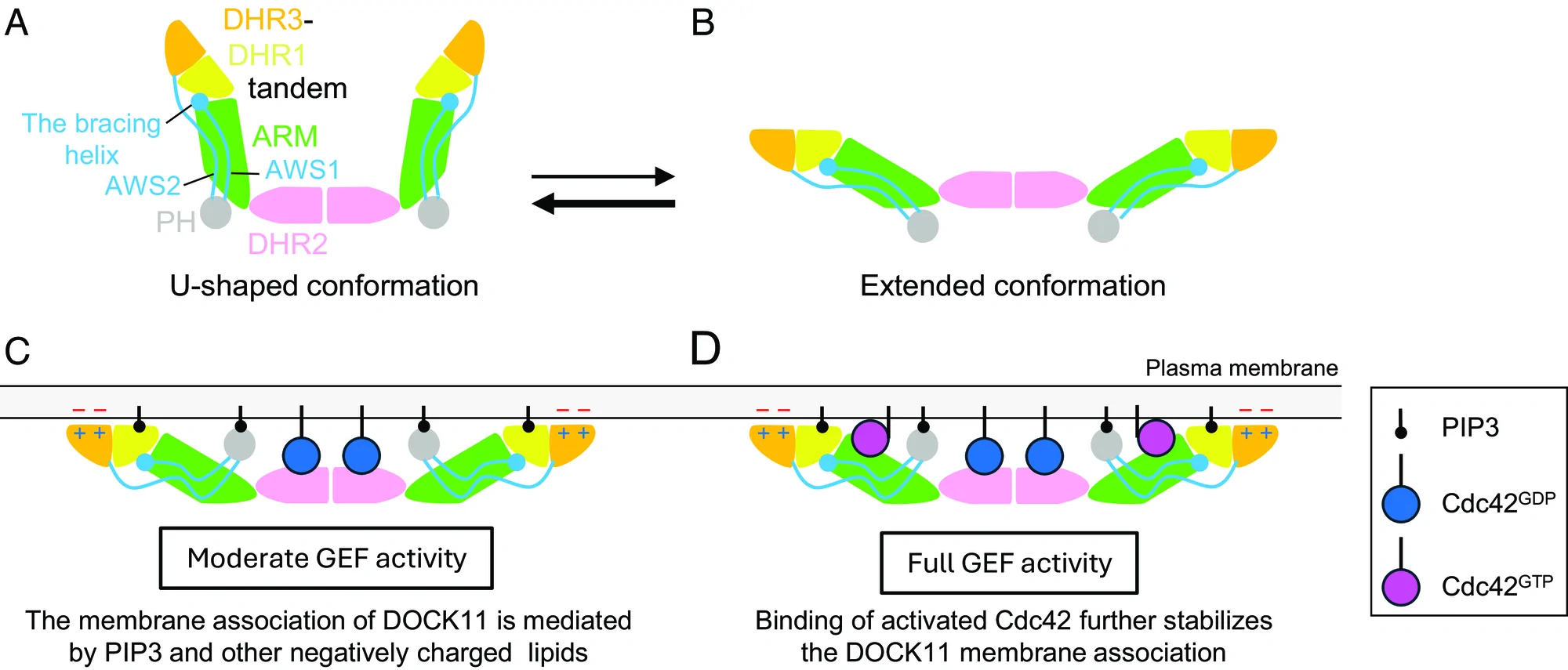
A model of membrane-dependent positive feedback regulation of DOCK11 by activated Cdc42. (*A* and *B*) DOCK11 exists in equilibrium between its U-shaped and extended conformations in the cytosol, with the former being the major conformation. DOCK11 in the U-shaped conformation may be essentially inactive because the active site in the DHR2 domain cannot access membrane-anchored Cdc42. (*C*) Upon stimulation, DOCK11 is recruited to the membrane, where it adopts an extended conformation and interacts with PIP3 and negatively charged lipids in the membrane via the DHR1 and DHR3 domains, respectively. The DHR3-DHR1 tandem is optimally oriented against the membrane and maintains the DOCK11–membrane association. The mobile PH domain may also contribute to the membrane association by binding to PIP3. Without binding to activated Cdc42, DOCK11 exhibits moderate GEF activity in vitro; however, the production of active Cdc42 is limited in vivo. (*D*) Binding of activated Cdc42 to the ARM domain further stabilizes the membrane association of DOCK11, resulting in the efficient production of activated Cdc42 with its full GEF activity.

Based on the cryo-EM structures together with biochemical and cell-based analyses, we propose a model of DOCK11 regulation through a positive feedback mechanism (Fig. 5). In the absence of stimulation, DOCK11 exists in equilibrium between its U-shaped and extended conformations in the cytosol, with the U-shaped conformation being predominant (Fig. 5 *A* and *B*). Upon stimulation, PIP3 accumulates to form nanoclusters with specific lipid molecules in the plasma membrane (63, 64), mediating the membrane recruitment of DOCK11. The lateral association of DOCK11 in the extended conformation with the plasma membrane is mediated through interactions of the DHR3-DHR1 tandem (and the PH domain) with PIP3 and other negatively charged lipids, thereby exhibiting moderate GEF activity toward Cdc42 (Fig. 5*C*). In this mode, while DOCK11 can activate Cdc42, it fails to achieve stable localization at the plasma membrane. Activated Cdc42, in turn, binds to the site within the ARM domain, thereby enhancing the membrane-binding affinity of DOCK11 and maintaining high GEF activity toward its membrane-anchored Cdc42 substrate (Fig. 5*D*). This positive feedback mechanism may enable DOCK11 to amplify Cdc42 activation localized at specific membrane regions, contributing to the spatiotemporal control of cytoskeletal dynamics and membrane remodeling. Future studies that allow real-time observation of DOCK11 dynamics within living cells are crucial to fully elucidate how this feedback mechanism operates in its native cellular context.

## Materials and Methods

Protein samples were purified according to previously established protocols using affinity and size-exclusion chromatography. Cryo-EM data were collected on a Titan Krios G4 electron microscope. The structures were validated by protein–protein interaction analyses, including immunoprecipitation, pull-down assays, and microscale thermophoresis (MST), together with cross-linking mass spectrometry, in vitro GEF assays, and cell imaging. Detailed experimental procedures are provided in *SI Appendix*.

## AI

This manuscript was prepared with the assistance of ChatGPT-5, a language model developed by OpenAI, which was used for refining language. The manuscript was further edited by Editage (www.editage.jp). The authors have reviewed, revised and approved the final content.

## Supplementary Material

Appendix 01 (PDF)

## Data Availability

EM maps are deposited in the Electron Microscopy Data Bank under the accession codes EMD-68711, EMD-68712, EMD-68713, and EMD-68714. Protein coordinates are deposited in the Protein Data Bank under the accession codes 22VD, 22VE, 22VF, and 22VG. Cross-link mass spectroscopy data have been deposited to ProteomeXchange (accession number, PXD079608) via jPOST (65) (accession number, JPST004699). Plasmids and cell lines generated and used in this study are available upon request from the authors. Source data are provided with this paper. All other data are included in the manuscript and/or *SI Appendix*.
