# Supplementary material for: Structural basis for membrane-dependent positive feedback regulation of DOCK11 by activated Cdc42
Source: Proc Natl Acad Sci U S A. 2026 Oct 6;123(41):e2607665123. doi: 10.1073/pnas.2607665123 (PMC13663769; doi:10.1073/pnas.2607665123)
Supplement: Supplementary file 1 [file pnas.2607665123.sapp.pdf]

Supporting Information for
Structural basis for membrane-dependent positive feedback regulation of DOCK11 by activated Cdc42

Tamao Hisano[1], Takehiro Shinoda[1], Mutsuko Kukimoto-Niino[1], Kazushige Katsura, Ryohei Kondo, Yoshiko Ishizuka-Katsura, Yukako Miyata-Yabuki, Kazuharu Hanada, Reiko Nakagawa, Akihiko Nishikimi, Mikako Shirouzu[2]

[1]These authors contributed equally to this work.
[2]Corresponding author: Mikako Shirouzu
Email: mikako.shirouzu@riken.jp

This PDF file includes:

SI Materials and Methods
Figures S1 to S21
Tables S1 to S3
SI References

Supporting Information Text

Materials and Methods

Protein expression and purification

G15A and Q61L mutants of Cdc42 (residues 1–188, C188S) were subcloned into the expression vector pCR2.1 (Thermo Fisher Scientific) using a modified natural polyhistidine (N11, MKDHLIHNHHKHEHAHAEH) tag, followed by a tobacco etch virus (TEV) protease cleavage site at the N-terminus. The protein was expressed using an *Escherichia coli* cell-free system (1,2) and purified by His-tag affinity chromatography, TEV digestion, removal of the N-terminal tag, and size-exclusion chromatography, as previously described (3). For purification of the constitutively active Q61L mutant, 1 mM $MgCl_2$ and 10 µM GTP were added during affinity chromatography. The final buffer contained 20 mM HEPES-NaOH (pH 7.5), 150 mM NaCl, 1 mM $MgCl_2$, and 2 mM DTT.

The gene encoding human DOCK11 (residues 1–2073) was subcloned into the mammalian expression vector pOriP (4) with N-terminal FLAG and streptavidin-binding peptide (SBP) tags followed by a TEV protease cleavage site. The resulting plasmid was designated as pOrip-FST-DOCK11. The plasmid was transfected into FreeStyle 293-F cells (Life Technologies) using 293fectin™ transfection reagent (Life Technologies) according to the manufacturer's protocol. The cells were grown in FreeStyle293 Expression medium (Life Technologies) using an incubation shaker equipped with a CO_2 controller (TAITEC) and harvested 48 h post-transfection. The cells were disrupted by sonication in buffer containing 100 mM Tris-HCl (pH 8.0), 300 mM NaCl, 100 mM arginine-HCl, 100 mM glutamic acid-Na, 500 mM sucrose, 5 mM DTT, and cOmplete protease inhibitor cocktail (Roche). Cell debris was removed by centrifugation at 10,000 ×*g* for 20 min. The supernatant was incubated with Streptavidin Sepharose resin (Cytiva) for 2 h at 4°C. The resin was washed with a buffer containing 100 mM Tris-HCl (pH 8.0), 300 mM NaCl, 100 mM arginine-HCl, 100 mM glutamic acid-Na, 500 mM sucrose, 5 mM DTT, 1 mM EDTA, and 2 mM TCEP. Proteins were eluted in the wash buffer supplemented with 4 mM desthiobiotin. TEV protease was added to the eluate and incubated overnight at 4°C. When preparing the DOCK11–$Cdc42^{G15A}$ complex, an excess of $Cdc42^{G15A}$ was added. After TEV digestion, the protein samples were subjected to size-exclusion chromatography on a HiLoad 16/600 Superose 6 pg (Cytiva) column equilibrated with a buffer containing 20 mM HEPES-NaOH (pH 8.0), 300 mM NaCl, and 1 mM TCEP. Peak fractions were pooled, concentrated, and subjected to cryo-EM analysis. Since the resolution of the map reconstructed from the uncross-linked DOCK11 sample reached only 5.6 Å (*SI Appendix*, Fig. S14), cross-linked samples were used for subsequent cryo-EM analyses to obtain higher-resolution cryo-EM data. The cross-linking reaction was performed by adding 1 mM bis(sulfosuccinimidyl)suberate (BS^3) to DOCK11 or DOCK11–$Cdc42^{G15A}$ binary complex samples, incubating at room temperature for 15 min, and quenching with 20 mM Tris-HCl (pH 8.0). The cross-linked protein was purified by a second round of SEC on a HiLoad 16/600 Superose 6 pg (Cytiva) column in 20 mM HEPES-NaOH (pH 8.0), 300 mM NaCl, and 1 mM TCEP. To prepare cryo-EM samples of the DOCK11–$Cdc42^{G15A}$–$Cdc42^{Q61L}$ ternary complex, an excess of $Cdc42^{Q61L}$ was added to the DOCK11–$Cdc42^{G15A}$ complex and incubated for 1 h at 4°C before cross-linking.

For cryo-EM analysis using membrane-coated grids and the GEF activity assay, genes encoding human Cdc42 G15A and Q61L mutants (residues 1–188, C188S) were cloned into the pCR2.1 expression vector with an N11 tag followed by a TEV protease cleavage site at the N-terminus and a decahistidine (His_{10}) tag at the C-terminus. The proteins were expressed using an *E. coli* cell-free protein synthesis system and purified by His-tag affinity chromatography as described above. After TEV protease digestion to remove the N-terminal N11 tag, the resulting C-terminal His_{10}-tagged proteins were purified using a second HisTrap affinity chromatography step followed by size-exclusion chromatography.

RhoGDI•Cdc42 complexes used for the GEF assays were prepared using a mammalian cell expression system to allow post-translational lipid modification of Cdc42. Human RhoGDI (residues 1–204) was cloned into the vector pOriP containing N-terminal FLAG and SBP tags followed by a TEV protease cleavage site. Human Cdc42 (residues 1–191) was cloned into the same vector, except that an N-terminal His_{10} tag replaced the FLAG–SBP tags, followed by a TEV protease cleavage site. RhoGDI and Cdc42 were co-expressed in 293 C18 cells (ATCC) using 293fectin and

cultured in FreeStyle 293 Expression Medium. Cells were harvested 48 h after transfection, resuspended in lysis buffer [20 mM Tris-HCl (pH 8.0), 300 mM NaCl, 2.5 mM $MgCl_2$, and DNase I], and lysed by sonication. The lysate was clarified by centrifugation at 100,000 × g for 1 h at 4°C. The RhoGDI•Cdc42 complex was purified sequentially using a HisTrap column and Streptavidin Sepharose HP resin (Cytiva). After TEV protease digestion, the complex was further purified by size-exclusion chromatography on a HiLoad 16/60 Superdex 200 pg column (Cytiva) equilibrated with 20 mM HEPES-NaOH (pH 8.0), 150 mM NaCl, 5 mM $MgCl_2$, and 2 mM DTT. The tag-cleaved complex eluted as a single monodisperse peak, and the corresponding fractions were pooled for subsequent experiments.

Cryo-EM grid preparation and data collection
Quantifoil R1.2/1.3 300 mesh copper grids were covered with a thin layer of continuous carbon film, using a JEE-420 vacuum evaporator (JEOL). The carbon-coated grids were freshly glow discharged using a PIB-10 Plasma Ion Bombarder (Vacuum Device) at 5 mA for 9 s. Protein samples were prepared at concentrations of 1.6 μM (DOCK11 apo), 0.4 μM (the DOCK11–Cdc42^{G15A} binary complex) or 0.8 μM (the DOCK11–Cdc42^{G15A}–Cdc42^{Q61L} ternary complex) in a buffer containing 20 mM HEPES-NaOH (pH 8.0), 300 mM NaCl, and 1 mM TCEP supplemented with 0.06% digitonin. Each protein (4 μL) was applied to the discharged grids in the chamber set at 4°C and 100% humidity and blotted for 1 s, followed by plunge-freezing in liquid ethane using Vitrobot Mark IV (Thermo Fisher Scientific). All datasets were collected using a Titan Krios G4 electron microscope (Thermo Fisher Scientific) operating at 300 kV and equipped with a K3 direct electron detector (Gatan) in counting mode. The movies datasets were collected at a nominal magnification of 64,000×, corresponding to a calibrated pixel size of 1.33 Å/pixel. For the DOCK11 apo dataset, each movie contained 48 frames over a 3.39 s exposure time, using a dose rate of 26.7 e^-/pixel/s, resulting in a total accumulated dose of 50.3 e^-/Å². The nominal defocus range was set between −0.8 and −2.2 μm. For the DOCK11–Cdc42^{G15A} dataset, each movie contained 48 frames over a 3.90 s exposure time, using a dose rate of 23.2 e^-/pixel/s, resulting in a total accumulated dose of 50.4 e^-/Å². The nominal defocus range was set between −0.8 and −2.0 μm. For the DOCK11–Cdc42^{G15A}–Cdc42^{Q61L} dataset, each movie contained 48 frames over a 3.35 s exposure time, using a dose rate of 26.8 e^-/pixel/s, resulting in the total accumulated dose of 50.0 e^-/Å². The nominal defocus range was set between −0.8 and −2.2 μm. The data were automatically collected using EPU software (Thermo Fisher Scientific) through the image shift method.

Membrane-coated cryo-EM grid preparation and data collection
Membrane-coated EM grids were prepared using the method developed by Shinoda et al. (5). The grids were prepared using a lipid mixture composed of egg yolk PC (Avanti, 840051), egg yolk PA (Avanti, 840101), PIP3 (Echelon, P-3916), and DGS-NTA (Avanti, 790404) at a molar ratio of 67/21/6/6. For the protein-binding step, purified DOCK11 was mixed with a two-fold molar excess of Cdc42^{G15A} and Cdc42^{Q61L} in sample buffer (20 mM HEPES-NaOH (pH 7.0), 150 mM NaCl, 1 mM $MgCl_2$) and incubated on ice for at least 1 h. A 10-μL droplet of this protein mixture (final concentration: 0.4 μM) was placed onto a PTFE plate, and the membrane-coated grid was carefully placed on top of the droplet. After a 5-min incubation, the grid was washed three times by dipping into 10-μL droplets of sample buffer. It was then blotted for 1.5 s in a fully humidified (100%) chamber at 4°C and plunge-frozen in liquid ethane using a Vitrobot Mark IV (Thermo Fisher Scientific). For the membrane-bound cross-linking experiment, the protein-binding procedure was performed as described above. After the washing steps, the grid was transferred onto a 10-μL droplet of sample buffer containing 1 mM BS^3 and incubated at 25 °C for approximately 10 min. The grid was then plunge-frozen using the same procedure described above.

Cryo-EM data for the membrane-bound DOCK11–Cdc42^{Q61L} binary complex and DOCK11–Cdc42^{G15A}–Cdc42^{Q61L} ternary complex were collected on a Titan Krios G4 electron microscope (Thermo Fisher Scientific) operated at 300 kV and equipped with a K3 direct electron detector (Gatan). Movies were recorded at a calibrated pixel size of 1.33 Å using EPU software version 3.2.0.4776 (Thermo Fisher Scientific). For the membrane-bound DOCK11–Cdc42^{Q61L} binary complex, data were collected with a total exposure of 48.8 e^-/Å² fractionated into 48 frames over 4.0 s at a dose rate of 22.0 e^-/pixel/s and a nominal defocus range of −0.8 to −2.0 μm. A total of

420 movies were collected at a 0° stage tilt. For the membrane-bound DOCK11–Cdc42^{G15A}–Cdc42^{Q61L} ternary complex, data were collected with a total exposure of 49.7 e⁻/Å² fractionated into 48 frames over 4.6 s at a dose rate of 19.4 e⁻/pixel/s and a nominal defocus range of −0.8 to −1.4 µm. A total of 1,101 movies were collected at a 0° stage tilt and 2,157 movies were collected at a stage tilt angle of 30°.

Cryo-EM data for the cross-linked membrane-bound DOCK11–Cdc42^{G15A}–Cdc42^{Q61L} ternary complex were collected on a Tecnai Arctica electron microscope (Thermo Fisher Scientific) operated at 200 kV and equipped with a K2 direct electron detector (Gatan). Movies were recorded at a calibrated pixel size of 1.47 Å. Data were collected with a total exposure of 50 e⁻/Å² fractionated into 40 frames over 12.4 s at a dose rate of 8.7 e⁻/pixel/s and a nominal defocus range of −0.8 to −1.4 µm. A total of 1,368 movies were collected at a 0° stage tilt.

Cryo-EM image processing and 3D reconstruction
Image processing was carried out in cryoSPARC v3.3 (6). Movie stacks were aligned by patch motion correction for beam-induced movement. Subsequently, the contrast transfer function (CTF) parameter of each micrograph was determined by patch CTF estimation, and the CTF effect was subsequently corrected. After manual inspection to remove poor-quality images, the remaining micrographs were frame aligned and dose weighted. Raw particle images were picked using templates generated from a preliminary 3D map. The picked raw particles were subjected to several rounds of 2D classification. After selecting good classes and removing duplicates with a minimum separation distance of 50 Å, the resulting particles were used for ab initio 3D reconstructions. After several rounds of heterogeneous refinement, the good class particles were further processed by non-uniform refinement to obtain the consensus map. Local refinement with a soft mask covering the ARM–Cdc42$^{Q61L•GTP}$ region in DOCK11 was also performed to improve the density. The consensus maps were postprocessed by DeepEMhancer (7).

Cryo-EM single-particle analysis for the membrane-coated grid data
Image processing was performed using cryoSPARC. The collected movies were motion-corrected using full-frame motion correction with dose weighting. CTF parameters were estimated from motion-corrected micrographs using Patch CTF estimation.

For the membrane-bound DOCK11–Cdc42^{Q61L} binary complex, particle picking was initially performed using Template Picker with the membrane-bound extended structure of the DOCK5–ELMO1 complex bound to Rac1 and RhoG (PDB: 9LX0) as the reference template. The extracted particles were boxed at 300 pixels, and approximately 200,000 particles were subjected to 2D classification. Classes corresponding to the extended binary complex were then used as templates for a second round of template-based particle picking. The resulting particles were extracted and subjected to ab initio reconstruction and heterogeneous refinement. Classes were selected on the basis of map quality and overall structural features. The final reconstruction was obtained by non-uniform refinement, yielding a map at 6.83 Å resolution (*SI Appendix*, Fig. S12).

For the membrane-bound DOCK11–Cdc42^{G15A}–Cdc42^{Q61L} ternary complex, particle picking was performed using Template Picker with the membrane-bound extended conformation of the DOCK11–Cdc42^{Q61L} binary complex as the reference template. Approximately 1.5 million particles from the combined 0° and 30° tilt datasets were extracted with a box size of 360 pixels and subjected to 2D classification, ab initio reconstruction (maximum resolution, 12 Å), and heterogeneous refinement. Classes exhibiting well-defined density corresponding to the extended conformation were selected and re-extracted with recentering. These particles were further processed through sequential rounds of ab initio reconstruction (maximum resolution, 10 Å), heterogeneous refinement, and ab initio reconstruction (maximum resolution, 8 Å). The final map was obtained by non-uniform refinement at a resolution of 6.07 Å (*SI Appendix*, Fig. S13). All 3D classifications were performed with C1 symmetry.

To identify particles corresponding to the U-shaped conformation, the same ternary complex dataset was reprocessed using the model of the U-shaped DOCK11 ternary complex as the

template for particle picking. Following particle extraction and 2D classification, classes corresponding to the extended conformation and evident debris were excluded, whereas all remaining classes were retained for further processing. These particles were subjected to ab initio reconstruction and heterogeneous refinement, followed by non-uniform refinement and local refinement, yielding a reconstruction at 8.1 Å resolution (*SI Appendix*, Fig. S14).

Model building, refinement, and validation

An initial model of apo DOCK11 was built by fitting the AlphaFold (8) prediction model of DOCK11 (AF-Q5JSL3-F1) into the corresponding cryo-EM map using UCSF ChimeraX (9). For the binary and ternary complexes, the crystal structures of Cdc42 (PDB IDs 2WM9, chain B; and 1NF3, chain A) were also utilized. These atomic models were manually rebuilt in Coot (10) and subjected to real-space refinement using Phenix (11). For that of the ternary complex in the extended form, the U-shaped model served as the starting point. To localize the PH domain within the extended-form map, the multi-scale cryo-EM flexible fitting protocol of GENESIS (12–14) was employed. The final models were validated using MolProbity (15). Structural figures were prepared using UCSF ChimeraX and PyMOL (16).

SUV preparation and GEF assay

The GEF activity assay in the presence of SUVs using His_{10}-tagged Cdc42 as a substrate was performed according to the method described by Medina et al. (17), with several modifications. SUVs for this GEF assay contained 48% egg yolk PC, 50% DOPE (Avanti, 850725), and 2% DGS-NTA. Lipid films were prepared by mixing lipids in chloroform solution and rapidly evaporating the chloroform under a stream of nitrogen. SUVs were prepared by resuspending the lipid film in 20 mM HEPES (pH 7.0) and 150 mM NaCl, followed by sonication using a Bioruptor (Diagenode, Belgium).

His_{10}-$Cdc42^{WT}$ was loaded with fluorescent boron-dipyrromethene-fluor (BODIPY-FL)-GDP by incubating 190 µM Cdc42 with 2-fold molar excess of BODIPY-FL-GDP in a buffer containing 20 mM HEPES (pH 8.0), 150 mM NaCl, and 10 mM EDTA at 30°C for 30 min. Nucleotide exchange was stopped by the addition of 20 mM $MgCl_2$. BODIPY-FL-GDP-loaded His_{10}-Cdc42 was purified using a gel-filtration column to remove excess nucleotides. Fluorescence assays were performed on a SPECTRAMAX iD3 multi-mode plate reader (Molecular Devices) at excitation/emission wavelengths of 485/535 nm at 30°C. In each assay, 1 µM BODIPY-FL-GDP-loaded His_{10}-Cdc42 was incubated with 100 µM GDP and SUVs (final lipid concentration, 500 µM) in reaction buffer containing 20 mM Tris-HCl (pH 7.0), 150 mM NaCl, 5 mM $MgCl_2$ and 0.2 mg/mL bovine serum albumin, with or without 250 nM His_{10}-$Cdc42^{Q61L}$-GTP. The exchange reaction was initiated by the addition of 30 nM DOCK11 or control buffer. Nucleotide exchange was measured by monitoring the decrease in BODIPY-FL-GDP fluorescence after its replacement with GDP. Fluorescence was monitored at excitation/emission wavelengths of 485/535 nm at 30°C using a SpectraMax iD3 multi-mode plate reader (Molecular Devices, San Jose, CA, USA). The observed rate constants (k_{obs}) of each reaction were determined by non-linear least-squares fitting of the data with a single exponential decay model using the KaleidaGraph software (Synergy Software, Pleasanton, CA, USA). All experiments were performed at least in triplicate.

Alternatively, a GEF assay using RhoGDI•Cdc42 complexes as substrates in the presence of SUVs was performed according to the method described by Robbe et al. (18), with modifications optimized for DOCK11. In this assay, fluorescence recovery resulting from the incorporation of BODIPY-FL-GTP into Cdc42 was monitored following the dissociation of Cdc42 from RhoGDI in the presence of both SUVs and DOCK11.

SUVs used in this assay consisted of PC and PE at equal molar ratios, with or without 4 mol% PA, PS or PIP3. PE was included because it is a major phospholipid component of the cytoplasmic leaflet and facilitates the formation of highly curved vesicles. Purified DOCK11 (final concentration, 5 nM) and SUVs (final lipid concentration, 100 µM) were mixed in assay buffer (20 mM HEPES-NaOH (pH 7.0), 150 mM NaCl, and 1 mM $MgCl_2$) and preincubated on ice for 30 min. RhoGDI•Cdc42 complexes (final concentration, 2 µM) and BODIPY-FL-GTP (final concentration,

2.4 μM) were then rapidly added, and the reaction mixture was immediately transferred to a black 96-well half-area plate. Fluorescence was monitored at excitation/emission wavelengths of 485/535 nm at 30°C for 1 h with measurements collected every 30 s using the SpectraMax iD3 multi-mode plate reader. k_{obs} were obtained by non-linear least-squares fitting of the fluorescence traces to a single-exponential rise model using the KaleidaGraph software and were used as a measure of GEF activity. All experiments were performed at least in triplicate.

Cross-linking mass spectrometry

The BS^3 cross-linked DOCK11–Cdc42^{G15A}–Cdc42^{Q61L} ternary complex was analyzed by cross-linking mass spectrometry. Proteins were reduced with tris(2-carboxyethyl)phosphine (TCEP) and alkylated with iodoacetamide (IAA), followed by digestion with Trypsin/Lys-C. The resulting peptides were fractionated using SDB–SCX StageTips (GL Sciences) according to the manufacturer's instructions. Peptides were sequentially eluted into five fractions using 20% acetonitrile (ACN) and 0.5% acetic acid (AcOH) containing increasing concentrations of ammonium acetate (NH_4OAc) (20, 50, 200, 500, and 1,000 mM). The eluates were dried in a SpeedVac concentrator, reconstituted in 0.1% trifluoroacetic acid (TFA) and 2% ACN, and subjected to LC–MS/MS analysis.

Peptides were analyzed using an Orbitrap Eclipse mass spectrometer coupled to a Vanquish Neo UHPLC system. Peptides were loaded onto a C18 trap column (PepMap Neo Trap Cartridge, 0.3 × 5 mm, 5 μm particle size; Thermo Fisher Scientific) and separated on an Aurora C18 column (75 μm × 250 mm, 1.7 μm particle size; IonOpticks) at a flow rate of 300 nL/min. Solvent A consisted of 0.1% formic acid in water, and solvent B consisted of 0.1% formic acid in acetonitrile. The 75-min gradient was as follows: 0–1 min, 0%–2% B; 1–2 min, 2%–5% B; 2–66 min, 5%–45% B; 66–70 min, 45%–95% B; and 70–75 min, 95% B. MS/MS data were acquired in positive ion mode. Full MS spectra were acquired in the Orbitrap at a resolution of 60,000 over an m/z range of 380–1400. Precursor ions with charge states of 3–8 were selected for HCD fragmentation using an isolation window of 1.6 m/z and stepped normalized collision energies of 21%, 26%, and 31%. MS/MS spectra were acquired in the Orbitrap at a resolution of 30,000. Dynamic exclusion was set to 60 s.

Raw data were analyzed using Proteome Discoverer 3.3 SP1 (Thermo Fisher Scientific) with the XlinkX node. MS/MS spectra were searched against a custom database containing the amino acid sequences of DOCK11 and Cdc42 mutants (G15A or Q61L). Trypsin/P was specified as the digestion enzyme with a maximum of three missed cleavages and a minimum peptide length of five amino acids. DSS cross-links on lysine residues (+138.068 Da) were specified as the cross-linking modification. The precursor and fragment mass tolerances were set to 10 ppm and 20 ppm, respectively. Carbamidomethylation of cysteine residues was set as a fixed modification, while methionine oxidation and protein N-terminal acetylation were set as variable modifications. Results were filtered to a 1% false discovery rate (FDR).

Protein-protein interaction analysis using MST

For binding assays, C-terminally truncated DOCK11 (residues 1–1595, wild-type and K851A/K854S mutant) with N-terminal FLAG and SBP tags were subcloned into the pcDNA3.4 vector (Thermo Fisher Scientific) and expressed and purified using the SBP affinity tag in the same manner as the full-length protein described above. Purified DOCK11 protein was prepared in serial dilutions (0.03–500 nM) in a buffer containing 20mM HEPES (pH 8.0), 150 mM NaCl, 1mM $MgCl_2$, 0.5mM TCEP, and 0.05% (v/v) Tween-20. His_{10}-tagged Cdc42 (wild type and Q61L mutant) was fluorescently labeled using the Monolith His-Tag Labeling Kit, RED-tris-NTA 2 Generation (NanoTemper). MST was measured at 25°C with a Monolith NT.115 (NanoTemper), according to the manufacturer's protocol. Each experiment was repeated three times and dissociation constants were calculated using MO.Affinity Analysis Software (Nanotemper). The fitting error represents the 68% confidence interval of the fit.

Cell imaging analysis by fluorescence microscopy

Constructs
To express N-terminally tagged EGFP or mCherry fusion proteins in mammalian cells, we inserted cDNA encoding EGFP or mCherry into pCI (Promega) to create pCI-EGFP and pCI-mCherry vectors, respectively. cDNAs encoding human DOCK11 (wild-type and K851A/K854S mutant) were amplified by PCR using pOrip-FST-DOCK11 and pOrip-FST-DOCK11 K851A/K854S, respectively, and subcloned into pCI-EGFP. The cDNA encoding human Cdc42 was amplified by PCR using cDNA prepared from HEK-293T cells and subcloned into pCI-mCherry. The pCI-mCherry-$Cdc42^{Q61L}$ mutant was generated using inverse PCR.

Immunofluorescence
Expression vectors were transfected into HEK-293T cells with PEI Max (Polysciences) according to the manufacturer's instructions and incubated for 24 h. Cells were fixed with 4% paraformaldehyde for 10 min and stained with DAPI (1 µg/mL) for 30 min at room temperature. To visualize filamentous actin, we permeabilized the fixed cells with 0.2% Triton X-100 in PBS for 10 min and then stained with Alexa555-conjugated phalloidin (Thermo Scientific). Fluorescence images were acquired using a STERALIS 5 laser confocal microscope (Leica Microsystems). The acquisition of z-stack images and construction of 3D images were performed using the Leica Application Suite X software. The average membrane-to-cytosol fluorescence ratio for each cell was determined by measuring fluorescence intensities at three randomly selected locations in the membrane and cytosol using Fiji software.

Immunoprecipitation and pull-down assay
The interaction between DOCK11 and Cdc42 was analyzed by immunoprecipitation. HEK-293T cells expressing EGFP-DOCK11 (wild type or the K851A/K854S mutant) and mCherry-$Cdc42^{Q61L}$ were lysed in lysis buffer (25 mM HEPES, 150 mM NaCl, 10 mM $MgCl_2$, 1 mM EDTA, 2% glycerol, 1% NP-40), and insoluble debris was removed by centrifugation. Supernatant was incubated with ChromoTek RFP-Trap Magnetic Agarose (Proteintech) and washed with the lysis buffer. The precipitated mCherry-Cdc42 (Q61L) and its associated molecules were subjected to SDS-PAGE and immunoblotting using anti-GFP or anti-RFP tag antibodies (Proteintech). The blot was developed with Western BLoT Quant HRP Substrate (Takara Bio). Chemiluminescence was detected and quantified using an Amersham Imager 600 (GE Healthcare).

Active Cdc42 in the cell was detected using an Active Cdc42 pull-down and detection kit (Thermo Fisher Scientific) according to the manufacturer’s instructions. Briefly, the cell extracts were incubated with the Pak1 PBD domain-conjugated beads for 30 min at 4°C. The bound proteins and total lysates were subjected to SDS-PAGE and immunoblotting with an anti-Cdc42 antibody or anti-FLAG-tagged antibody (Wako Pure Chemicals) as described above.

Figures

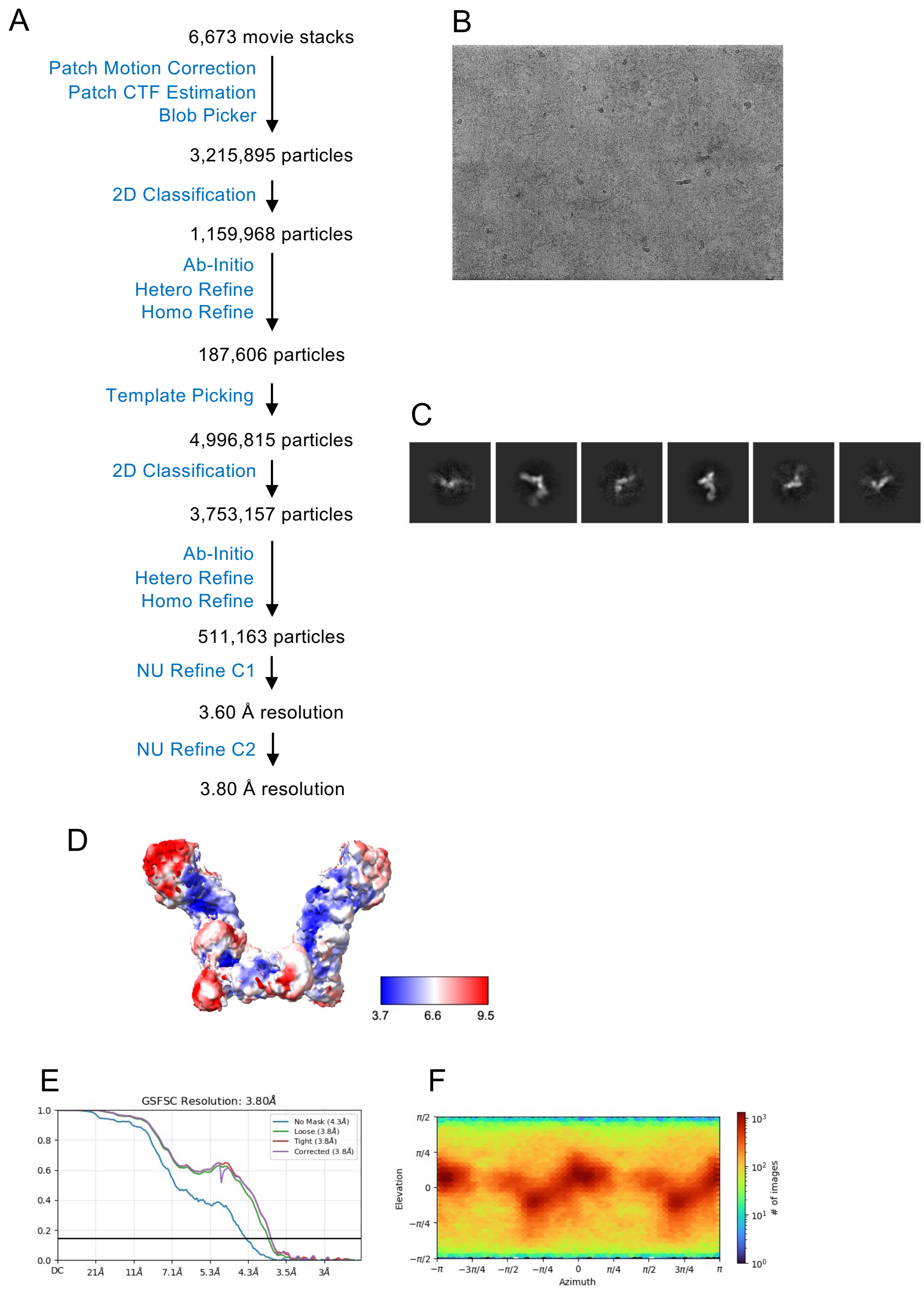

Fig. S1. Flowchart of cryo-EM data processing for the DOCK11 apo dimer. (*A*) Processing workflow for the DOCK11 apo data. (*B*) A representative motion-corrected cryo-EM micrograph of DOCK11. (*C*) 2D class averages of DOCK11 used to build an *ab initio* model. (*D*) A local resolution map. (*E*) FSC curve of the final cryo-EM map. (*F*) Particle orientation distribution from the final NU refinement.

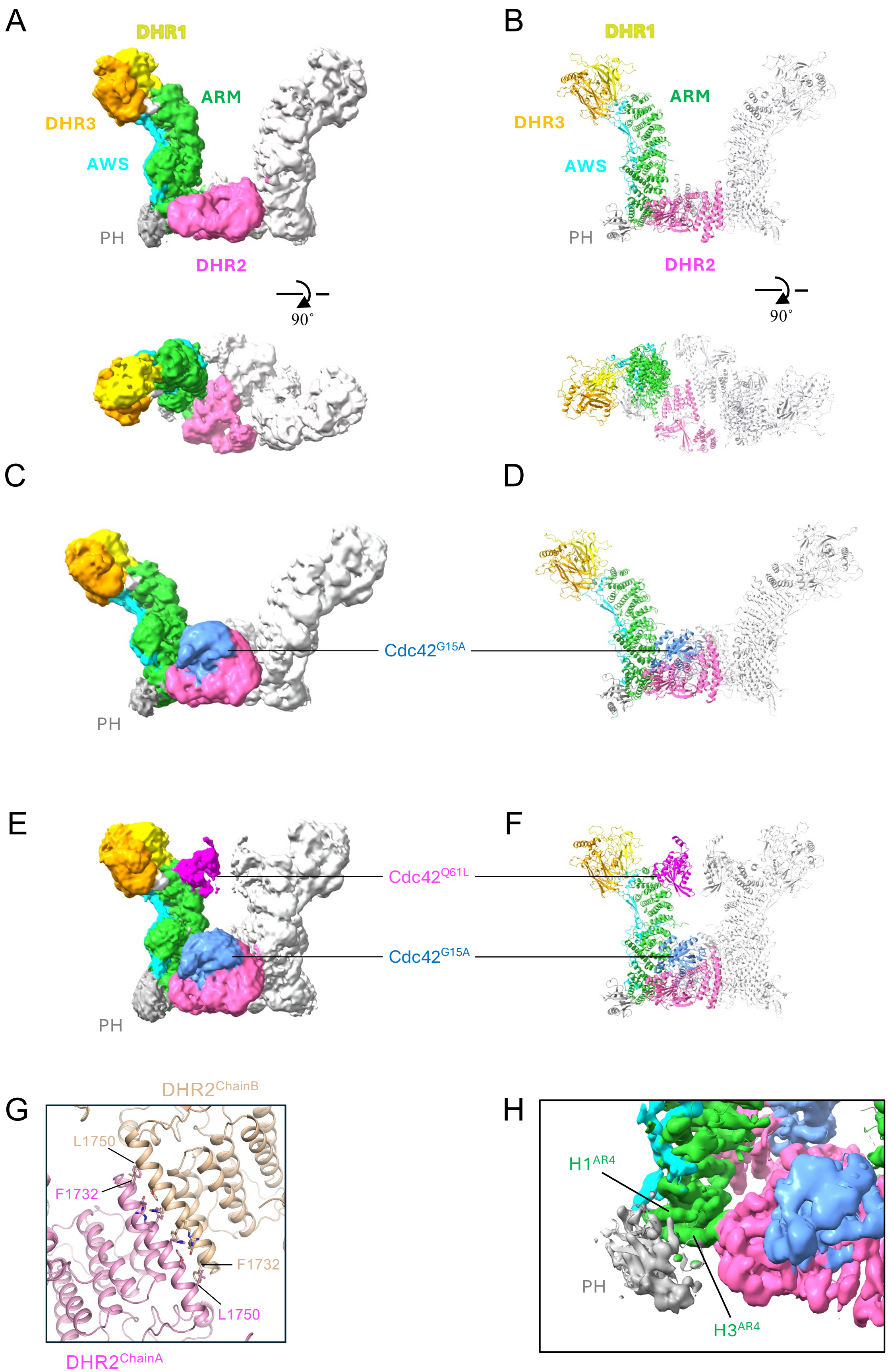

A
DHR1
ARM
DHR3
AWS
PH
DHR2
90˚
B
DHR1
ARM
DHR3
AWS
PH
DHR2
90˚
C
D
Cdc42G15A
PH
E
F
Cdc42Q61L
Cdc42G15A
PH
G
DHR2ChainB
L1750
F1732
F1732
L1750
DHR2ChainA
H
H1AR4
PH
H3AR4

Fig. S2. Cryo-EM density maps and dimeric models of DOCK11 in solution, showing the densities and positions of the PH domain and Cdc42 bound to the ARM domain. (*A-F*) Cryo-EM density maps and dimeric models of (*A,B*) DOCK11, (*C,D*) DOCK11–Cdc42^{G15A} binary complex, and (*E,F*) DOCK11–Cdc42^{G15A}–Cdc42^{Q61L} ternary complex, showing the densities and location of the PH domain and Cdc42^{Q61L} bound to the ARM domain. (*G*) The DHR2 dimeric interaction. Residues at the dimer interface (F1732, T1736, Y1739, R1740, H1743, Y1746, T1747, and L1750) are shown as stick models. (*H*) A close view of the density map showing densities corresponding to the PH domain (grey) observed in the ternary complex dataset. H1^{AR4} and H3^{AR4} are the C-terminal helices of the ARM domain (the first and third helices of AR4, see *SI Appendix,* Fig. 4*A*).

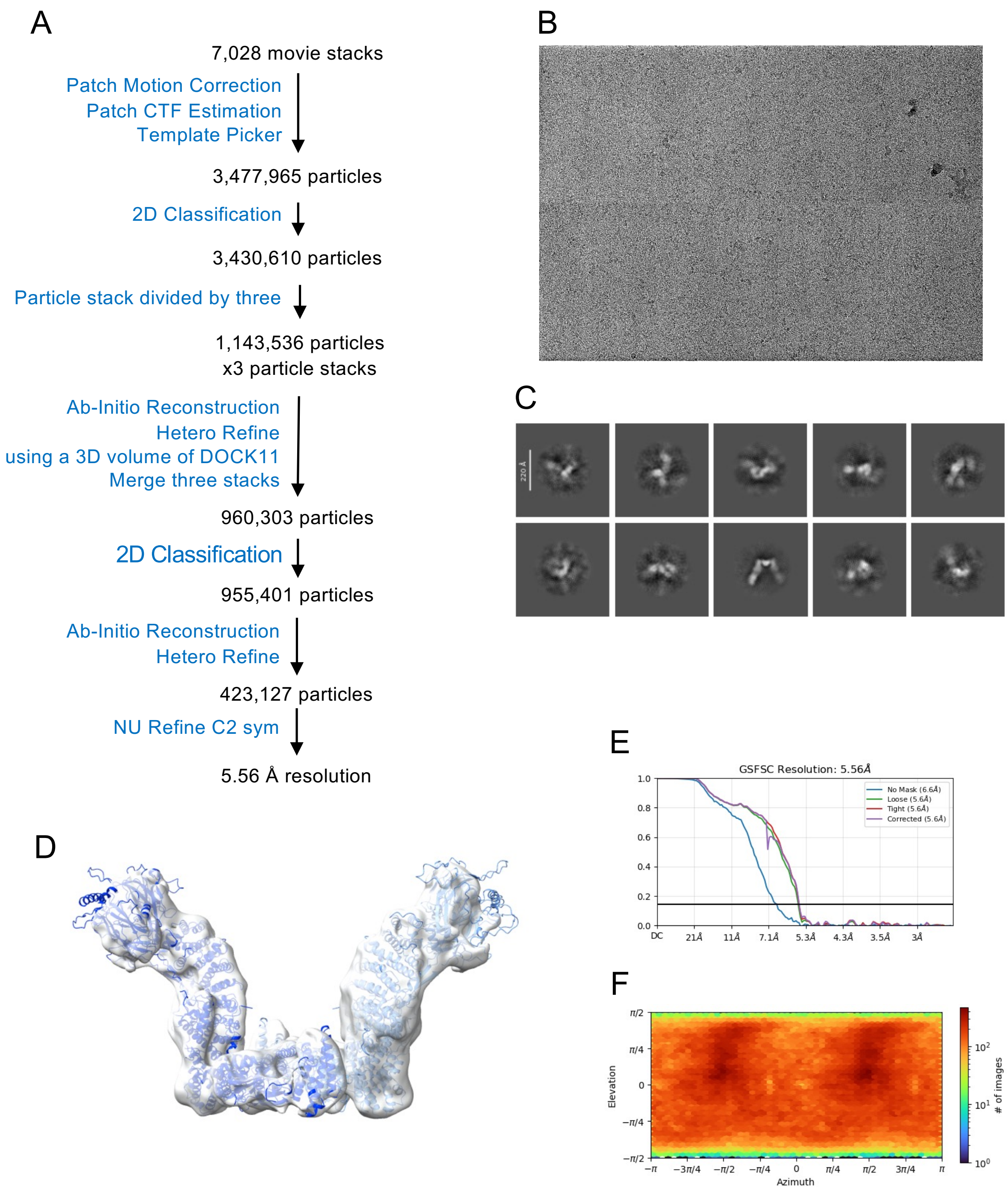

Fig. S3. Cryo-EM analysis of the DOCK11 apo dimer without cross-linking. (*A*) Processing workflow. (*B*) A representative of motion corrected cryo-EM micrographs. (*C*) 2D class averages used to build an *ab initio* model. (*D*) 3D reconstructed map. (*E*) FSC curve of the final cryo-EM map. (*F*) Particle orientation distribution from the final NU refinement.

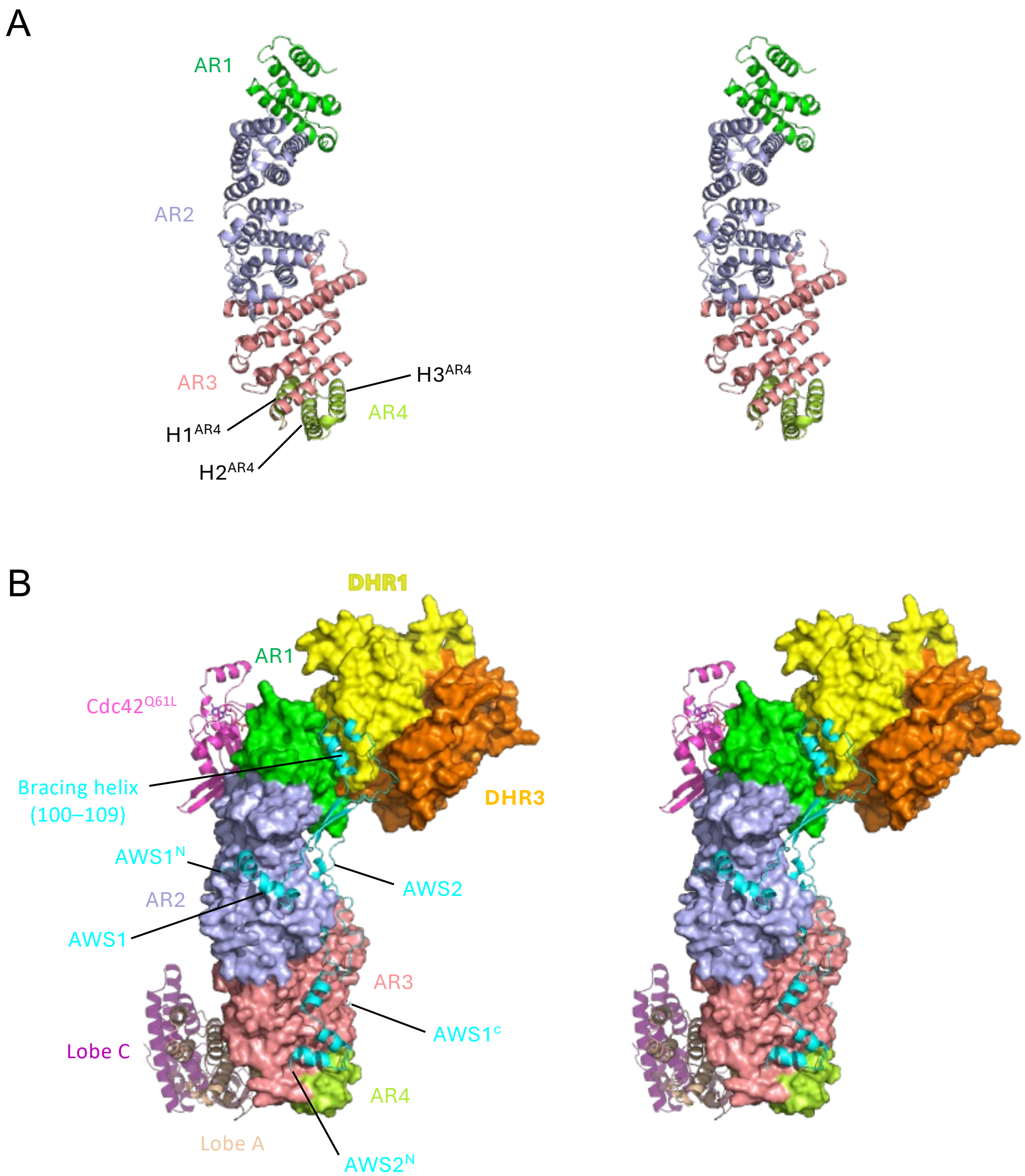

Fig. S4. The ARM domain (stereo view). (*A*) The ARM domain is divided into four regions (AR1–AR4) based on the orientations of helices. AR1: residues 824–930 (green); AR2: residues 931–1305 (sky blue); AR3: residues 1306–1519 (salmon); AR4: residues 1520 –1595 (wheat). (*B*) The ARM domain, as well as the DHR3–DHR1 tandem, is shown as a surface model, with the same orientation as *A*. AWS1 (residues 40–151; cyan) initiates at the center of the convex surface of the ARM domain (indicated by AWS1^{N}) and runs upward toward the DHR1–ARM interface, where it forms the bracing helix (100–109) which mediates the interaction between the DHR1 and ARM domains. It then traverses the concave surface of the ARM domain, running downward along the longitudinal axis from AR1 to AR3 (indicated by AWS1^{C}). Following the PH domain (not shown), AWS2 (residue 310–363; cyan) runs upward from the bottom of AR3 (indicated by AWS2^{N}) along the ARM domain, connecting to the DHR3 domain. The color scheme is the same as in *A* for AR1–AR4, with DHR1 colored in yellow and DHR3 in orange. Lobe A and Lobe C of the DHR2 domains are also shown in wheat and violet, respectively. Cdc42^{Q61L} (magenta) is also shown to indicate its binding site in the ARM domain.

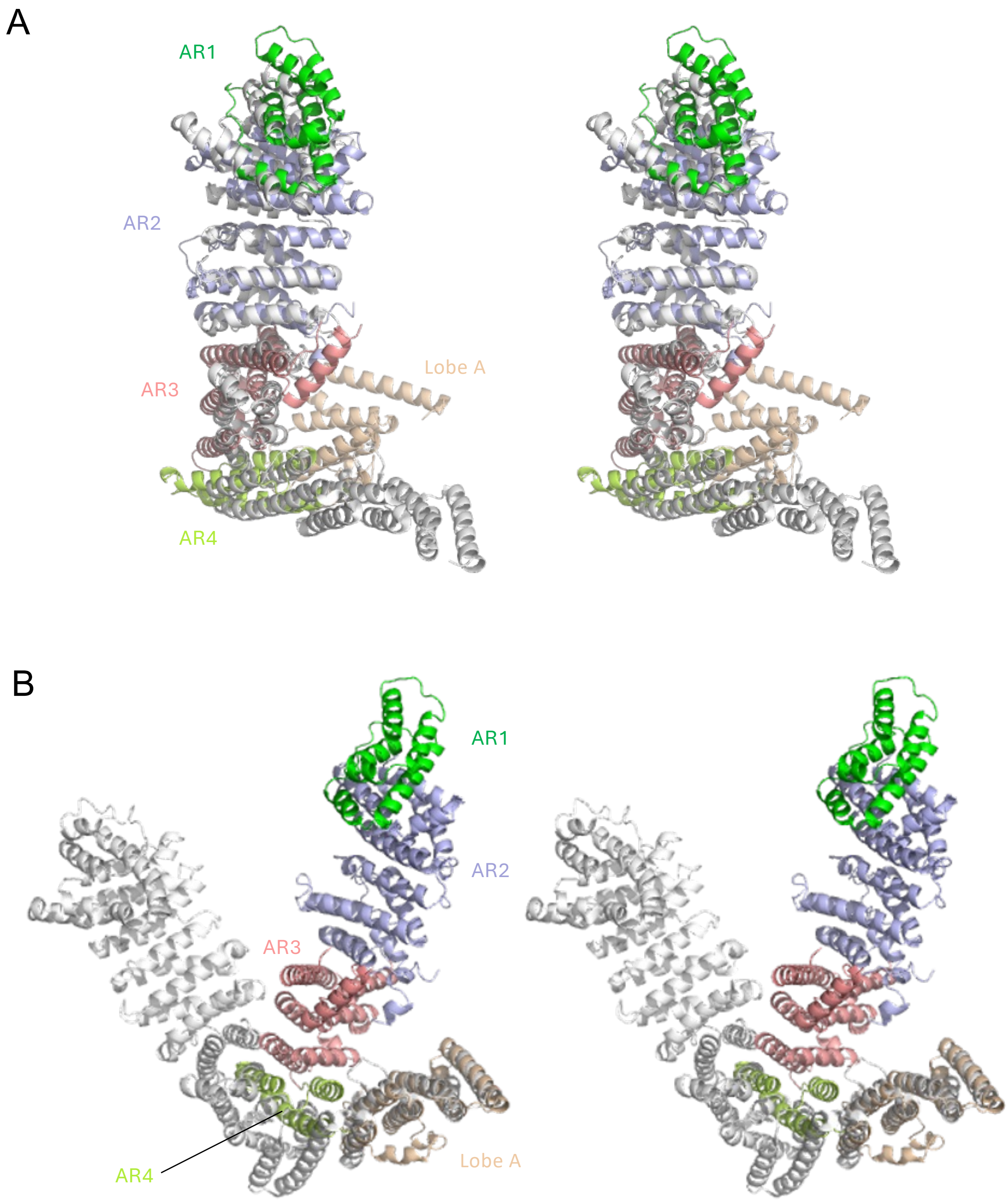

Fig. S5. Conformational differences in the ARM-DHR2 domains between DOCK11 (in solution) and DOCK5 (8ZJI) (stereo view). The conformations of the ARM and DHR2 (Lobe A) domains of DOCK11 (various colors) and DOCK5 (gray) are compared by superposing (*A*) the ARM domains and (*B*) the DHR2 domains (Lobe A).

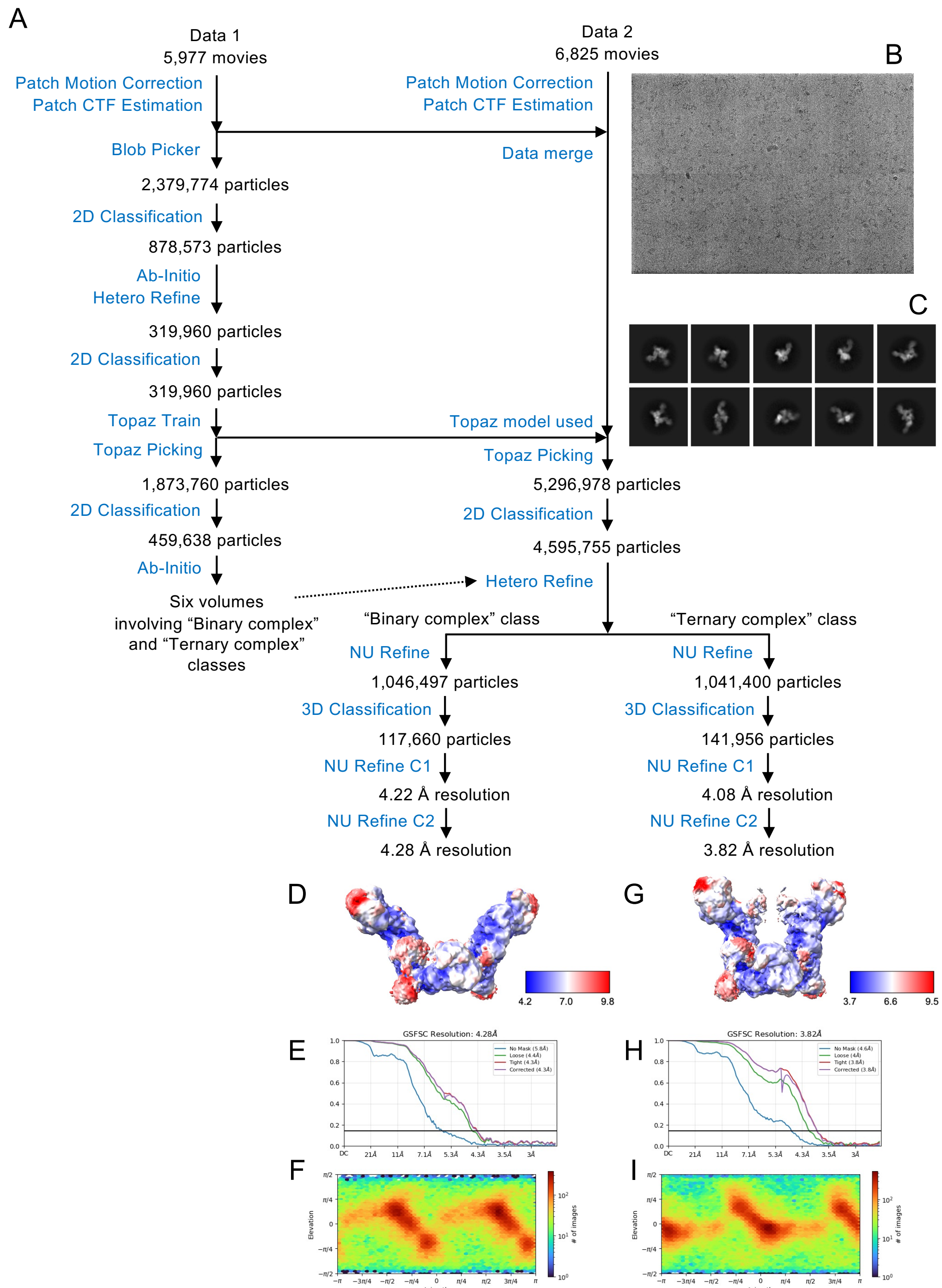

Fig. S6. Flowchart of cryo-EM data processing for the "ternary sample". (*A*) Processing workflow for the "ternary sample" data. (*B*) A representative of motion corrected cryo-EM micrographs. (*C*) 2D class averages of the "ternary sample" data used to build an *ab initio* model. (*D*) A local resolution map, (*E*) FSC curve of the final cryo-EM map, and (*F*) particle orientation distribution from the final NU refinement for the "binary complex" map. (*G*) Local resolution map, (*H*) FSC curve of the final cryo-EM map, and (*I*) particle orientation distribution from the final NU refinement for the "ternary complex" map.

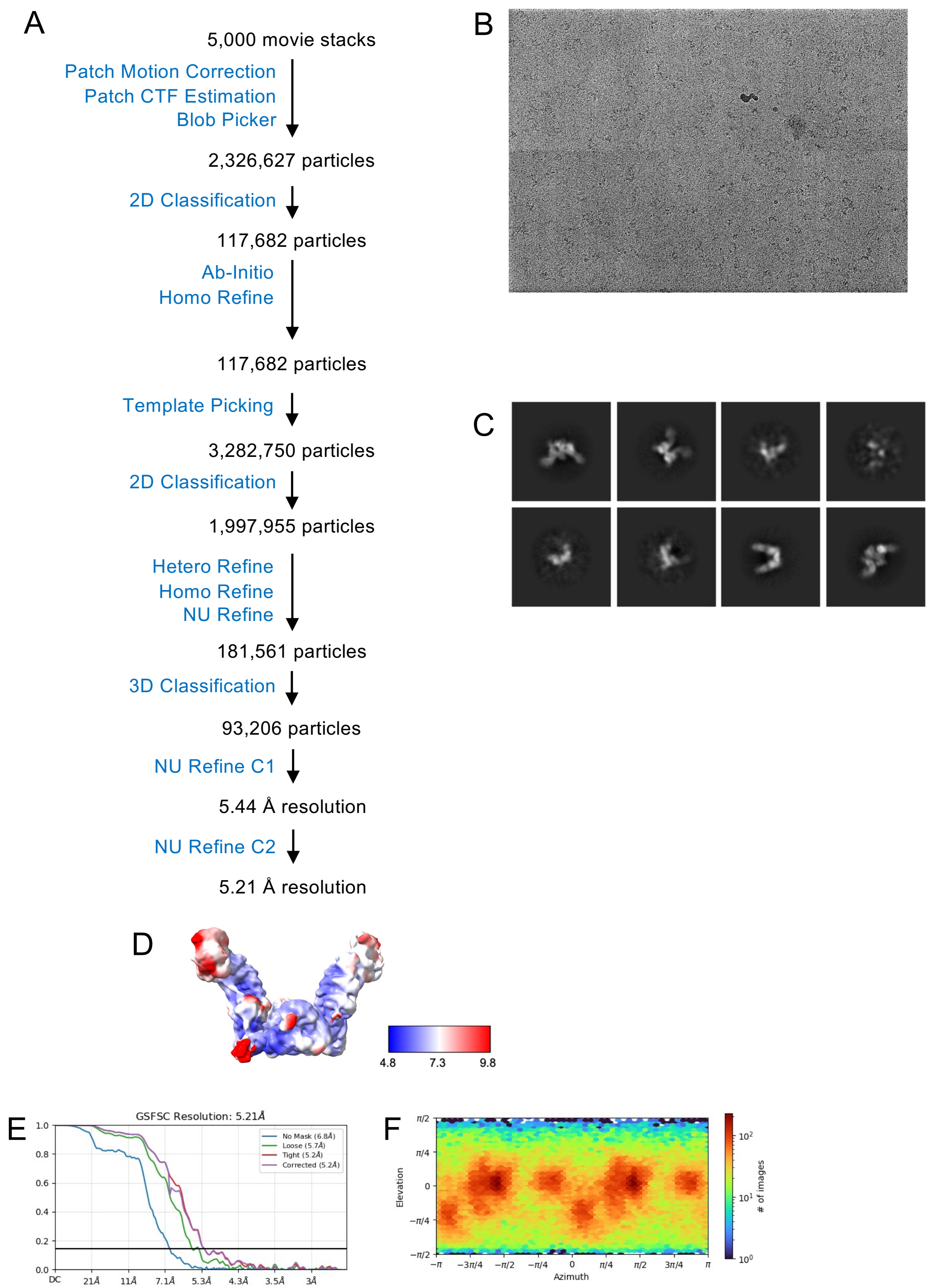

Fig. S7. Flowchart of cryo-EM data processing for the "binary sample". (*A*) Processing workflow for the "binary sample" data. (*B*) A representative motion-corrected cryo-EM micrograph of the binary complex. (*C*) 2D class averages of the binary complex used to generate an *ab initio* model. (*D*) Local resolution map. (*E*) FSC curve of the final cryo-EM map. (*F*) Particle orientation distribution from the final NU refinement.

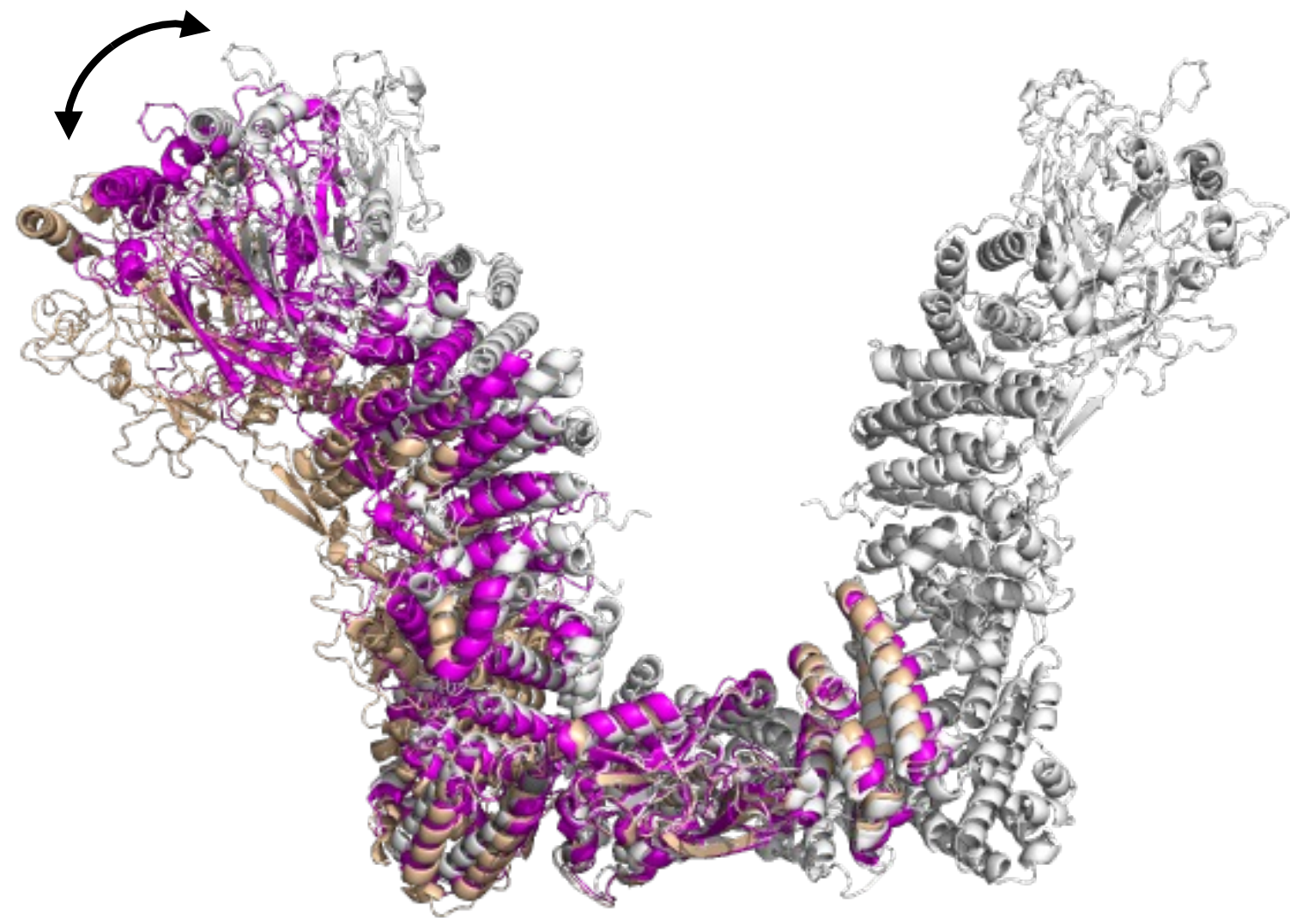

Fig. S8. Conformational flexibility of DOCK11. Structures of DOCK11 protomers (Chain A) of the apo dimer (magenta), the binary complex (brown), and the ternary complex (white) are superimposed based on the DHR2 domain, showing pivot motions at the ARM–DHR2 domain connection. The N-terminal half (the whole region excluding the DHR2 domain) behaves as a rigid body. The other protomer (Chain B; white) of DOCK11 in the ternary complex is also shown.

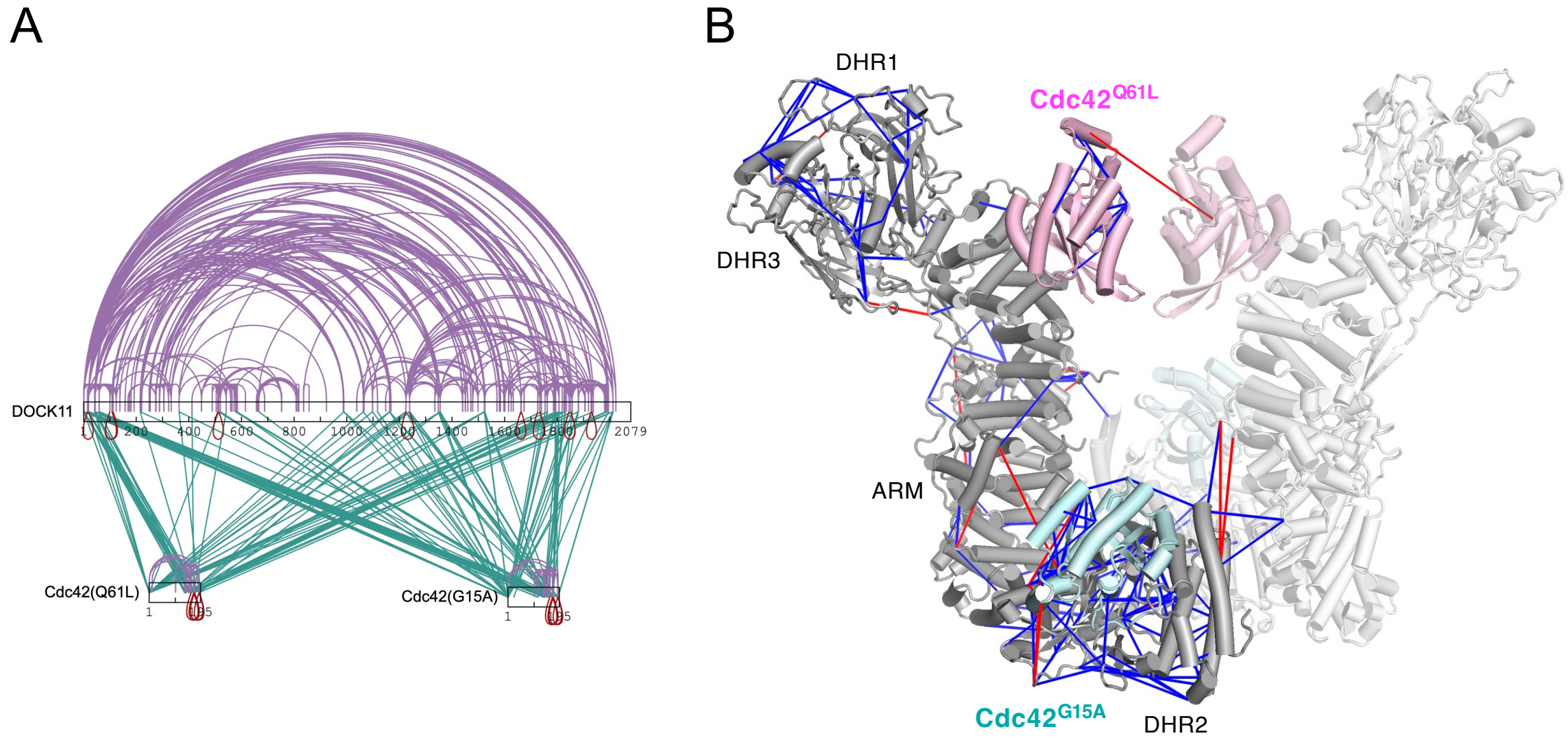

Fig. S9. Cross-linking mass spectrometry of the DOCK11–Cdc42^{G15A}–Cdc42^{Q61L} ternary complex. (*A*) Intermolecular and intramolecular BS^3 cross-links detected across DOCK11 and Cdc42 (G15A or Q61L) in three technically replicated experiments. The residue numbers indicated by bars include the N-terminal linker sequence (DOCK11: GGSGGS, Cdc42: GSSGSSG). (*B*) Validated cross-links mapped onto the ternary complex structure. Blue lines indicate cross-links with distances of less than 30 Å between lysine Cα atoms, which are acceptable with the BS^3 cross-linker, while red lines indicate cross-links with distances in the 30–50 Å range, suggesting structural flexibility.

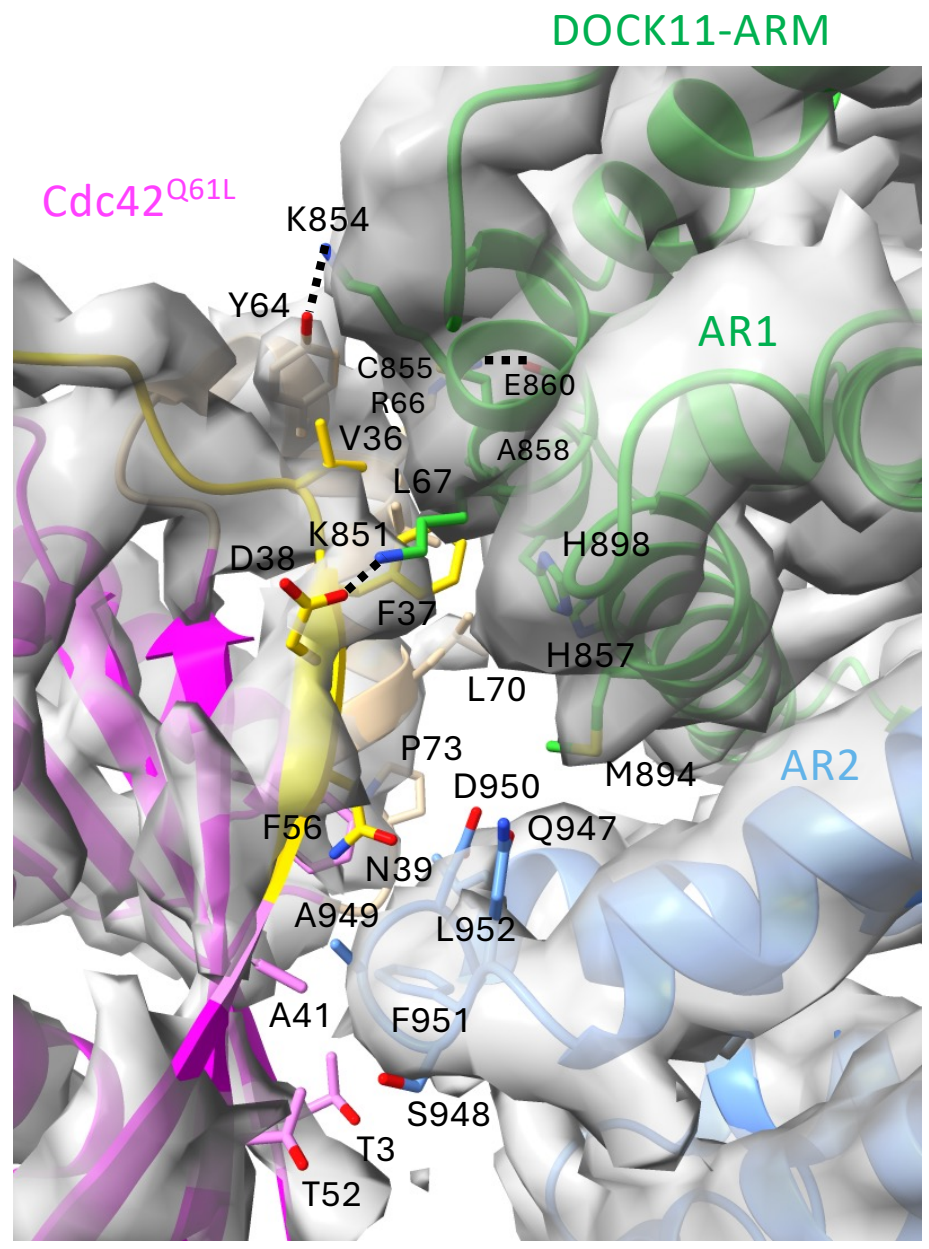

Fig. S10. Local refinement density map of the ARM domain (AR1 and AR2) and bound Cdc42^{Q61L}, viewed from the same direction as Fig. 2*A*. Residues potentially involved in the interaction are shown as sticks. The color scheme is the same as used in Fig. 2*A*.

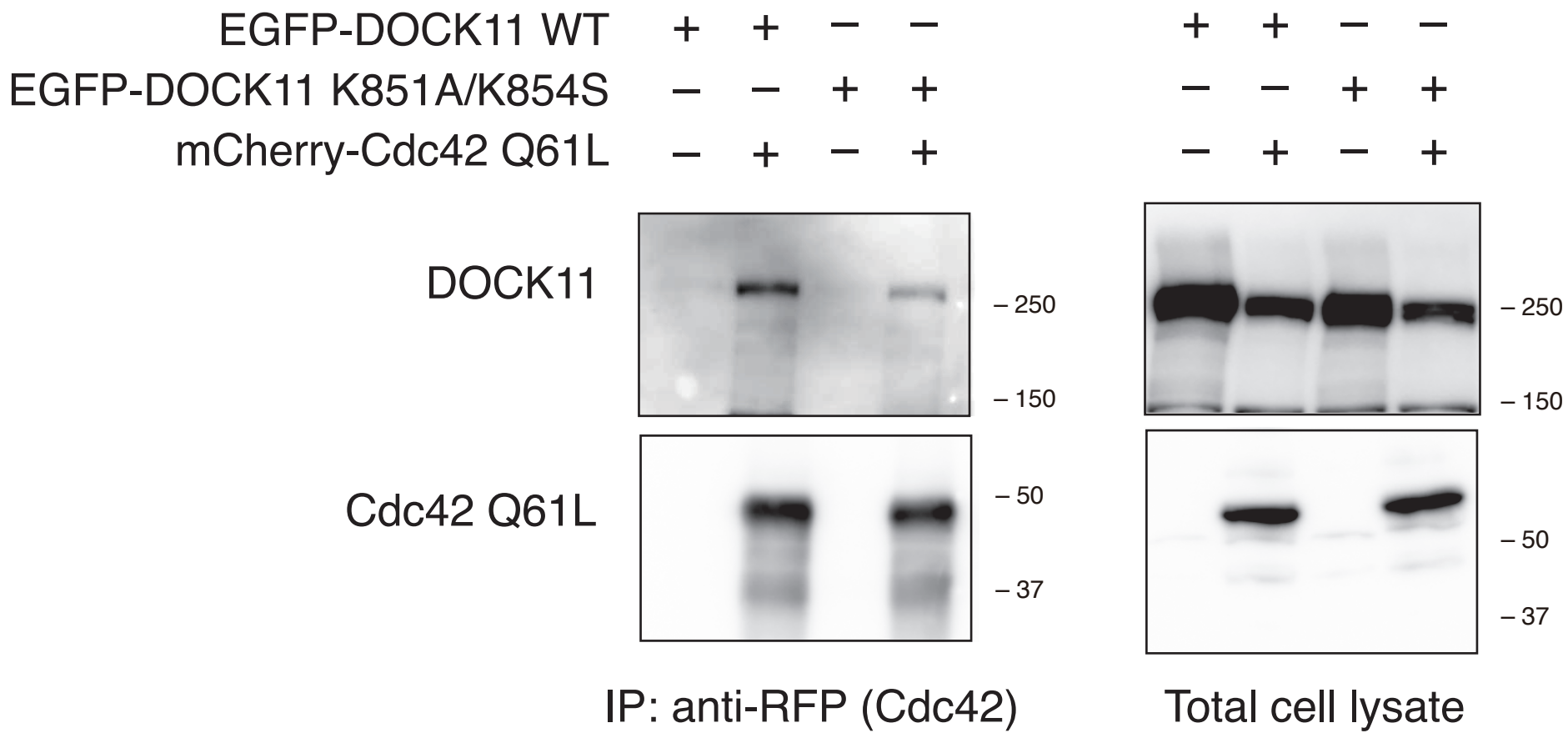

Fig. S11. Activated Cdc42 binds to the ARM domain of DOCK11 in HEK-293T cells. Following expression of EGFP-tagged DOCK11 (wild type or double mutant) with mCherry-tagged Cdc42^{Q61L} in HEK-293T cells, cell lysates were subjected to immunoprecipitation using RFP-Trap magnetic agarose, followed by immunoblotting. Molecular weight markers (kDa) are indicated on the right. A representative result from four technically independent experiments is shown.

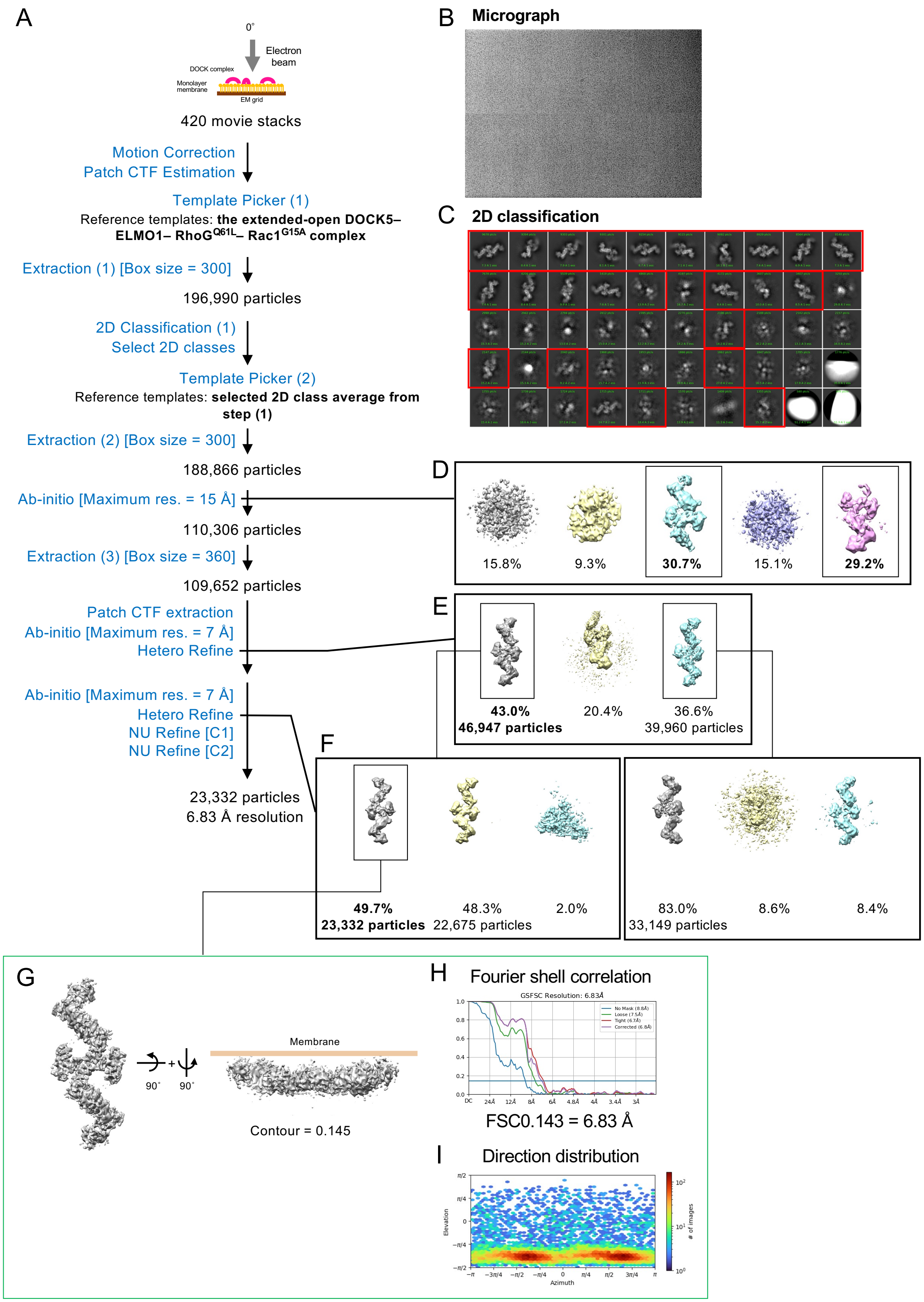
A
0°
Electron beam
DOCK complex
Monolayer membrane
EM grid
420 movie stacks
Motion Correction
Patch CTF Estimation
Template Picker (1)
Reference templates: the extended-open DOCK5–ELMO1– RhoG^{Q61L}– Rac1^{G15A} complex
Extraction (1) [Box size = 300]
196,990 particles
2D Classification (1)
Select 2D classes
Template Picker (2)
Reference templates: selected 2D class average from step (1)
Extraction (2) [Box size = 300]
188,866 particles
Ab-initio [Maximum res. = 15 Å]
110,306 particles
Extraction (3) [Box size = 360]
109,652 particles
Patch CTF extraction
Ab-initio [Maximum res. = 7 Å]
Hetero Refine
Ab-initio [Maximum res. = 7 Å]
Hetero Refine
NU Refine [C1]
NU Refine [C2]
23,332 particles
6.83 Å resolution
B Micrograph
C 2D classification
D
15.8%
9.3%
30.7%
15.1%
29.2%
E
43.0%
46,947 particles
20.4%
36.6%
39,960 particles
F
49.7%
23,332 particles
48.3%
22,675 particles
2.0%
83.0%
33,149 particles
8.6%
8.4%
G
90°
90°
Membrane
Contour = 0.145
H Fourier shell correlation
GSFSC Resolution: 6.83Å
No Mask (8.8Å)
Loose (7.5Å)
Tight (6.7Å)
Corrected (6.8Å)
FSC0.143 = 6.83 Å
I Direction distribution
Elevation
Azimuth
# of images

Fig. S12. Flowchart of cryo-EM data processing for the membrane-bound DOCK11–Cdc42^{Q61L} binary complex. (*A*) Processing workflow. Particles initially picked using Template Picker (1) were subjected to 2D classification. The classes highlighted by the red boxes were then used as templates for a second round of template-based particle picking (Template Picker (2)) from the same dataset. (*B*) A representative of motion corrected cryo-EM micrographs. (*C*) 2D class averages. (*D*–*F*) 3D density maps obtained from the indicated runs. (*G*) 3D reconstructed map. The cryo-EM map corresponds to the extended conformation of the DOCK11–Cdc42^{Q61L} binary complex and was subsequently used as the template for particle picking of the membrane-bound DOCK11–Cdc42^{G15A}–Cdc42^{Q61L} ternary complex shown in Fig. S13. (*H*) FSC curve of the final cryo-EM map. (*I*) Particle orientation distribution of the final NU refinement.

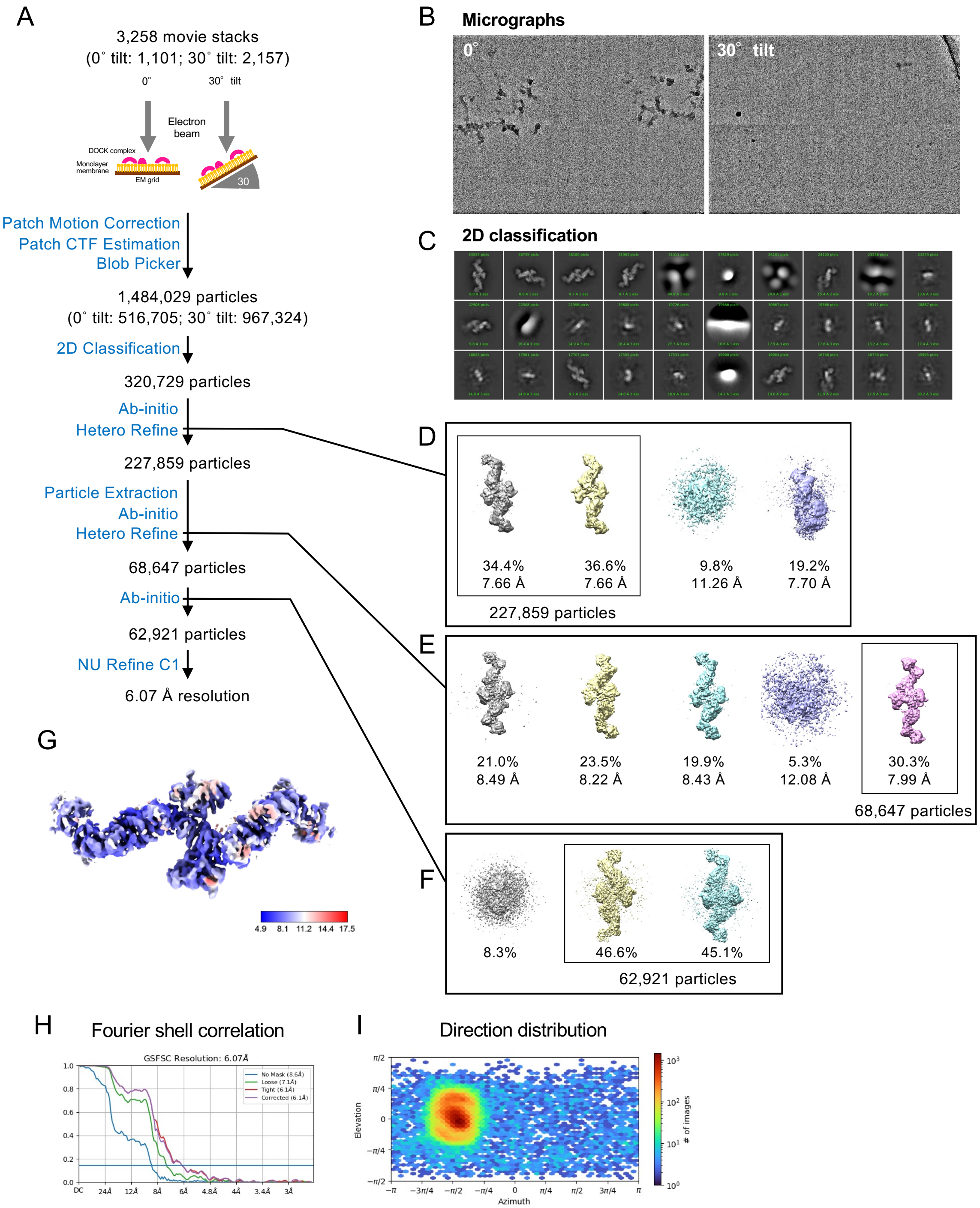

Fig. S13. Flowchart of cryo-EM data processing for the membrane-bound DOCK11–Cdc42^{G15A}–Cdc42^{Q61L} ternary complex. (*A*) Processing workflow for the membrane-bound ternary complex data. (*B*) A representative of motion corrected cryo-EM micrographs. (*C*) 2D class averages of the ternary complex data used to generate an *ab initio* model. (*D–F*) 3D density maps obtained from the indicated runs. (*G*) Local resolution map, (*H*) FSC curve of the final cryo-EM map, and (*I*) particle orientation distribution from the final NU refinement for the "ternary complex" map.

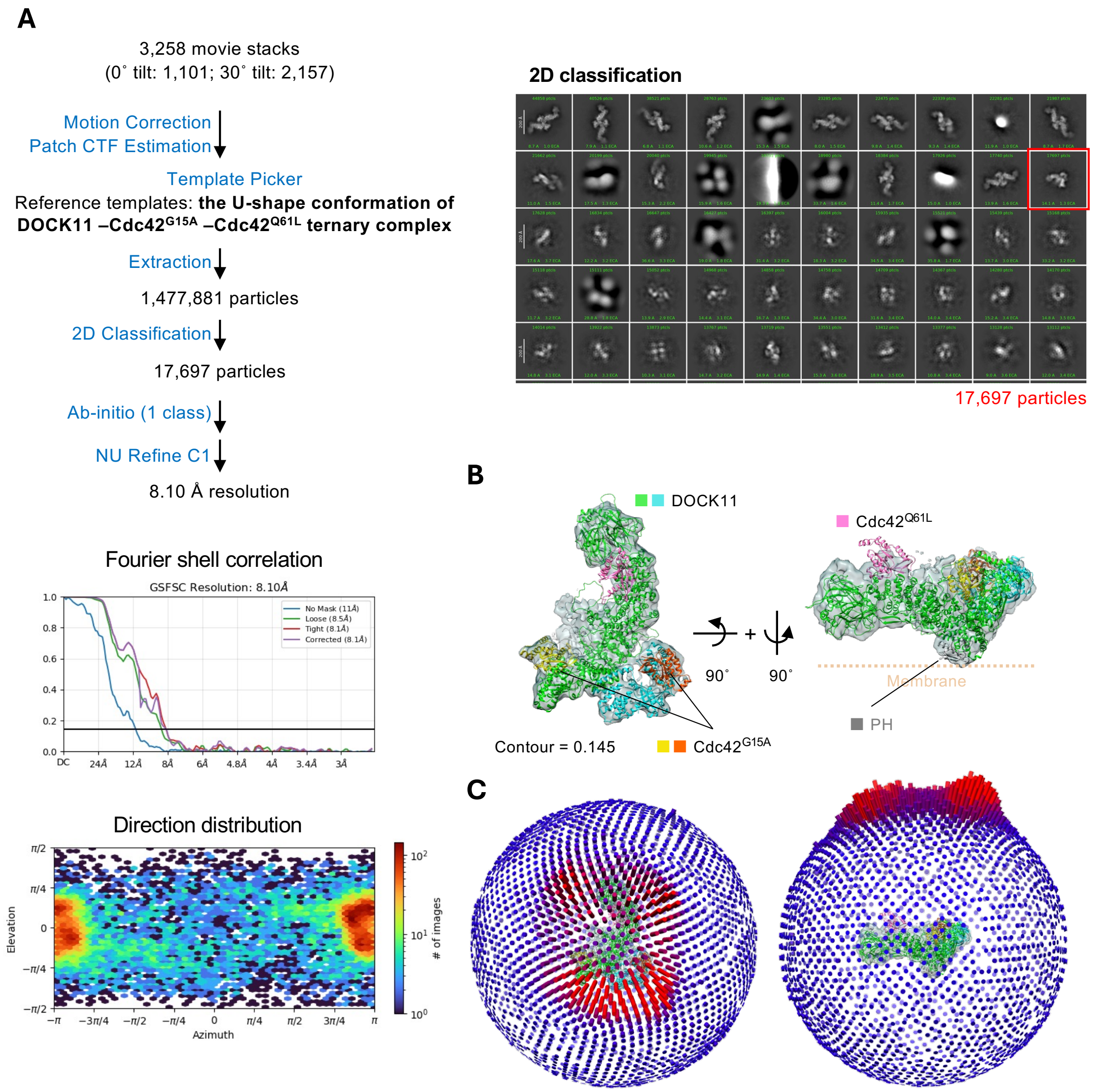

Fig. S14. Cryo-EM data processing for identification of the U-shaped DOCK11–Cdc42^{G15A}–Cdc42^{Q61L} ternary complex in the membrane-coated grid dataset. (A) Flowchart of cryo-EM data processing. Particle picking was performed using the cryo-EM map of the U-shaped DOCK11–Cdc42^{G15A}–Cdc42^{Q61L} ternary complex as the template. Following 2D classification, only the class highlighted by the red box, corresponding to the U-shaped conformation, was selected for subsequent 3D reconstruction. (B) Final cryo-EM map obtained from the selected particles. The atomic models of DOCK11 and Cdc42 were fitted into the density. (C) Preferred particle orientations of the reconstruction shown in (B), visualized using the orientation distribution plot.

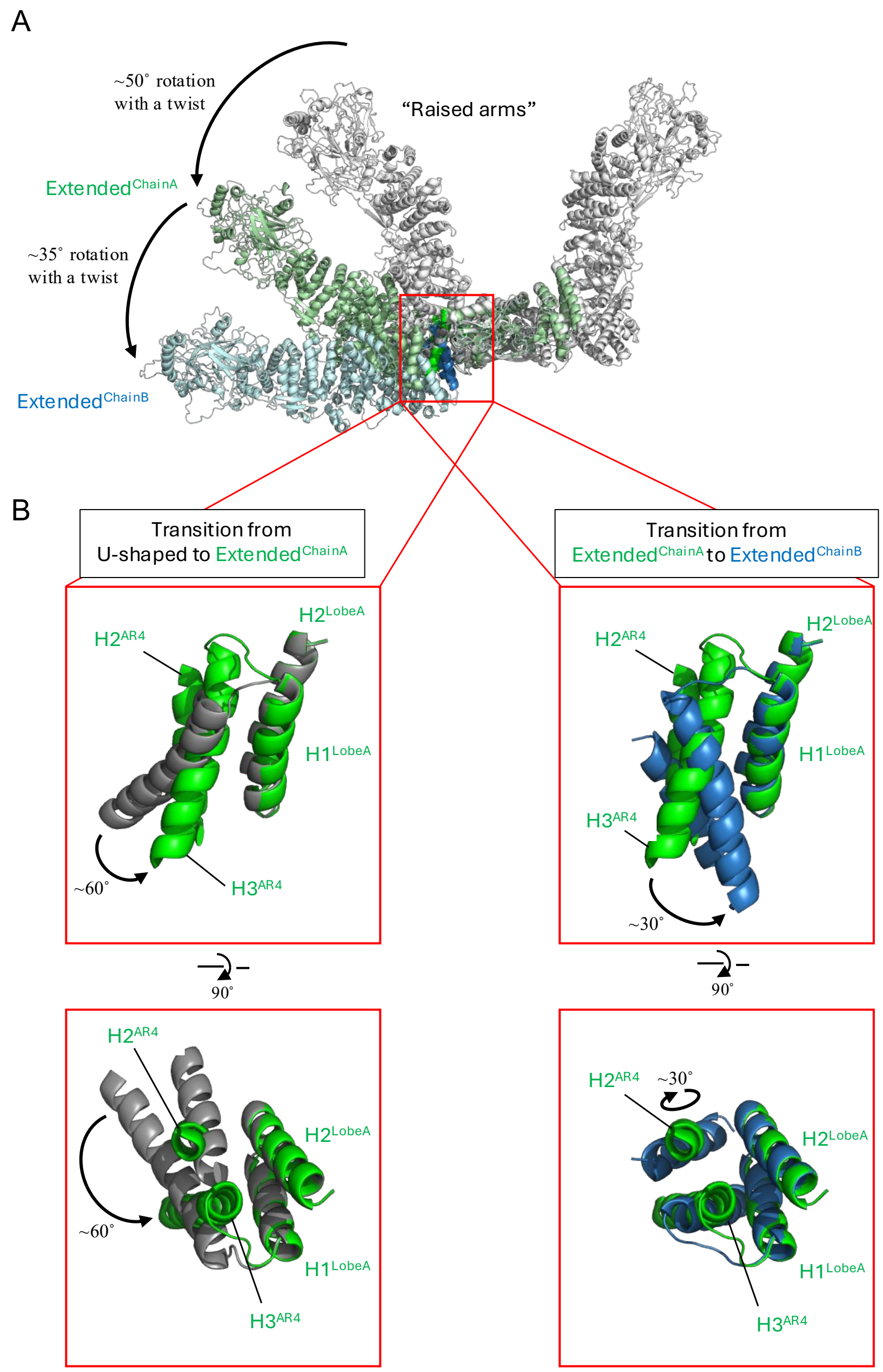

Fig. S15. Conformational transitions of DOCK11 from the U-shaped to the extended structures. (*A*) Structures of DOCK11 protomers (Chain A) of the ternary complex in solution (white) and in the membrane-bound state (Chains A (light green) and B (light cyan)) are superimposed based on the DHR2 domain, showing large conformational transitions of rotations with a twist derived from pivot motions at the ARM–DHR2 domain connection. The N-terminal half (the whole region excluding the DHR2 domain) behaves as a rigid body. The other protomer of DOCK11 in solution (Chain B; white) is also shown. (*B*) Close views of the ARM–DHR2 connection region, showing transitions of two helices from AR4 of the ARM domain ($H2^{AR4}$ and $H3^{AR4}$) and two from Lobe A of the DHR2 domain ($H1^{LobeA}$ and $H2^{LobeB}$) between the U-shaped and extended (Chain A) structures (left panels), and between chains A and B of the extended structure (right panels).

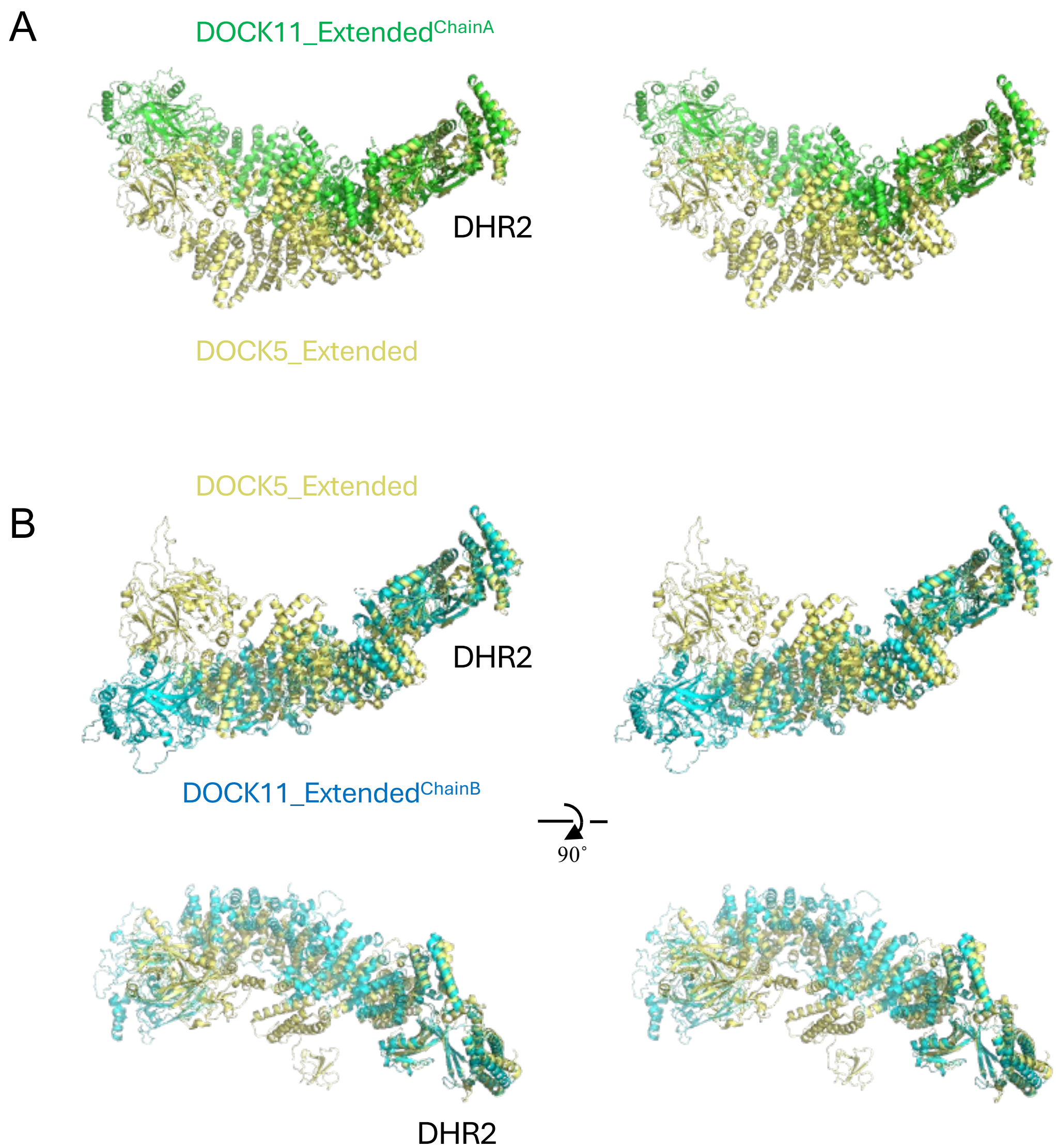

Fig. S16. Comparison of DOCK11 (Chain A (green; *A*) and Chain B (cyan; *B*)) and DOCK5 (9LX0, Chain B; yellow) in the extended conformation (stereo view). Structures are superimposed based on the DHR2 domain. The ARM domains of DOCK11 Chain B and DOCK5 are largely overlapped, though positions of helices are deviated.

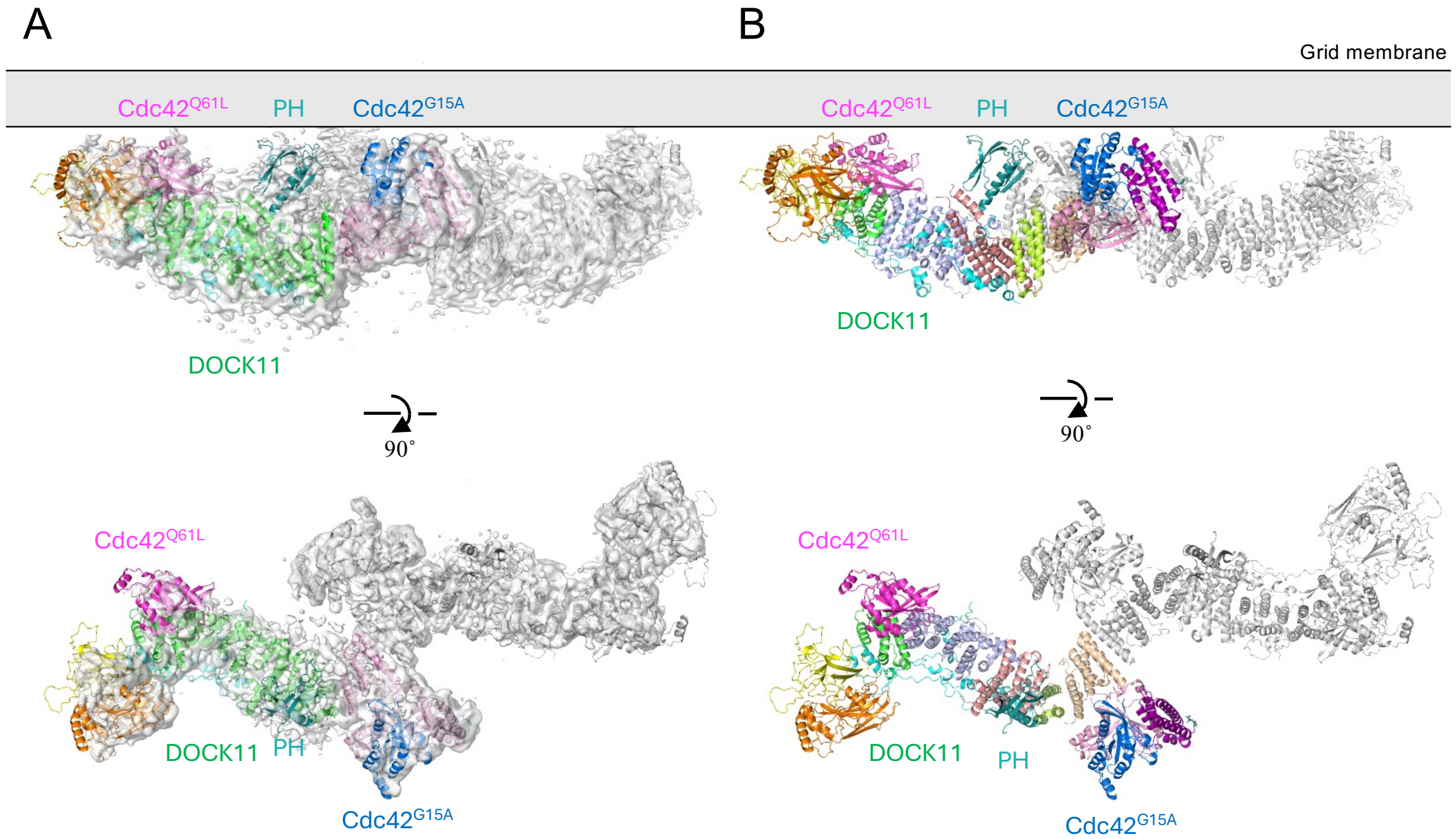

Fig. S17. The PH domain is likely bound to the membrane. (*A*) Cryo-EM density maps and fitted structural models of the DOCK11–Cdc42^{G15A}–Cdc42^{Q61L} ternary complex at a lowered contour level, showing densities between the C-terminal region (AR3) of the ARM domain and the lipid membrane coated on the cryo-EM grid, likely corresponding to the PH domain (calculated by GENESIS (62–64); deep teal). (*B*) The fitted structural model of the ternary complex, showing AR1–AR4 and Lobes A–C colored with the same scheme as Fig. S4.

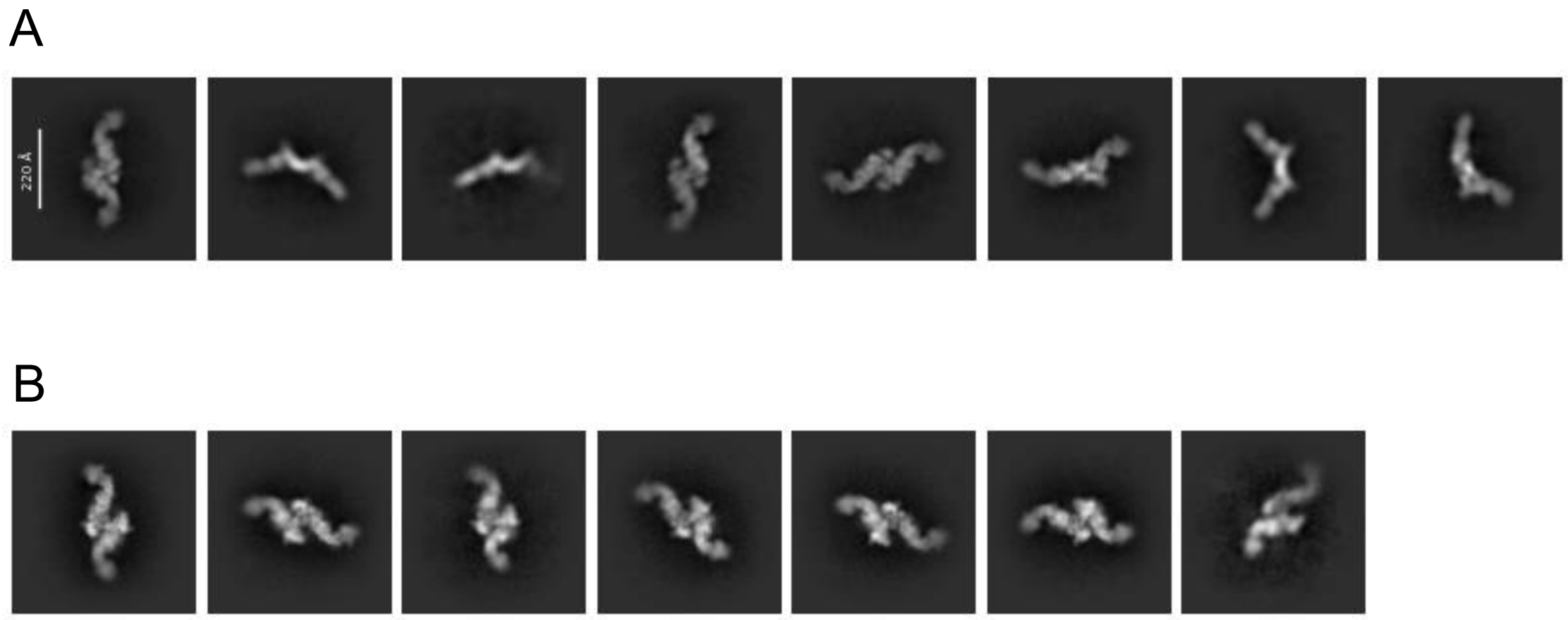

Fig. S18. Extended forms of DOCK11 in solution. 2D images of the extended conformation of DOCK11 for the cryo-EM data of (*A*) the DOCK11 apo sample and (*B*) the “ternary sample”, indicating DOCK11 is in equilibrium between U-shaped and extended conformations in solution.

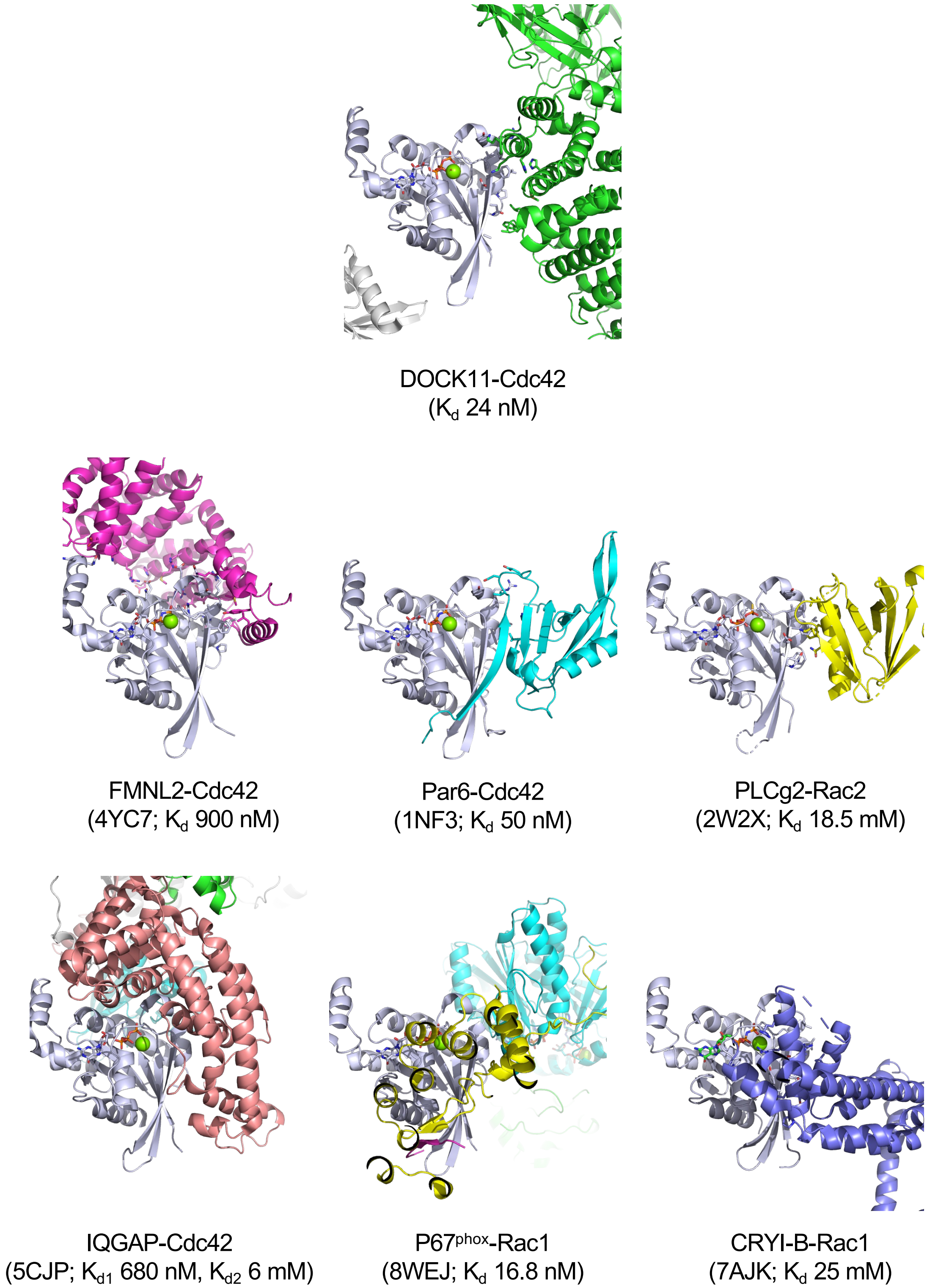

Fig. S19. Comparison of interactions between activated Rho GTPases and their effector proteins. Activated Cdc42 and Rac1 (white blue) are shown in the same orientation. The effector proteins are shown in different colors. PDB IDs and K_d values are shown in parentheses.

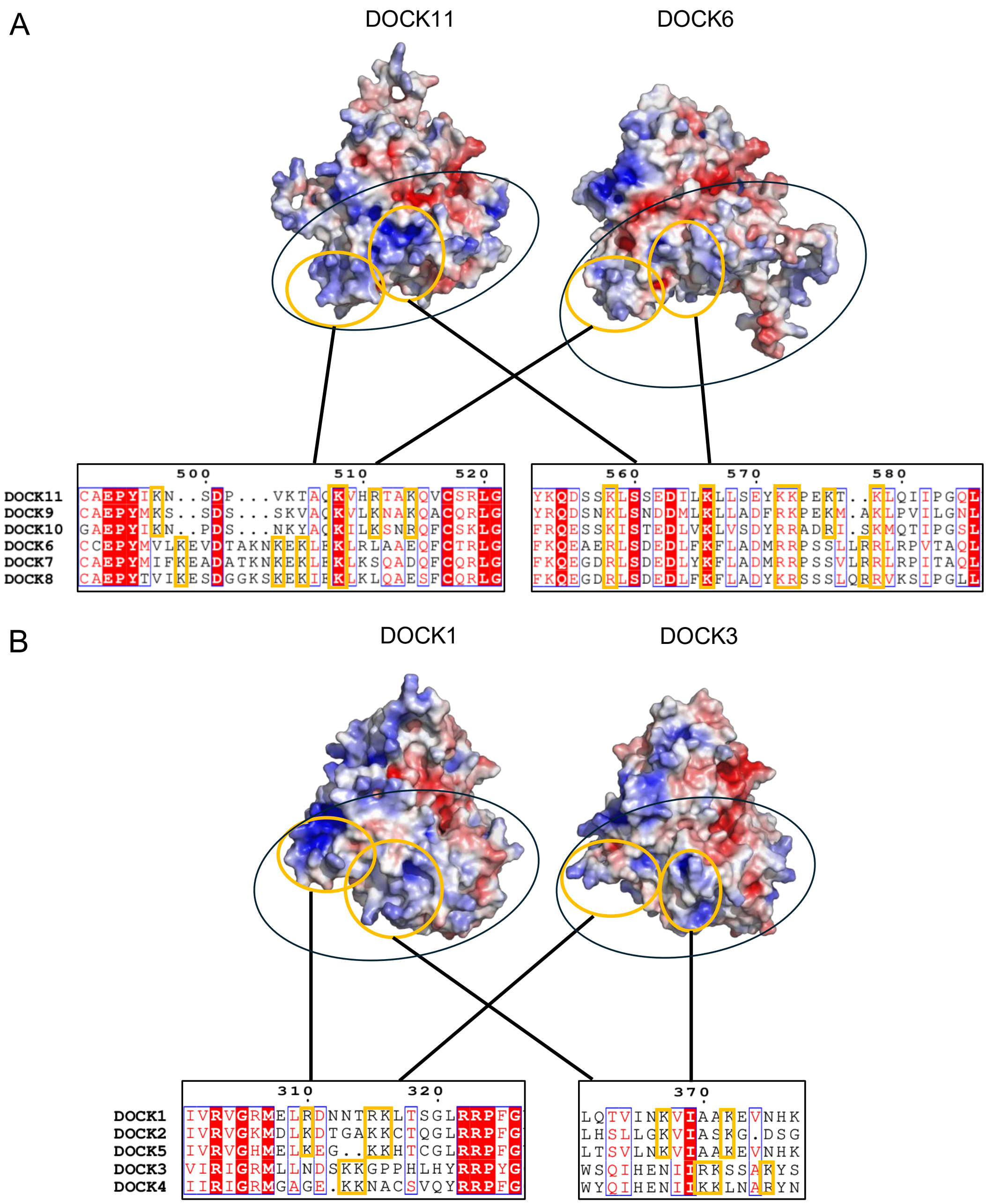

Fig. S20. Conservation of basic regions within the DHR3 domains of the DOCK family. Surface electrostatic potential maps for the structures of the DHR3-DHR1 tandems of (*A*) DOCK11 and DOCK6, and (*B*) DOCK1 and DOCK3 are shown as representatives of the DOCK-D, DOCK-C, DOCK-A and DOCK-B subfamilies, respectively. The DHR3 domains and regions containing conserved basic residues are indicated by black and orange circles, respectively, on the surface potential maps, and the corresponding residues are indicated by orange rectangles in the sequence alignments. The orientation of the tandem structure is roughly the same as in Fig. 4B. Sequence numbers are indicated for (*A*) DOCK11 and (*B*) DOCK1. The structures of the DHR3-DHR1 domains of DOCK1, DOCK3, and DOCK6 were obtained from the AlphaFold-predicted full-length models in the UniProt database (AF-Q14185-F1, AF-Q81ZD9-F1, and AF-Q96HP0-F1, respectively). Surface potential maps were calculated using the APBS plugin in PyMol, and the sequence alignments were generated using Clustal Omega and Espript.

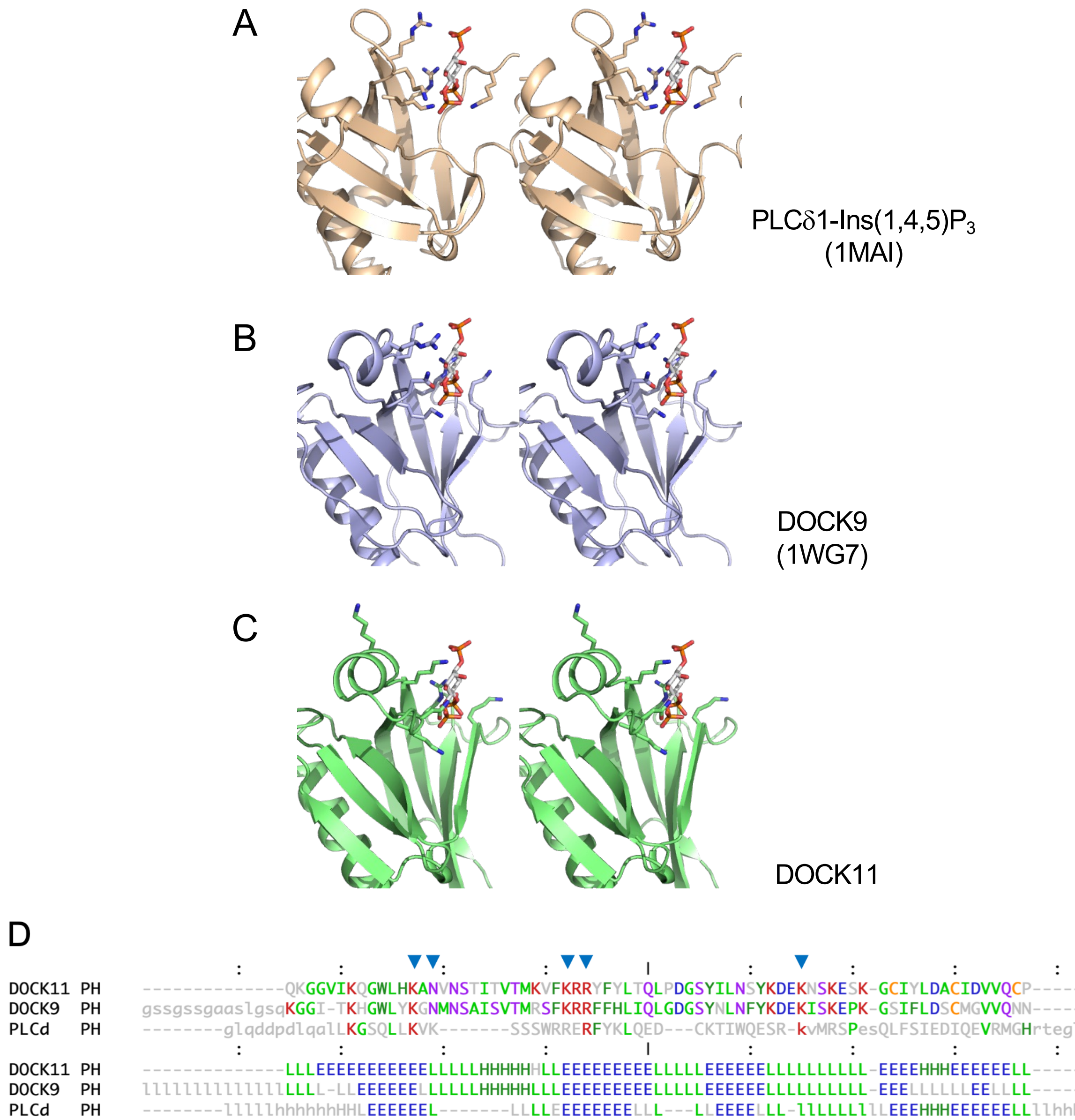

Fig. S21. Conservation of PIP-binding residues of the PH domain of DOCK11, DOCK9, and PLCδ1. (*A*) Crystal structure of the PH domain of PLCδ1 complexed with Ins(1,4,5)P_3 (PDB ID 1MAI). Residues involved in Ins(1,4,5)P_3 binding are shown as sticks. (*B*) NMR structure of the PH domain of DOCK9 (PDB ID 1WG7) and (*C*) an AlphaFold-predicted model of the PH domain of DOCK11. Residues corresponding to the Ins(1,4,5)P_3-binding residues of PLCδ1 are shown as sticks, with the Ins(1,4,5)P_3 model overlaid. Structural models are shown as stereo views. (*D*) Structure-based sequence alignment of DOCK11, DOCK9 and PLCδ1 produced using Dali (79). Secondary structure elements are shown below the sequences (E: strand; H,h: helix; L,l: loop). Only N-terminal regions including PIP-binding residues are shown (residues 162–233 for DOCK11). Positions corresponding to the Ins(1,4,5)P_3-binding residues of PLCδ1 are indicated by triangles (K174, N176, K189, R191 and K218 for DOCK11).

Tables

Table S1. Cryo-EM data collection, model refinement and validation statistics for DOCK11 in solution

**Data collection and processing**			
Dataset	DOCK11 apo (EMD-68711)	The "binary data"	The "ternary data" (EMD-68712*, 68713†)
Magnification	64,000	64,000	64,000
Voltage (kV)	300	300	300
Electron exposure (e–/Å^2)	50.3	50.4	50.0
Defocus range (μm)	–0.80 ~ –0.22	–0.80 ~ –0.20	–0.80 ~ –0.22
Pixel size (Å)	1.33	1.33	1.33
Symmetry imposed	C2	C2	C2
Initial particle images (no.)	3,215,895	2,326,627	5,296,978
Final particle images (no.)	511,163	93,206	117,660* / 141,956†
Map resolution (Å)	3.8	5.2	4.3* / 3.8†
FSC threshold	0.143	0.143	0.143

**Model refinement and validation**			
Dataset	DOCK11 apo	The "ternary data"	
	(PDB 22VD)	The binary complex (PDB 22VE)	The ternary complex (PDB 22VF)
Model composition			
Non-hydrogen atoms	28,286	30,990	33,818
Protein residues	3,486	3,840	4,194
Ligands	–	–	Mg-GTP
B factors (Å^2)			
Protein	230	290	227
Ligand	–	–	171
R.m.s. deviations			
Bond lengths (Å)	0.003	0.004	0.004
Bond angles (°)	0.70	0.66	0.72
Validation			
MolProbity score	1.99	1.91	2.06
Clashscore	14.03	11.32	16.12
Poor rotamers (%)	0.00	0.00	0.05
Ramachandran plot			
Favored (%)	95.10	95.08	94.96
Allowed (%)	4.90	4.92	4.99
Disallowed (%)	0.00	0.00	0.05

*The values for the binary complex density map
†The values for the ternary complex density map

Table S2. Cryo-EM data collection, model refinement and validation statistics for DOCK11 in the membrane-bound state

**Data collection and processing**	
Dataset	The ternary complex (EMD-68714)
Magnification	64,000
Voltage (kV)	300
Electron exposure (e–/Å^2)	49.7
Defocus range (μm)	–0.80 ~ –2.0
Pixel size (Å)	1.33
Symmetry imposed	C1
Initial particle images (no.)	1,484,729
Final particle images (no.)	62,921
Map resolution (Å)	6.1
FSC threshold	0.143

**Model refinement and validation**	
Dataset	The ternary complex (PDB 22VG)
Map sharpening *B* factor (Å^2)	–327.3
Model composition	
Non-hydrogen atoms	33,818
Protein residues	4,194
Ligands	GTP
B factors (Å^2)	
Protein	376
Ligand	143
R.m.s. deviations	
Bond lengths (Å)	0.003
Bond angles (°)	0.75
Validation	
MolProbity score	2.12
Clashscore	19.18
Poor rotamers (%)	0.03
Ramachandran plot	
Favored (%)	95.04
Allowed (%)	4.96
Disallowed (%)	0.00

Table S3. BS3 cross-linking mass spectrometry of the DOCK11-Cdc42(G15A)-Cdc42(Q61L) ternary complex

Three independent experiments (combined)

CSMs: Cross-link Spectrum Matches

# CSMs	Max. XlinkX Score	Protein 1, Domain		Residue 1	Protein 2, Domain		Residue 2	Chain 1	Chain 2	Cα-Cα distance (Å)
6	46.62	DOCK11	AWS1	36	DOCK11	ARM	983	A	A	38.725
8	61.26	DOCK11	AWS1	36	DOCK11	ARM	1092	A	A	33.888
9	118.03	DOCK11	AWS1	38	DOCK11	AWS1	53	A	A	29.468
4	52.68	DOCK11	AWS1	38	DOCK11	ARM	983	A	A	35.302
1	49.67	DOCK11	AWS1	38	DOCK11	DHR2	1984	A	B	52.784
13	259.95	DOCK11	AWS1	38	DOCK11	DHR2	2006	A	B	20.35
10	126.05	DOCK11	AWS1	53	DOCK11	AWS1	36	A	A	34.644
318	98.37	DOCK11	AWS1	53	DOCK11	ARM	983	A	A	15.305
11	61.26	DOCK11	AWS1	53	DOCK11	ARM	1092	A	A	16.785
5	43.07	DOCK11	AWS1	53	DOCK11	ARM	1157	A	A	25.021
1	58.64	DOCK11	AWS1	94	DOCK11	AWS1	53	A	A	53.188
114	114.02	DOCK11	AWS1	102	DOCK11	AWS1	94	A	A	8.744
12	50.91	DOCK11	AWS1	102	DOCK11	ARM	1591	A	A	100.806
32	144.83	DOCK11	AWS1	106	DOCK11	AWS1	94	A	A	11.462
2	37.72	DOCK11	AWS1	106	DOCK11	ARM	1591	A	A	95.827
1	45.08	DOCK11	AWS1	118	DOCK11	AWS1	38	A	A	35.749
15	147.12	DOCK11	AWS1	118	DOCK11	AWS1	53	A	A	23.992
16	114.02	DOCK11	AWS1	118	DOCK11	AWS1	132	A	A	17.998
3	60.36	DOCK11	AWS1	118	DOCK11	AWS2	327	A	A	40.678
20	61.26	DOCK11	AWS1	118	DOCK11	ARM	983	A	A	17.162
3	66.99	DOCK11	AWS1	118	DOCK11	ARM	1349	A	A	Not modeled
7	59.64	DOCK11	AWS1	132	DOCK11	ARM	1157	A	A	19.873
3	86.11	DOCK11	PH	152	DOCK11	ARM	1493	A	A	Not modeled
2	114.93	DOCK11	PH	163	DOCK11	PH	269	A	A	Not modeled
1	38.32	DOCK11	PH	174	DOCK11	ARM	1578	A	A	Not modeled
3	38.19	DOCK11	PH	186	DOCK11	ARM	1578	A	A	Not modeled
14	114.67	DOCK11	PH	186	DOCK11	DHR2	1824	A	A	Not modeled
2	103.74	DOCK11	PH	209	DOCK11	DHR3	536	A	A	Not modeled
7	97.03	DOCK11	PH	209	DOCK11	ARM	1574	A	A	Not modeled
1	49.42	DOCK11	PH	209	DOCK11	ARM	1591	A	A	Not modeled
2	113.18	DOCK11	PH	209	DOCK11	ARM	1595	A	A	Not modeled
10	58.66	DOCK11	PH	209	DOCK11	DHR2	1801	A	A	Not modeled
8	54.53	DOCK11	PH	209	DOCK11	DHR2	1824	A	A	Not modeled
1	41.44	DOCK11	PH	215	DOCK11	ARM	1578	A	A	Not modeled
16	77.53	DOCK11	PH	243	DOCK11	ARM	1578	A	A	Not modeled
14	91.96	DOCK11	PH	247	DOCK11	PH	282	A	A	Not modeled
13	220.93	DOCK11	PH	247	DOCK11	PH	283	A	A	Not modeled
1	61.11	DOCK11	PH	247	DOCK11	PH	299	A	A	Not modeled
12	103.92	DOCK11	PH	247	DOCK11	ARM	1574	A	A	Not modeled
7	51.46	DOCK11	PH	268	DOCK11	PH	243	A	A	Not modeled
27	146.35	DOCK11	PH	268	DOCK11	PH	282	A	A	Not modeled
18	83.61	DOCK11	PH	269	DOCK11	PH	168	A	A	Not modeled
1	43.7	DOCK11	PH	269	DOCK11	AWS2	327	A	A	Not modeled
4	94.67	DOCK11	PH	269	DOCK11	ARM	1349	A	A	Not modeled
1	100.15	DOCK11	PH	269	DOCK11	ARM	1574	A	A	Not modeled
10	92.69	DOCK11	PH	282	DOCK11	PH	243	A	A	Not modeled
6	58.55	DOCK11	PH	282	DOCK11	ARM	1517	A	A	Not modeled
9	47.14	DOCK11	PH	282	DOCK11	ARM	1519	A	A	Not modeled
4	50.04	DOCK11	PH	282	DOCK11	ARM	1574	A	A	Not modeled
3	51	DOCK11	PH	282	DOCK11	ARM	1578	A	A	Not modeled
3	84.36	DOCK11	PH	283	DOCK11	PH	243	A	A	Not modeled
23	296.64	DOCK11	PH	283	DOCK11	PH	269	A	A	Not modeled
6	54.76	DOCK11	PH	283	DOCK11	ARM	1157	A	A	Not modeled
2	44.38	DOCK11	PH	283	DOCK11	ARM	1342	A	A	Not modeled
6	80.38	DOCK11	PH	283	DOCK11	ARM	1473	A	A	Not modeled
19	150.63	DOCK11	PH	283	DOCK11	ARM	1517	A	A	Not modeled

19	61.26	DOCK11	PH	283	DOCK11	ARM	1519	A	A	Not modeled
2	41.96	DOCK11	PH	283	DOCK11	ARM	1578	A	A	Not modeled
6	166.17	DOCK11	PH	299	DOCK11	PH	269	A	A	Not modeled
5	72.55	DOCK11	PH	299	DOCK11	PH	282	A	A	Not modeled
3	62.71	DOCK11	PH	299	DOCK11	AWS2	327	A	A	Not modeled
3	49.44	DOCK11	PH	299	DOCK11	ARM	1454	A	A	Not modeled
9	77.74	DOCK11	PH	299	DOCK11	ARM	1466	A	A	Not modeled
38	186.74	DOCK11	PH	299	DOCK11	ARM	1473	A	A	Not modeled
32	118.18	DOCK11	PH	299	DOCK11	ARM	1517	A	A	Not modeled
30	61.26	DOCK11	PH	299	DOCK11	ARM	1519	A	A	Not modeled
3	65.44	DOCK11	PH	299	DOCK11	ARM	1578	A	A	Not modeled
2	56.01	DOCK11	AWS2	317	DOCK11	AWS2	327	A	A	15.218
14	132.22	DOCK11	AWS2	317	DOCK11	ARM	1517	A	A	23.858
50	148.37	DOCK11	AWS2	327	DOCK11	ARM	1157	A	A	16.869
1	47.07	DOCK11	AWS2	357	DOCK11	AWS2	327	A	A	53.644
1	68.22	DOCK11	AWS2	357	DOCK11	DHR3	536	A	A	32.677
22	118.01	DOCK11	AWS2	357	DOCK11	ARM	917	A	A	8.422
8	184.08	DOCK11	AWS2	357	DOCK11	ARM	1349	A	A	Not modeled
9	40.57	DOCK11	DHR3	406	DOCK11	DHR3	572	A	A	13.639
1	50.61	DOCK11	DHR3	442	DOCK11	AWS1	94	A	A	39.549
19	67.06	DOCK11	DHR3	442	DOCK11	DHR3	514	A	A	25.177
9	141.46	DOCK11	DHR3	442	DOCK11	DHR1	747	A	A	34.497
6	50.34	DOCK11	DHR3	483	DOCK11	DHR3	508	A	A	16.21
1	58.66	DOCK11	DHR3	483	DOCK11	DHR3	514	A	A	14.247
24	186.29	DOCK11	DHR3	483	DOCK11	DHR3	553	A	A	11.759
37	81.22	DOCK11	DHR3	498	DOCK11	DHR3	508	A	A	12.651
8	92.28	DOCK11	DHR3	498	DOCK11	DHR3	514	A	A	20.049
50	182.31	DOCK11	DHR3	498	DOCK11	DHR3	553	A	A	12.527
1	37.35	DOCK11	DHR3	498	DOCK11	DHR3	576	A	A	22.71
2	84.36	DOCK11	DHR3	504	DOCK11	DHR3	514	A	A	15.073
1	88.73	DOCK11	DHR3	508	DOCK11	DHR3	504	A	A	6.111
4	90.32	DOCK11	DHR3	508	DOCK11	DHR1	660	A	A	27.34
2	51	DOCK11	DHR3	508	DOCK11	DHR1	746	A	A	25.926
14	147.63	DOCK11	DHR3	514	DOCK11	DHR1	508	A	A	9.781
4	40.91	DOCK11	DHR3	514	DOCK11	DHR1	746	A	A	21.639
42	219.4	DOCK11	DHR3	536	DOCK11	DHR3	572	A	A	15.33
5	197.65	DOCK11	DHR3	536	DOCK11	DHR3	573	A	A	18.908
3	36.87	DOCK11	DHR3	553	DOCK11	PH	212	A	A	Not modeled
28	101.25	DOCK11	DHR3	553	DOCK11	DHR3	508	A	A	21.181
20	116.12	DOCK11	DHR3	553	DOCK11	DHR3	514	A	A	23.91
12	66.56	DOCK11	DHR3	553	DOCK11	DHR3	576	A	A	15.055
1	49.37	DOCK11	DHR3	558	DOCK11	DHR3	573	A	A	17.205
10	87.26	DOCK11	DHR3	558	DOCK11	DHR3	576	A	A	15.965
23	76.27	DOCK11	DHR3	566	DOCK11	DHR3	576	A	A	12.875
17	146.62	DOCK11	DHR3	566	DOCK11	DHR1	812	A	A	20.171
12	95.62	DOCK11	DHR3	572	DOCK11	DHR3	576	A	A	9.162
1	50.97	DOCK11	DHR3	578	DOCK11	DHR3	572	A	A	12.874
9	79.91	DOCK11	DHR3	609	DOCK11	DHR3	536	A	A	29.99
16	156.12	DOCK11	DHR1	651	DOCK11	DHR1	812	A	A	8.514
3	47.63	DOCK11	DHR1	660	DOCK11	DHR1	746	A	A	17.11
1	36.28	DOCK11	DHR1	660	DOCK11	DHR1	812	A	A	21.588
1	37.56	DOCK11	DHR1	660	DOCK11	DHR2	1657	A	A	156.553
34	210.26	DOCK11	DHR1	682	DOCK11	DHR3	442	A	A	25.159
22	79.03	DOCK11	DHR1	747	DOCK11	DHR3	514	A	A	23.937
19	55.13	DOCK11	DHR1	747	DOCK11	DHR1	660	A	A	18.355
17	78.62	DOCK11	DHR1	802	DOCK11	DHR1	656	A	A	13.446
35	94.27	DOCK11	DHR1	802	DOCK11	DHR1	660	A	A	17.722
1	57.71	DOCK11	DHR1	802	DOCK11	DHR1	812	A	A	20.367
45	111.69	DOCK11	DHR1	808	DOCK11	DHR1	651	A	A	9.91
2	67.89	DOCK11	DHR1	808	DOCK11	DHR1	656	A	A	12.09

2	35.3	DOCK11	ARM	830	DOCK11	ARM	851	A	A	10.076
1	36.2	DOCK11	ARM	830	DOCK11	DHR2	1925	A	A	120.076
1	58.64	DOCK11	ARM	981	DOCK11	AWS1	53	A	A	19.253
1	48.17	DOCK11	ARM	1032	DOCK11	ARM	1349	A	A	Not modeled
6	46.8	DOCK11	ARM	1057	DOCK11	AWS1	36	A	A	10.269
19	279.98	DOCK11	ARM	1057	DOCK11	AWS1	38	A	A	15.085
5	52.94	DOCK11	ARM	1057	DOCK11	DHR2	2006	A	A	61.827
2	143.22	DOCK11	ARM	1060	DOCK11	AWS1	36	A	A	17.309
17	181.94	DOCK11	ARM	1060	DOCK11	ARM	1349	A	A	Not modeled
4	72.02	DOCK11	ARM	1111	DOCK11	AWS1	36	A	A	16.261
26	236.77	DOCK11	ARM	1111	DOCK11	AWS1	38	A	A	10.619
24	251.58	DOCK11	ARM	1111	DOCK11	AWS1	53	A	A	19.119
1	43	DOCK11	ARM	1142	DOCK11	ARM	1349	A	A	Not modeled
4	46.62	DOCK11	ARM	1157	DOCK11	ARM	1092	A	A	27.945
8	101.52	DOCK11	ARM	1162	DOCK11	AWS1	53	A	A	19.672
3	123.34	DOCK11	ARM	1219	DOCK11	AWS1	36	A	A	Not modeled
1	99.51	DOCK11	ARM	1219	DOCK11	ARM	1060	A	A	Not modeled
2	69.95	DOCK11	ARM	1219	DOCK11	ARM	1233	A	A	Not modeled
10	138.9	DOCK11	ARM	1219	DOCK11	ARM	1342	A	A	Not modeled
4	195.41	DOCK11	ARM	1219	DOCK11	ARM	1349	A	A	Not modeled
8	152.97	DOCK11	ARM	1219	DOCK11	ARM	1434	A	A	Not modeled
5	168.06	DOCK11	ARM	1219	DOCK11	ARM	1493	A	A	Not modeled
1	36.2	DOCK11	ARM	1219	DOCK11	ARM	1517	A	A	Not modeled
3	140.79	DOCK11	ARM	1219	DOCK11	DHR2	1619	A	A	Not modeled
7	189.32	DOCK11	ARM	1219	DOCK11	DHR2	1734	A	A	Not modeled
4	114.84	DOCK11	ARM	1219	DOCK11	DHR2	1788	A	A	Not modeled
20	183.32	DOCK11	ARM	1219	DOCK11	DHR2	1801	A	A	Not modeled
23	104.48	DOCK11	ARM	1219	DOCK11	DHR2	1851	A	A	Not modeled
2	47.29	DOCK11	ARM	1219	DOCK11	DHR2	1989	A	A	Not modeled
2	121.5	DOCK11	ARM	1219	DOCK11	DHR2	2006	A	A	Not modeled
8	67.89	DOCK11	ARM	1233	DOCK11	AWS1	36	A	A	Not modeled
7	109.88	DOCK11	ARM	1233	DOCK11	ARM	1060	A	A	Not modeled
23	195.41	DOCK11	ARM	1233	DOCK11	ARM	1349	A	A	Not modeled
14	124.32	DOCK11	ARM	1233	DOCK11	ARM	1493	A	A	Not modeled
4	71.07	DOCK11	ARM	1233	DOCK11	ARM	1517	A	A	Not modeled
8	97.6	DOCK11	ARM	1233	DOCK11	DHR2	1734	A	A	Not modeled
9	68.22	DOCK11	ARM	1233	DOCK11	DHR2	1788	A	A	Not modeled
17	72	DOCK11	ARM	1233	DOCK11	DHR2	1801	A	A	Not modeled
10	63.05	DOCK11	ARM	1233	DOCK11	DHR2	1851	A	A	Not modeled
1	48.28	DOCK11	ARM	1342	DOCK11	PH	269	A	A	Not modeled
24	219.93	DOCK11	ARM	1342	DOCK11	ARM	1060	A	A	25.65
31	232.39	DOCK11	ARM	1342	DOCK11	ARM	1349	A	A	Not modeled
8	142.63	DOCK11	ARM	1342	DOCK11	ARM	1493	A	A	33.094
20	70.86	DOCK11	ARM	1349	DOCK11	ARM	1329	A	A	Not modeled
13	118.18	DOCK11	ARM	1349	DOCK11	DHR2	1801	A	A	Not modeled
1	78.19	DOCK11	ARM	1349	DOCK11	DHR2	1851	A	A	Not modeled
3	83.01	DOCK11	ARM	1434	DOCK11	ARM	1233	A	A	Not modeled
15	150.82	DOCK11	ARM	1434	DOCK11	DHR2	1734	A	A	22.66
2	62.84	DOCK11	ARM	1445	DOCK11	ARM	1349	A	A	Not modeled
28	237.64	DOCK11	ARM	1445	DOCK11	ARM	1434	A	A	15.998
38	130.28	DOCK11	ARM	1454	DOCK11	AWS2	327	A	A	12.53
18	148.37	DOCK11	ARM	1466	DOCK11	AWS2	327	A	A	15.834
7	119.54	DOCK11	ARM	1473	DOCK11	ARM	1519	A	A	14.185
1	62.39	DOCK11	ARM	1473	DOCK11	DHR2	1851	A	B	29.815
6	85.52	DOCK11	ARM	1493	DOCK11	PH	215	A	A	Not modeled
31	68.6	DOCK11	ARM	1493	DOCK11	ARM	1329	A	A	14.929
21	159.65	DOCK11	ARM	1493	DOCK11	ARM	1349	A	A	Not modeled
24	126.38	DOCK11	ARM	1517	DOCK11	ARM	1473	A	A	9.047
34	98.37	DOCK11	ARM	1517	DOCK11	ARM	1519	A	A	8.292
17	150.17	DOCK11	ARM	1565	DOCK11	PH	282	A	A	Not modeled

19	224.42	DOCK11	ARM	1565	DOCK11	PH	283	A	A	Not modeled
6	167.2	DOCK11	ARM	1565	DOCK11	PH	299	A	A	Not modeled
19	149.11	DOCK11	ARM	1591	DOCK11	ARM	1493	A	A	19.116
1	36.42	DOCK11	ARM	1591	DOCK11	DHR2	1756	A	A	41.458
34	131.72	DOCK11	ARM	1595	DOCK11	DHR2	1801	A	A	11.372
1	43.08	DOCK11	ARM	1595	DOCK11	DHR2	1899	A	A	16.448
1	36.77	DOCK11	ARM	1595	DOCK11	DHR2	1913	A	A	44.804
4	38.19	DOCK11	DHR2	1609	DOCK11	ARM	1578	A	A	20.13
1	56.94	DOCK11	DHR2	1609	DOCK11	DHR2	1780	A	A	19.273
14	130.64	DOCK11	DHR2	1609	DOCK11	DHR2	1899	A	A	13.437
3	126.05	DOCK11	DHR2	1619	DOCK11	ARM	1517	A	A	29.272
7	70.29	DOCK11	DHR2	1619	DOCK11	ARM	1519	A	A	26.754
9	102.87	DOCK11	DHR2	1656	DOCK11	ARM	1519	A	A	24.549
3	42.11	DOCK11	DHR2	1656	DOCK11	DHR2	1657	A	A	3.821
7	110.63	DOCK11	DHR2	1656	DOCK11	DHR2	1728	A	A	18.531
2	41.57	DOCK11	DHR2	1657	DOCK11	ARM	1519	A	A	27.961
3	69.64	DOCK11	DHR2	1657	DOCK11	DHR2	1851	A	A	37.486
30	174.15	DOCK11	DHR2	1668	DOCK11	DHR2	1728	A	A	23.748
6	186.87	DOCK11	DHR2	1720	DOCK11	DHR2	1668	A	A	11.451
18	90.29	DOCK11	DHR2	1720	DOCK11	DHR2	1728	A	A	13.773
1	61.14	DOCK11	DHR2	1728	DOCK11	DHR2	1619	A	A	26.591
17	148.28	DOCK11	DHR2	1728	DOCK11	DHR2	1657	A	A	16.663
2	46.35	DOCK11	DHR2	1728	DOCK11	DHR2	1785	B	A	28.585
18	92.15	DOCK11	DHR2	1728	DOCK11	DHR2	1851	B	A	10.703
14	72.23	DOCK11	DHR2	1734	DOCK11	DHR2	1728	A	A	12.516
2	94.27	DOCK11	DHR2	1748	DOCK11	DHR2	1851	A	A	18.369
49	131.63	DOCK11	DHR2	1756	DOCK11	DHR2	1851	A	A	14.936
12	76.59	DOCK11	DHR2	1780	DOCK11	DHR2	1824	A	A	15.375
15	84.57	DOCK11	DHR2	1780	DOCK11	DHR2	1899	A	A	15.079
1	125.31	DOCK11	DHR2	1785	DOCK11	DHR2	1748	A	A	23.707
3	59.61	DOCK11	DHR2	1785	DOCK11	DHR2	1824	A	A	28.275
1	72.73	DOCK11	DHR2	1785	DOCK11	DHR2	1851	A	A	21.706
3	50.04	DOCK11	DHR2	1788	DOCK11	DHR2	1728	A	A	28.421
25	164.53	DOCK11	DHR2	1788	DOCK11	DHR2	1748	A	A	17.636
16	113.07	DOCK11	DHR2	1788	DOCK11	DHR2	1756	A	A	12.897
6	154.74	DOCK11	DHR2	1788	DOCK11	DHR2	1801	A	A	20.361
3	97.87	DOCK11	DHR2	1801	DOCK11	PH	215	A	A	Not modeled
10	142.27	DOCK11	DHR2	1813	DOCK11	DHR2	1801	A	A	11.14
12	186.74	DOCK11	DHR2	1813	DOCK11	DHR2	1824	A	A	15.077
4	168.06	DOCK11	DHR2	1820	DOCK11	DHR2	1875	A	A	14.086
7	80.22	DOCK11	DHR2	1824	DOCK11	DHR2	1874	A	A	21.609
45	240.66	DOCK11	DHR2	1829	DOCK11	DHR2	1813	A	A	9.666
2	76.59	DOCK11	DHR2	1839	DOCK11	DHR2	1728	B	A	26.547
19	163.53	DOCK11	DHR2	1839	DOCK11	DHR2	1756	A	A	18.662
4	42.87	DOCK11	DHR2	1839	DOCK11	DHR2	1845	A	A	17.428
14	167.75	DOCK11	DHR2	1839	DOCK11	DHR2	1851	A	A	17.065
35	142.42	DOCK11	DHR2	1845	DOCK11	DHR2	1728	B	A	16.017
2	67.03	DOCK11	DHR2	1845	DOCK11	DHR2	1756	A	A	24.296
48	182.75	DOCK11	DHR2	1845	DOCK11	DHR2	1851	A	A	10.556
1	79.02	DOCK11	DHR2	1845	DOCK11	DHR2	1978	A	A	23.894
6	83.61	DOCK11	DHR2	1874	DOCK11	DHR2	1820	A	A	15.06
4	46.57	DOCK11	DHR2	1899	DOCK11	DHR2	1728	B	A	29.999
3	39.73	DOCK11	DHR2	1900	DOCK11	DHR2	1974	A	A	47.209
1	40.72	DOCK11	DHR2	1900	DOCK11	DHR2	1978	A	A	44.023
4	97.87	DOCK11	DHR2	1900	DOCK11	DHR2	1979	A	A	41.307
3	50.28	DOCK11	DHR2	1913	DOCK11	DHR2	1445	A	B	68.273
9	95.62	DOCK11	DHR2	1913	DOCK11	DHR2	1820	A	A	19.574
2	38.1	DOCK11	DHR2	1913	DOCK11	DHR2	1839	A	A	20.641
13	58.92	DOCK11	DHR2	1913	DOCK11	DHR2	1845	A	A	24.281
6	151.75	DOCK11	DHR2	1913	DOCK11	DHR2	1874	A	A	14.331

151	314.84	DOCK11	DHR2	1913	DOCK11	DHR2	1974	A	A	12.808
96	105.53	DOCK11	DHR2	1913	DOCK11	DHR2	1978	A	A	14.69
96	156.85	DOCK11	DHR2	1913	DOCK11	DHR2	1979	A	A	11.505
10	72.23	DOCK11	DHR2	1913	DOCK11	DHR2	1984	A	A	17.183
1	58.67	DOCK11	DHR2	1913	DOCK11	DHR2	1989	A	A	21.817
2	39.92	DOCK11	DHR2	1923	DOCK11	ARM	1445	A	B	54.359
13	87.95	DOCK11	DHR2	1923	DOCK11	DHR2	1978	A	A	15.632
10	113.99	DOCK11	DHR2	1923	DOCK11	DHR2	1979	A	A	12.728
1	40.11	DOCK11	DHR2	1925	DOCK11	DHR3	508	A	A	153.186
1	38.81	DOCK11	DHR2	1925	DOCK11	DHR2	1923	A	A	5.405
48	225.26	DOCK11	DHR2	1931	DOCK11	DHR2	1756	A	A	14.487
24	222.55	DOCK11	DHR2	1931	DOCK11	DHR2	1839	A	A	18.803
137	81.36	DOCK11	DHR2	1931	DOCK11	DHR2	1851	A	A	13.999
14	205.14	DOCK11	DHR2	1931	DOCK11	DHR2	1989	A	A	13.365
4	48.04	DOCK11	DHR2	1974	DOCK11	DHR2	1874	A	A	22.487
8	118.03	DOCK11	DHR2	1974	DOCK11	DHR2	1875	A	A	23.256
12	78.19	DOCK11	DHR2	1974	DOCK11	DHR2	1979	A	A	10.635
7	126.05	DOCK11	DHR2	1984	DOCK11	AWS1	36	A	B	57.383
6	61.3	DOCK11	DHR2	1984	DOCK11	DHR2	1978	A	A	9.896
4	97.07	DOCK11	DHR2	1989	DOCK11	DHR2	1845	A	A	22.873
9	95.35	DOCK11	DHR2	1989	DOCK11	DHR2	1851	A	A	23.257
9	85.31	DOCK11	DHR2	1989	DOCK11	DHR2	1984	A	A	8.692
24	150.89	DOCK11	DHR2	2006	DOCK11	AWS1	36	A	B	24.632
12	70.86	DOCK11	DHR2	2006	DOCK11	ARM	1092	A	B	20.753
11	221.53	DOCK11	DHR2	2006	DOCK11	ARM	1233	A	A	Not modeled
1	38.09	DOCK11	DHR2	2006	DOCK11	ARM	1591	A	A	64.183
33	126.05	DOCK11	DHR2	2017	DOCK11	AWS1	36	A	B	38.753
20	205.57	DOCK11	DHR2	2017	DOCK11	AWS1	38	A	B	33.741
24	86.16	DOCK11	DHR2	2017	DOCK11	ARM	1092	A	B	19.221
3	76.27	DOCK11	AWS1	53	Cdc42(G15A);Cdc42(Q61L)		150	A	D	57.378
2	77.93	DOCK11	AWS1	53	Cdc42(Q61L);Cdc42(G15A)		150	A	D	57.378
2	66.07	DOCK11	PH	209	Cdc42(G15A);Cdc42(Q61L)		163	A	C	Not modeled
1	43.77	DOCK11	PH	209	Cdc42(Q61L);Cdc42(G15A)		163	A	E	Not modeled
1	38.29	DOCK11	DHR3	406	Cdc42(Q61L);Cdc42(G15A)		163	A	C	86.78
2	179.73	DOCK11	DHR3	536	Cdc42(Q61L);Cdc42(G15A)		163	A	C	83.163
2	47.63	DOCK11	DHR3	572	Cdc42(G15A);Cdc42(Q61L)		131	A	E	63.011
2	38.19	DOCK11	DHR3	576	Cdc42(G15A);Cdc42(Q61L)		131	A	E	62.844
5	106.84	DOCK11	ARM	1157	Cdc42(G15A);Cdc42(Q61L)		150	B	C	70.207
1	97.64	DOCK11	ARM	1157	Cdc42(Q61L);Cdc42(G15A)		150	A	E	80.04
6	143	DOCK11	ARM	1219	Cdc42(G15A);Cdc42(Q61L)		163	A	C	Not modeled
1	134.68	DOCK11	ARM	1219	Cdc42(Q61L);Cdc42(G15A)		163	A	E	Not modeled
3	38	DOCK11	ARM	1233	Cdc42(G15A);Cdc42(Q61L)		150	A	C	Not modeled
1	74.89	DOCK11	ARM	1233	Cdc42(Q61L);Cdc42(G15A)		150	A	C	Not modeled
14	103.83	DOCK11	ARM	1233	Cdc42(G15A);Cdc42(Q61L)		163	A	C	Not modeled
4	72.55	DOCK11	ARM	1342	Cdc42(G15A);Cdc42(Q61L)		128	A	C	60.397
1	75.69	DOCK11	ARM	1342	Cdc42(G15A);Cdc42(Q61L)		163	A	C	41.11
1	55.13	DOCK11	ARM	1517	Cdc42(Q61L);Cdc42(G15A)		150	A	C	88.127
11	100	DOCK11	DHR2	1801	Cdc42(G15A);Cdc42(Q61L)		131	A	C	37.838
3	100.21	DOCK11	DHR2	1801	Cdc42(Q61L);Cdc42(G15A)		131	A	C	37.838
2	85.52	DOCK11	DHR2	1801	Cdc42(G15A);Cdc42(Q61L)		150	A	C	36.06
2	112.31	DOCK11	DHR2	1801	Cdc42(Q61L);Cdc42(G15A)		150	A	C	36.06
34	197.65	DOCK11	DHR2	1813	Cdc42(G15A);Cdc42(Q61L)		163	A	C	15.095
9	174.21	DOCK11	DHR2	1813	Cdc42(Q61L);Cdc42(G15A)		163	A	C	15.095
2	70.86	DOCK11	DHR2	1824	Cdc42(G15A);Cdc42(Q61L)		131	A	C	39.636
1	38.19	DOCK11	DHR2	1824	Cdc42(Q61L);Cdc42(G15A)		131	A	C	39.636
1	66.56	DOCK11	DHR2	1851	Cdc42(Q61L);Cdc42(G15A)		150	A	C	51.09
5	83.38	DOCK11	DHR2	1874	Cdc42(G15A);Cdc42(Q61L)		128	A	C	25.607
2	45.95	DOCK11	DHR2	1874	Cdc42(Q61L);Cdc42(G15A)		128	A	C	25.607
1	38	DOCK11	DHR2	1979	Cdc42(Q61L);Cdc42(G15A)		150	A	C	55.644
12	114.6	Cdc42(G15A)		16	DOCK11	DHR2	1874	C	A	22.031

7	124.08	Cdc42(G15A);Cdc42(Q61L)	107	DOCK11	DHR2	2006	C	A	22.467
4	67.12	Cdc42(G15A);Cdc42(Q61L)	133	DOCK11	AWS1	36	C	B	67.463
1	74.13	Cdc42(Q61L);Cdc42(G15A)	133	DOCK11	AWS1	36	E	A	74.677
1	61.3	Cdc42(G15A);Cdc42(Q61L)	133	DOCK11	ARM	1519	C	A	94.259
1	87.32	Cdc42(G15A);Cdc42(Q61L)	133	DOCK11	DHR2	1824	C	A	43.457
1	50.61	Cdc42(Q61L);Cdc42(G15A)	133	DOCK11	DHR2	1824	C	A	43.457
1	41.24	Cdc42(G15A);Cdc42(Q61L)	144	DOCK11	DHR3	573	E	A	59.376
1	44.31	Cdc42(G15A);Cdc42(Q61L)	150	DOCK11	DHR3	572	E	A	53.371
3	130.77	Cdc42(G15A);Cdc42(Q61L)	163	DOCK11	AWS1	53	E	A	74.421
2	88.73	Cdc42(Q61L);Cdc42(G15A)	163	DOCK11	AWS1	53	E	A	74.421
1	41.96	Cdc42(G15A);Cdc42(Q61L)	163	DOCK11	ARM	983	E	A	67.474
4	43.07	Cdc42(G15A);Cdc42(Q61L)	163	DOCK11	ARM	1157	C	A	75.04
4	136.44	Cdc42(G15A);Cdc42(Q61L)	163	DOCK11	ARM	1349	C	A	Not modeled
2	52.72	Cdc42(G15A);Cdc42(Q61L)	163	DOCK11	ARM	1517	C	A	69.701
226	132.11	Cdc42(G15A);Cdc42(Q61L)	163	DOCK11	DHR2	1801	C	A	12.859
66	120.06	Cdc42(Q61L);Cdc42(G15A)	163	DOCK11	DHR2	1801	C	A	12.859
4	56.47	Cdc42(G15A);Cdc42(Q61L)	163	DOCK11	DHR2	1851	C	A	37.963
2	76.27	Cdc42(Q61L);Cdc42(G15A)	163	DOCK11	DHR2	1851	C	A	37.963
1	61.12	Cdc42(Q61L);Cdc42(G15A)	163	DOCK11	DHR2	1984	C	A	43.284
6	50.61	Cdc42(G15A);Cdc42(Q61L)	166	DOCK11	DHR2	1788	C	A	15.384
19	58.66	Cdc42(G15A);Cdc42(Q61L)	166	DOCK11	DHR2	1801	C	A	17.306
2	53.47	Cdc42(Q61L);Cdc42(G15A)	166	DOCK11	DHR2	1801	C	A	17.306
24	71.85	Cdc42(G15A);Cdc42(Q61L)	94	Cdc42(G15A);Cdc42(Q61L)		135	C	C	17.632
2	41.65	Cdc42(Q61L);Cdc42(G15A)	94	Cdc42(Q61L);Cdc42(G15A)		135	E	E	18.082
13	53.17	Cdc42(G15A);Cdc42(Q61L)	94	Cdc42(G15A);Cdc42(Q61L)		144	C	C	12.727
8	35.65	Cdc42(G15A);Cdc42(Q61L)	94	Cdc42(G15A);Cdc42(Q61L)		150	C	C	11.216
24	97.87	Cdc42(G15A);Cdc42(Q61L)	107	Cdc42(G15A);Cdc42(Q61L)		150	C	C	10.482
4	119.21	Cdc42(G15A);Cdc42(Q61L)	133	Cdc42(G15A);Cdc42(Q61L)		128	C	C	8.919
3	141.98	Cdc42(Q61L);Cdc42(G15A)	133	Cdc42(Q61L);Cdc42(G15A)		128	E	E	8.894
1	62.09	Cdc42(G15A);Cdc42(Q61L)	133	Cdc42(G15A);Cdc42(Q61L)		150	C	C	27.979
7	135.16	Cdc42(G15A);Cdc42(Q61L)	133	Cdc42(G15A);Cdc42(Q61L)		163	C	C	28.757
3	89.14	Cdc42(Q61L);Cdc42(G15A)	133	Cdc42(Q61L);Cdc42(G15A)		163	E	E	27.606
11	100.21	Cdc42(G15A);Cdc42(Q61L)	135	Cdc42(G15A);Cdc42(Q61L)		131	C	C	9.522
6	65.44	Cdc42(Q61L);Cdc42(G15A)	135	Cdc42(Q61L);Cdc42(G15A)		131	C	C	9.988
27	94.27	Cdc42(G15A);Cdc42(Q61L)	144	Cdc42(G15A);Cdc42(Q61L)		150	E	E	9.744
4	69.64	Cdc42(Q61L);Cdc42(G15A)	144	Cdc42(Q61L);Cdc42(G15A)		150	C	C	9.988
16	100.45	Cdc42(G15A);Cdc42(Q61L)	144	Cdc42(G15A);Cdc42(Q61L)		163	C	C	21.952
6	92.92	Cdc42(Q61L);Cdc42(G15A)	144	Cdc42(Q61L);Cdc42(G15A)		163	E	E	21.466
1	70.86	Cdc42(G15A);Cdc42(Q61L)	150	Cdc42(G15A);Cdc42(Q61L)		131	E	F	46.711
1	76.73	Cdc42(Q61L);Cdc42(G15A)	150	Cdc42(Q61L);Cdc42(G15A)		131	F	E	46.354
3	160.91	Cdc42(G15A);Cdc42(Q61L)	153	Cdc42(G15A);Cdc42(Q61L)		163	C	C	17.356
3	207.86	Cdc42(Q61L);Cdc42(G15A)	153	Cdc42(Q61L);Cdc42(G15A)		163	E	E	17.344
4	77.53	Cdc42(G15A);Cdc42(Q61L)	163	Cdc42(G15A);Cdc42(Q61L)		131	C	C	28.727
1	76.42	Cdc42(Q61L);Cdc42(G15A)	163	Cdc42(Q61L);Cdc42(G15A)		131	E	E	27.73
3	81.63	Cdc42(G15A);Cdc42(Q61L)	163	Cdc42(G15A);Cdc42(Q61L)		135	C	C	22.157
1	81.63	Cdc42(Q61L);Cdc42(G15A)	163	Cdc42(Q61L);Cdc42(G15A)		135	E	E	21.021
8	77.93	Cdc42(G15A);Cdc42(Q61L)	163	Cdc42(G15A);Cdc42(Q61L)		150	C	C	25.14
2	77.93	Cdc42(Q61L);Cdc42(G15A)	163	Cdc42(Q61L);Cdc42(G15A)		150	E	E	24.775
3	40.3	Cdc42(G15A);Cdc42(Q61L)	166	Cdc42(G15A);Cdc42(Q61L)		150	C	C	22.803
12	74.13	Cdc42(G15A);Cdc42(Q61L)	166	Cdc42(G15A);Cdc42(Q61L)		163	C	C	7.971
1	55.53	Cdc42(Q61L);Cdc42(G15A)	166	Cdc42(Q61L);Cdc42(G15A)		163	E	E	7.831
2	42.1	Cdc42(Q61L);Cdc42(G15A)	166	Cdc42(Q61L)		5	E	E	16.461

SI References

1. T. Terada, T. Murata, M. Shirouzu, S. Yokoyama, Cell-free expression of protein complexes for structural biology. *Methods Mol. Biol.* **1091**, 151–159 (2014).
2. Katsura, K. et al. A reproducible and scalable procedure for preparing bacterial extracts for cell-free protein synthesis. *J. Biochem.* **162**, 357–369 (2017).
3. M. Kukimoto-Niino et al., Structural Basis for the Dual Substrate Specificity of DOCK7 Guanine Nucleotide Exchange Factor. *Structure* **27**, 741–748 (2019).
4. Mukai, T. et al. Adding L-lysine derivatives to the genetic code of mammalian cells with engineered pyrrolysyl-tRNA synthetases. *Biochem. Biophys. Res. Commun.* **371**, 818–822 (2008).
5. T. Shinoda et al., Conformational alteration of DOCK5•ELMO1 signalosome on lipid membrane. *Communs. Biol.* **8**, 1523 (2025).
6. A. Punjani, J. L. Rubinstein, D. J. Fleet, M. A. Brubaker, cryoSPARC: algorithms for rapid unsupervised cryo-EM structure determination. *Nat. Methods* **14**, 290–296 (2017).
7. R. Sanchez-Garcia, J. Gomez-Blanco, A. Cuervo C. O. S. Sorzano, J. Vargas, J. DeepEMhancer: a deep learning solution for cryo-EM volume post-processing. *Commun Biol.* **4**, 874 (2021).
8. J. Jumper et al., Highly accurate protein structure prediction with AlphaFold. *Nature* **596**, 583–589 (2021).
9. E. C. Meng et al., UCSF ChimeraX: tools for structure building and analysis. *Prot. Sci.* **32**, e4792 (2023).
10. P. Emsley, B. Lohkamp, W. G. Scott, K. Cowtan, Features and development of *Coot*. *Acta Crystallogr.* **D66**, 486–501 (2010).
11. D. Liebschner et al., Macromolecular structure determination using X-rays, neutrons and electrons: recent developments in Phenix. *Acta Crystallogr*. **D75**, 861–877 (2019).
12. J. Jung et al., GENESIS 2.1: High-performance molecular dynamics software for enhanced sampling and free-energy calculations for atomistic, coarse-grained, and quantum mechanics/molecular mechanics models. *J. Phys. Chem. B* **128**, 6028–6048 (2024).
13. O. Miyashita, C. Kobayashi, T. Mori, Y. Sugita, F. Tama, Flexible fitting to cryo-EM density map using ensemble molecular dynamics simulations. *J. Comput. Chem.* **38**, 1447–1461 (2017).
14. T. Mori et al., Acceleration of cryo-EM flexible fitting for large biomolecular systems by efficient space partitioning. *Structure* **27**, 161–174 (2019).
15. V. B. Chen et al., MolProbity: all-atom structure validation for macromolecular crystallography. *Acta Crystallogr*. **D66**, 12–21 (2010).
16. Schrödinger, LLC. The PyMOL Molecular Graphics System, Version 3.0.
17. F. Medina et al., Activated RhoA is a positive feedback regulator of the Lbc family of Rho guanine nucleotide exchange factor proteins. *J. Biol. Chem.* **288**, 11325–11333 (2013).
18. K. Robbe, A, Otto-Bruc, P. Chardin, B. Antonny, Dissociation of GDP dissociation inhibitor and membrane translocation are required for efficient activation of Rac by the Dbl homology-pleckstrin homology region of Tiam. *J. Biol. Chem.* **278**, 4756–4762 (2003).
19. L. Holm, Dali server: structural unification of protein families. *Nucl. Acid Res.* **50**, W210–W215 (2022).
